# Recent Advances in the Synthesis of β-Carboline Alkaloids

**DOI:** 10.3390/molecules26030663

**Published:** 2021-01-27

**Authors:** Tímea Szabó, Balázs Volk, Mátyás Milen

**Affiliations:** Egis Pharmaceuticals Plc., Directorate of Drug Substance Development, P.O. Box 100, H-1475 Budapest, Hungary; szabo.timea91@gmail.com (T.S.); volk.balazs@egis.hu (B.V.)

**Keywords:** β-carboline, natural products, total synthesis, bioactive molecules

## Abstract

β-Carboline alkaloids are a remarkable family of natural and synthetic indole-containing heterocyclic compounds and they are widely distributed in nature. Recently, these alkaloids have been in the focus of interest, thanks to their diverse biological activities. Their pharmacological activity makes them desirable as sedative, anxiolytic, hypnotic, anticonvulsant, antitumor, antiviral, antiparasitic or antimicrobial drug candidates. The growing potential inherent in them encourages many researchers to address the challenges of the synthesis of natural products containing complex β-carboline frameworks. In this review, we describe the recent developments in the synthesis of β-carboline alkaloids and closely related derivatives through selected examples from the last 5 years. The focus is on the key steps with improved procedures and synthetic approaches. Furthermore the pharmacological potential of the alkaloids is also highlighted.

## 1. Introduction

**Figure 1:**
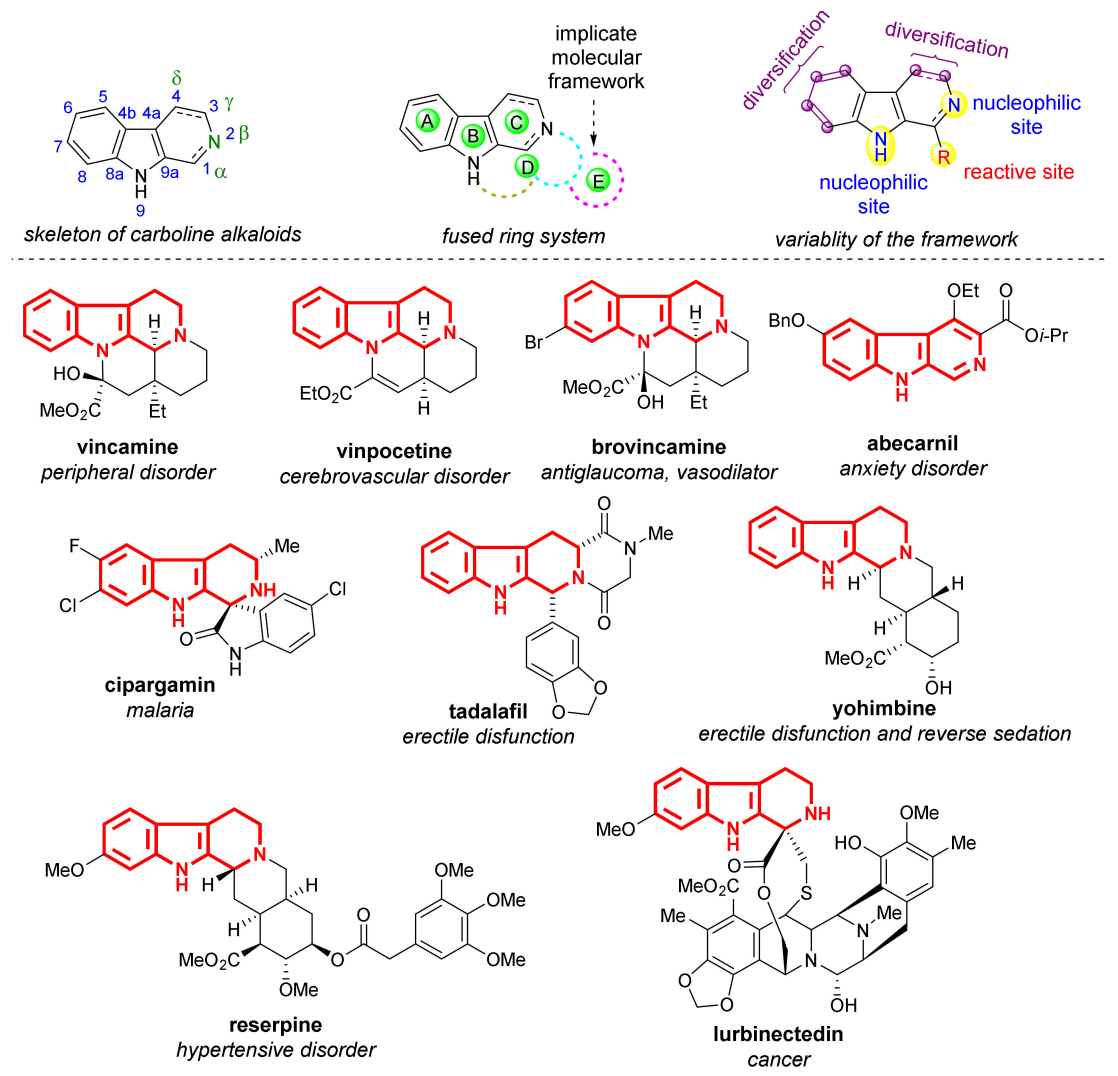
Structure of carboline skeleton and drugs containing a β-carboline framework.

Carbolines are a remarkable family of heterocyclic natural products, with outstanding pharmacological potential. They are determined by their tricyclic, pyridine-fused indole framework (where the rings are identified as A, B and C), and classified according to the degree of saturation (fully saturated: 1,2,3,4-tetrahydro; partially saturated: 3,4-dihydro; and unsaturated β-carbolines) and the position of the N-atom in the C-ring as α-, β-, γ- or δ-carbolines (Figure 1) [1,2]. β-Carboline alkaloids are widely distributed in nature including various plants, foodstuffs, marine creatures, insects, mammals as well as human tissues and body fluids. Numerous representatives of this family show various biological activities [3,4,5]. The fascinating diversity of structures and medicinal potential [6] inherent in them encourage several researchers to deal with the synthesis of β-carboline containing natural products and their synthetic derivatives [2,4,7,8,9]. Several commercial drugs or drug candidates such as vinpocetine, brovincamine, abecarnil, cipargamin, tadalafil, reserpine and lurbinectedin contain this unique framework (Figure 1).

This review focuses on the field of β-carboline research through selected examples from the last 5 years, where the key information on natural occurrence, structural diversity and biological activities is highlighted (Table 1). The comprehensive demonstration of recent advances in the synthesis of naturally occurring β-carbolines, including both simple β-carbolines (Section 2 of this review) and fused ring systems containing this framework (see Figure 1 and Section 3 of this review), is discussed with a special emphasis on the innovative framework construction steps.

## 2. Natural Products Containing a Simple β-Carboline Skeleton

**Scheme 1:**
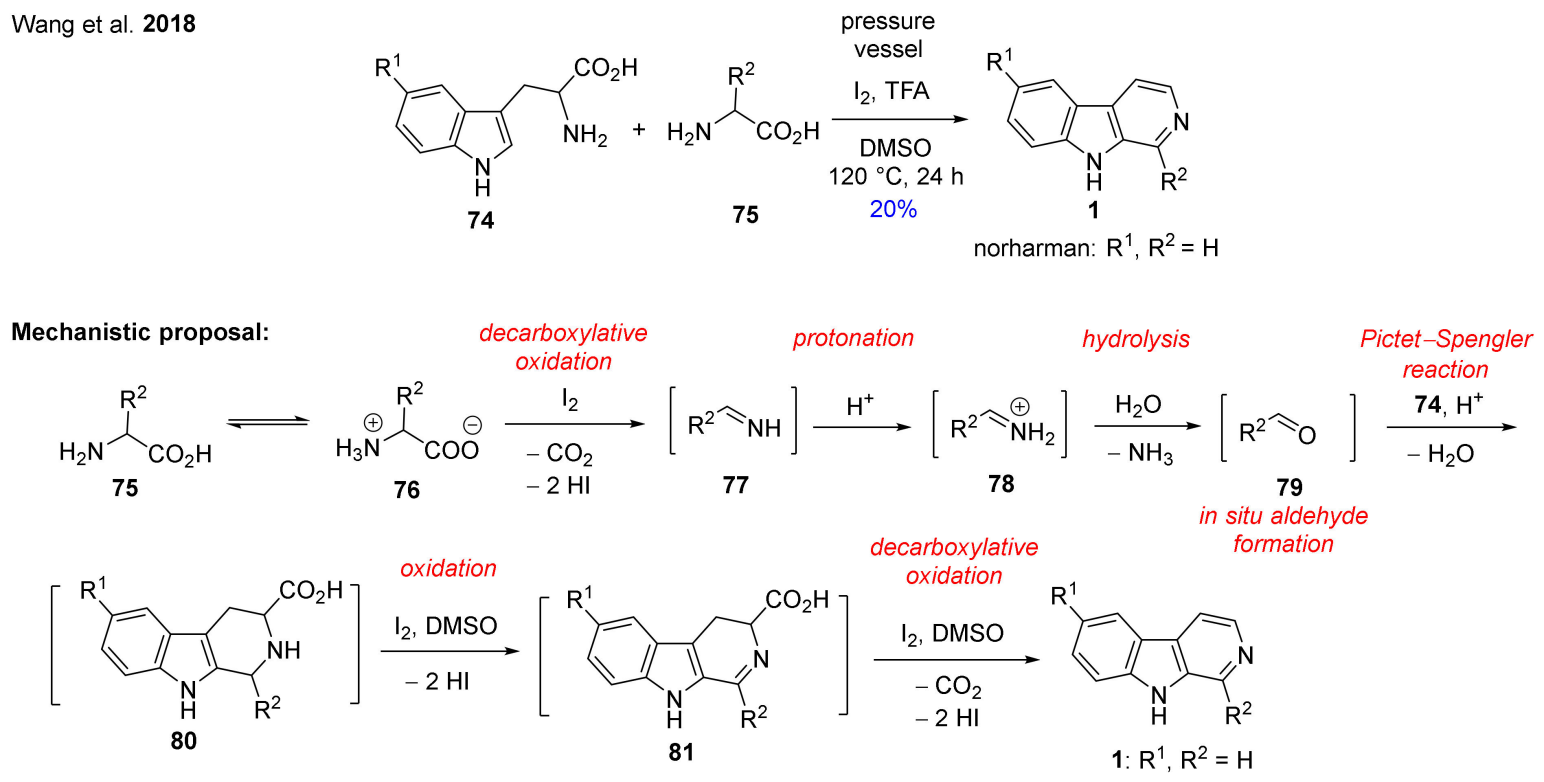
One-pot synthesis of natural product norharman (**1**) and further β-carboline derivatives.

A novel one-pot synthesis of norharman (**1**) and 1- or 6-substituted β-carbolines have been achieved by Wang and co-workers (Scheme 1) [109]. The designed synthesis starting from racemic tryptophan (**74**) and various amino acids (**75**) proceeds through sequential steps, driven by a reaction with I_2_ and TFA, followed by decarboxylation, deamination, Pictet–Spengler reaction and oxidation. By using various amino acids, further alkaloids and synthetic analogues were also obtained.

**Scheme 2:**
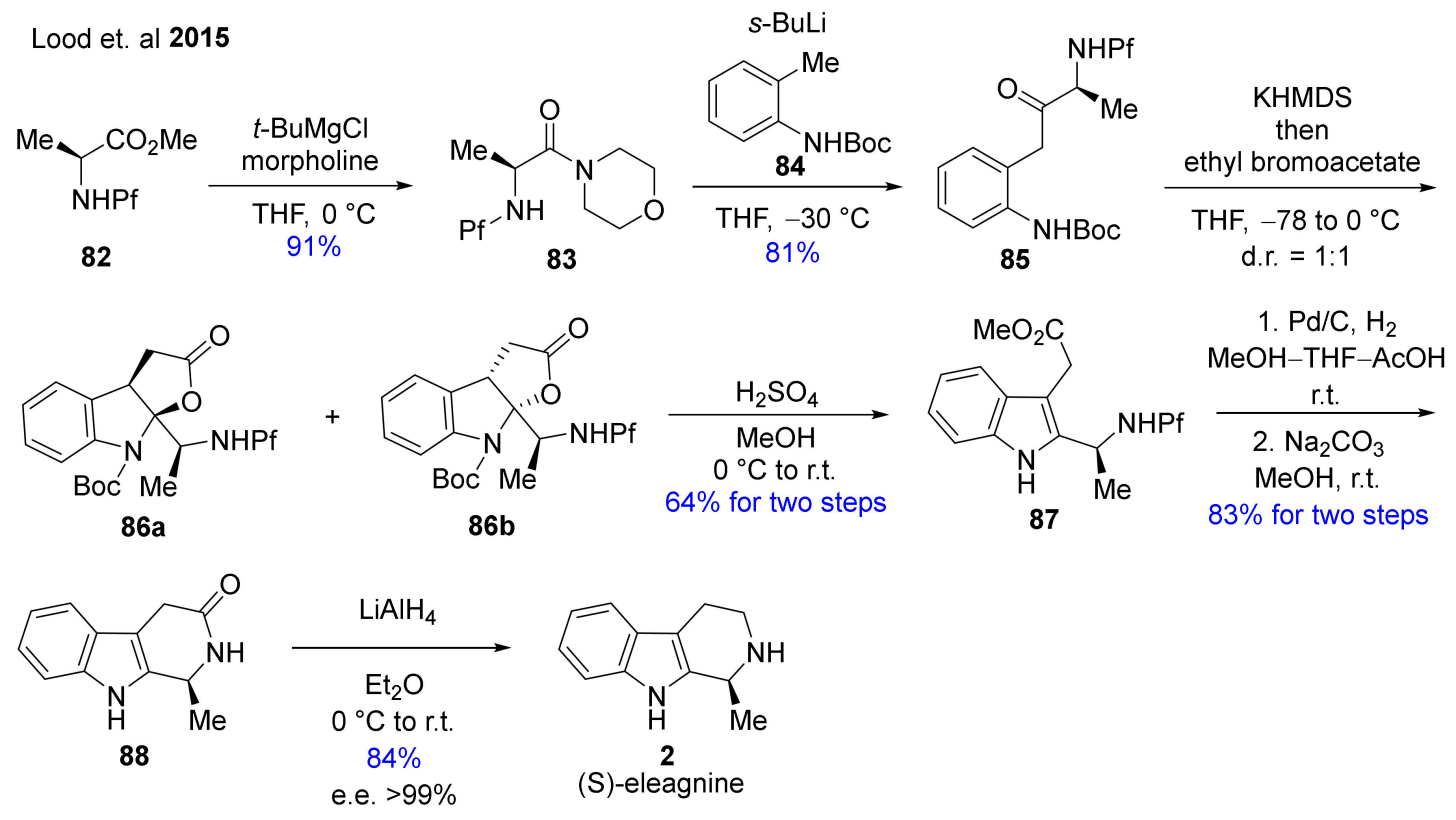
Enantioselective synthesis of (*S*)-eleagnine (**2**).

In 2015, Lood et al. reported the new enantiospecific gram-scale synthesis of (*S*)-eleagnine/(−)-tetrahydroharmane (**2**) (Scheme 2) [110]. Their synthesis started from the previously modified amino acid methyl ester **82**, which included the appropriate chirality. The treatment of **82** with morpholine in the presence of *t*-BuMgCl resulted in morpholine amide **83**. Amide **83** went through a subsequent lateral lithiation, which was followed by the addition of Boc-protected *o*-toluidine (**84**) to furnish ketone **85** in gram scale. After that, ketone **85** was treated with KHMDS, then the potassium enolate formed was quenched with ethyl bromoacetate, which resulted in a diastereomeric mixture of indoline γ-lactones (**86a** and **86b**). Treating this diastereomeric mixture with sulfuric acid in MeOH facilitated a sequential lactone ring opening and Boc-cleavage to form indole **87**. Hydrogenolysis of Pf-protecting group and treatment of the free amine with Na_2_CO_3_ base led to lactam **88**. As a final step, LiAlH_4_ reducing agent was applied on **88** to provide (*S*)-eleagnine (**2**) in a gram-scale synthesis with an enantiomeric excess (e.e.) >99%.

**Scheme 3:**
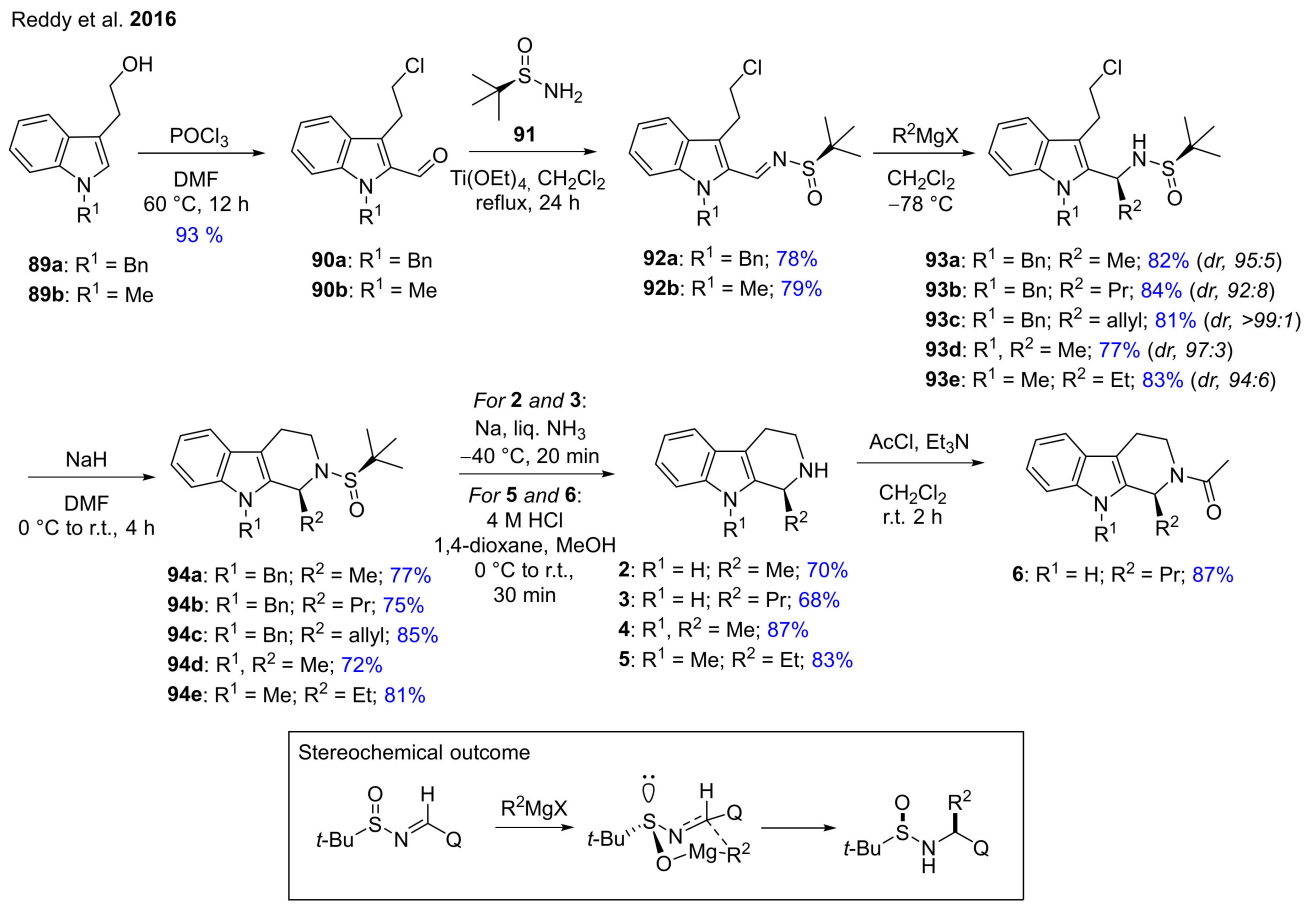
Synthesis of (−)-tetrahydroharman (**2**), (−)-komaroidine (**3**), *N*-(+)-methyltetrahydroharman (**4**), (+)-1-ethyl-9-methyltetrahydro-β-carboline (**5**) and (+)-*N*-acetylkomaroidine (**6**).

Reddy et al. have accomplished the stereoselective synthesis of natural compounds (−)-tetrahydroharman (**2**), (−)-komaroidine (**3**), *N*-tetrahydroharman (**4**), (+)-1-ethyl-9-methyltetrahydro-β-carboline (**5**) and (+)-*N*-acetylkomaroidine (**6**) using Ellman’s sulfinamide [(*R*)-*tert*-butanesulfinamide, **91**] [111] as a chiral auxiliary generally used for the preparation of chiral amines (Scheme 3) [112]. The use of this readily available reagent as the source of asymmetric induction to establish the stereochemical configuration of these alkaloids makes this approach convenient and practical. *N*-Sulfinylimines **92** were prepared with the reaction of **90** and (*R*)-*tert*-butanesulfinamide (**91**) in the presence of Ti(OEt)_4_. The addition of alkyl-Grignard reagent to **92** led to the corresponding sulfinamides **93** with the expected stereochemical outcome and with high diastereoselectivity. In this reaction, the occupation of the less hindered equatorial position in the six-membered transitional state resulted in a favorable attack from the same face (Scheme 3). The synthesis was continued with a reductive or acidic removal of both the benzyl group and the sulfinyl auxiliary to afford products **2**–**5**. A final classical acetylation on the piperidine nitrogen atom was needed to prepare target molecule **6**.

**Scheme 4:**
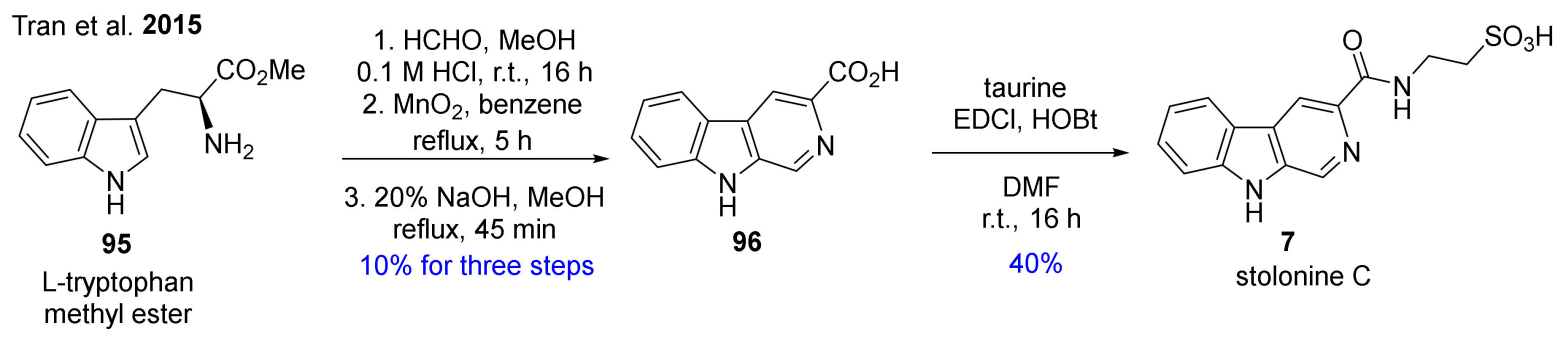
Synthesis of stolonine C (**7**).

The chemical study on the marine tunicate *Cnemidocarpa stolonifera* contributed to the discovery and synthesis of 3 new alkaloids, including a β-carboline alkaloid called stolonine C (**7**, Scheme 4) [20]. The synthesis starts from l-tryptophan methyl ester (**95**) viaPictet–Spengler reaction with formaldehyde followed by dehydrogenation of the C-ring and hydrolysis of the ester group. Intermediate **96** was transformed into an amide with taurine resulting in final product **7**.

**Scheme 5:**
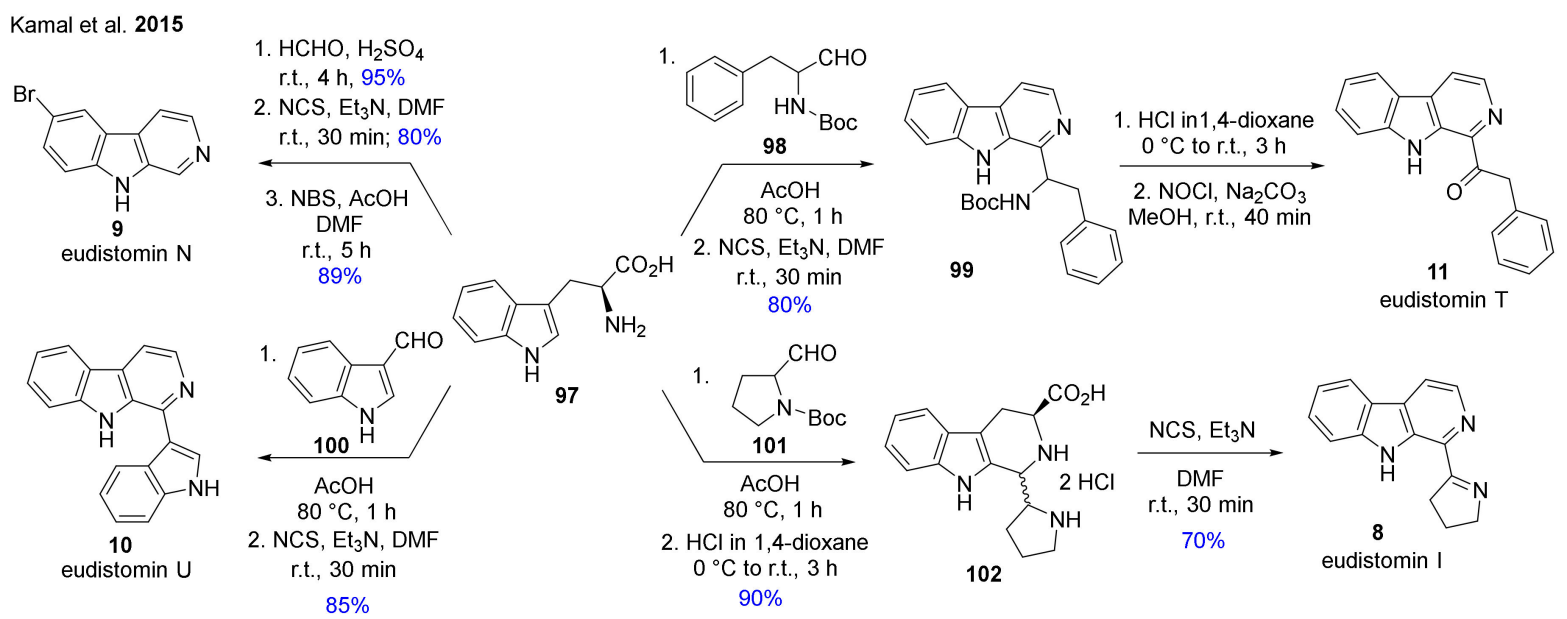
Kamal’s improved synthesis of eudistomins I (**8**), N (**9**), T (**10**) and U (**11**).

Eudistomins are important natural products belonging to the family of β-carboline alkaloids, and show a wide variety of biological activities [26,113,114,115]. Kamal et al. have developed an efficient method for the synthesis of diverse β-carboline derivatives and naturally occurring alkaloids such as eudistomins I, N, T and U (**8**, **9**, **10** and **11**, respectively) [116] which are isolated from ascidians (Scheme 5) [23,24,27,116,117]. The protocol is based on the Pictet‒Spengler reaction of l-tryptophan (**97**) and the corresponding aldehydes, subsequent decarboxylative aromatization of the C-ring employing NCS and variation of classical synthesis methods in the final steps, providing an easier and more convenient pathway compared to the previously reported methods. In addition, the most efficient synthesis of **10** has been described using a one-pot approach.

It is worth mentioning that the NBS-promoted oxidative dehydrogenation of the C-ring has also been devised and has become very popular recently [118]. The method also provides access to a variety of β-carbolines and can be applied effectively in generating molecule libraries.

**Scheme 6:**
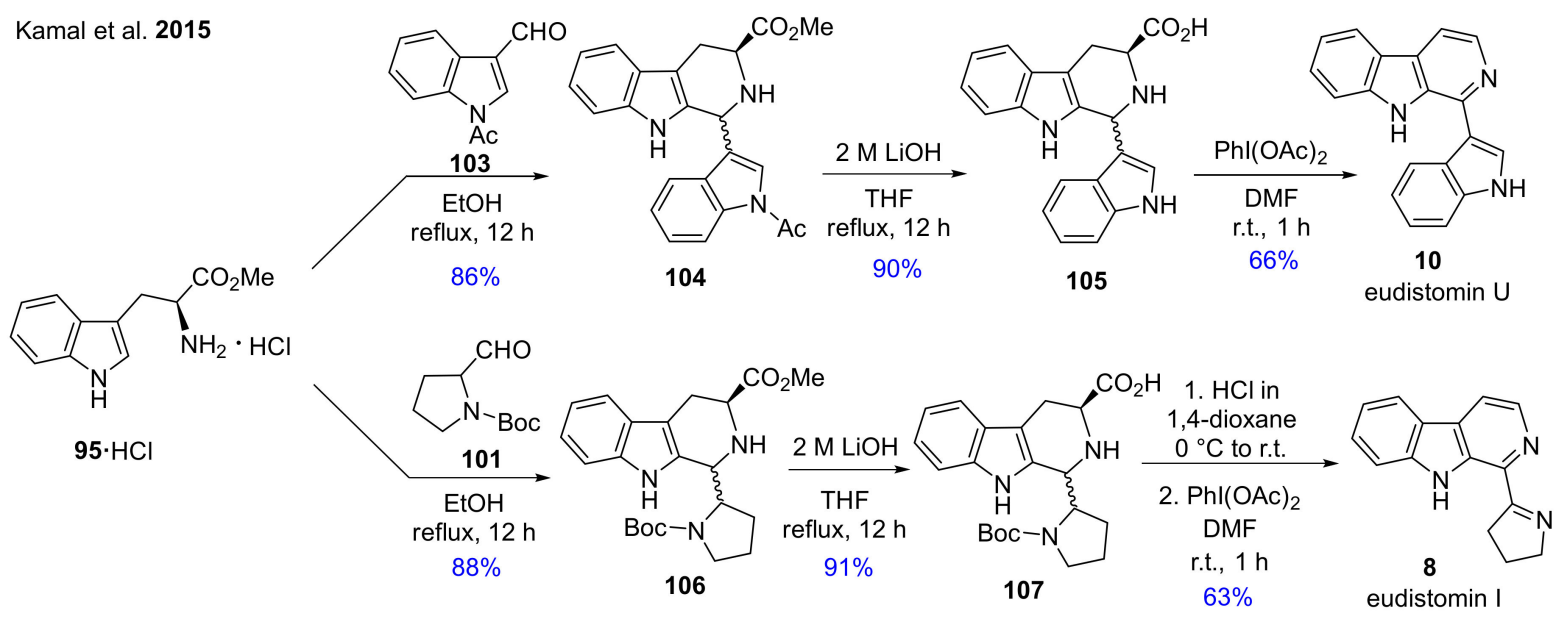
Kamal’s synthesis of eudistomins U (**10**) and I (**8**).

The beneficial pharmacological efficacy of eudistomins has prompted other researchers to further improve their total synthesis [115]. In 2015, also Kamal et al. reported a three-step synthesis of eudistomin U (**10**) starting from L-tryptophan methyl ester (**95 ∙ HCl**), which underwent a Pictet–Spengler condensation with *N*-acetylindole-3-carboxaldehyde (**103**) to form THβC ester **104** (Scheme 6). Ester hydrolysis and loss of the *N*-acetyl group of intermediate **104** provided carboxylic acid **105**. Compound **105** went through an iodobenzene diacetate-mediated oxidative decarboxylation to afford **10** in 51% overall yield (Scheme 5) [119]. Using this method, the authors also developed the synthesis of eudistomin I (**8**) [119], where iodobenzene diacetate mediates not only decarboxylation and aromatization of the THβC ring, but also the partial dehydrogenation of the pyrrolidine ring in a one-pot operation.

**Scheme 7:**
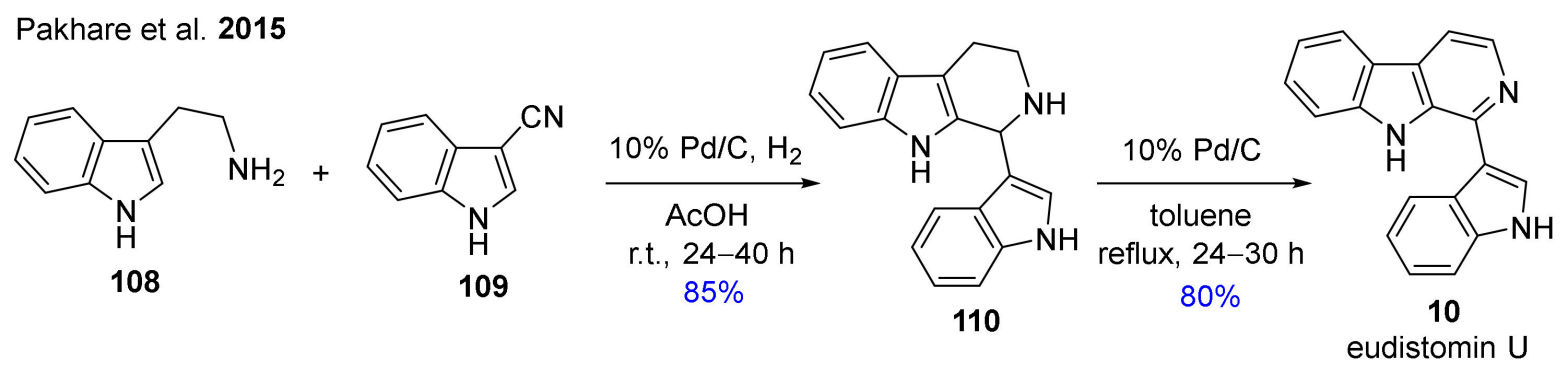
Synthesis of eudistomin U (**10**).

An alternative synthesis of **10** through the reaction of tryptamine (**108**) and indole-3-carbonitrile (**109**) followed by dehydrogenation of intermediate **110** with palladium on carbon was described in two steps with 68% overall yield (Scheme 7) [120].

**Scheme 8:**
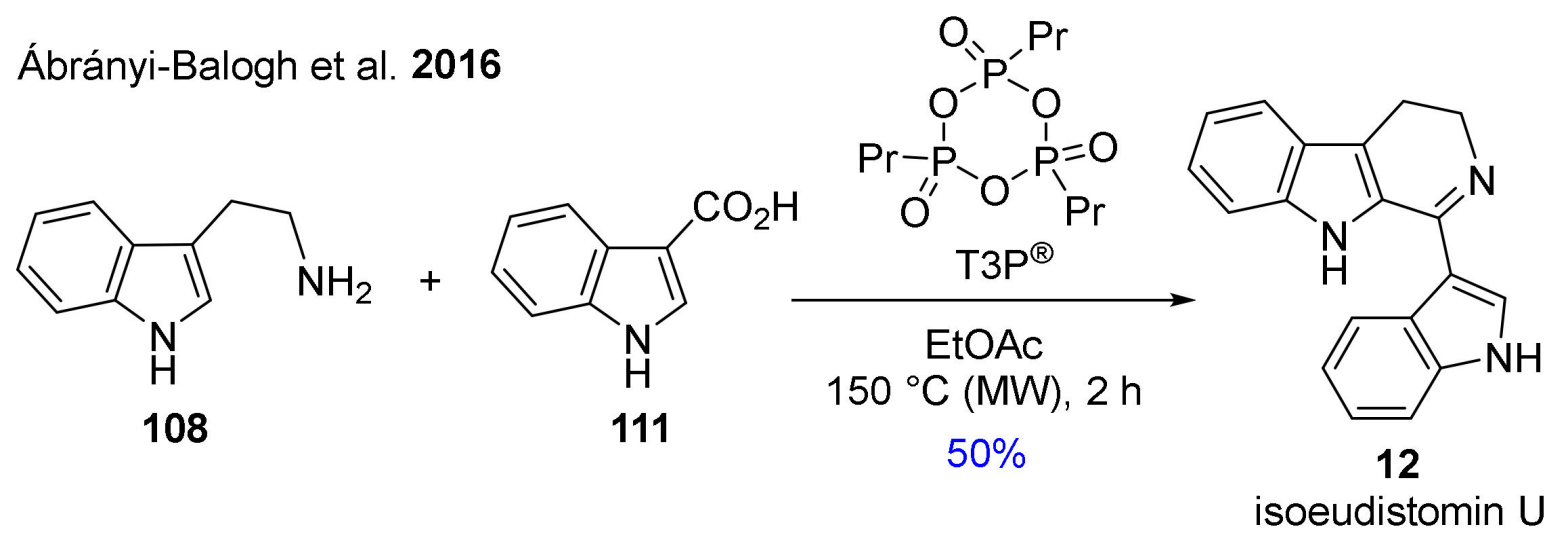
Ábrányi-Balogh’s synthesis of isoeudistomin U (**12**).

In 2016, Ábrányi-Balogh and co-workers developed a microwave-assisted one-pot protocol in the presence of T3P^®^ for the preparation of substituted 3,4-dihydro-β-carbolines (DHβCs). The new method was also applied for the synthesis of isoeudistomin U alkaloid (**12**) starting from tryptamine (**108**) and indole-3-carboxylic acid (**111**) with 50% yield (Scheme 8) [121].

**Scheme 9:**
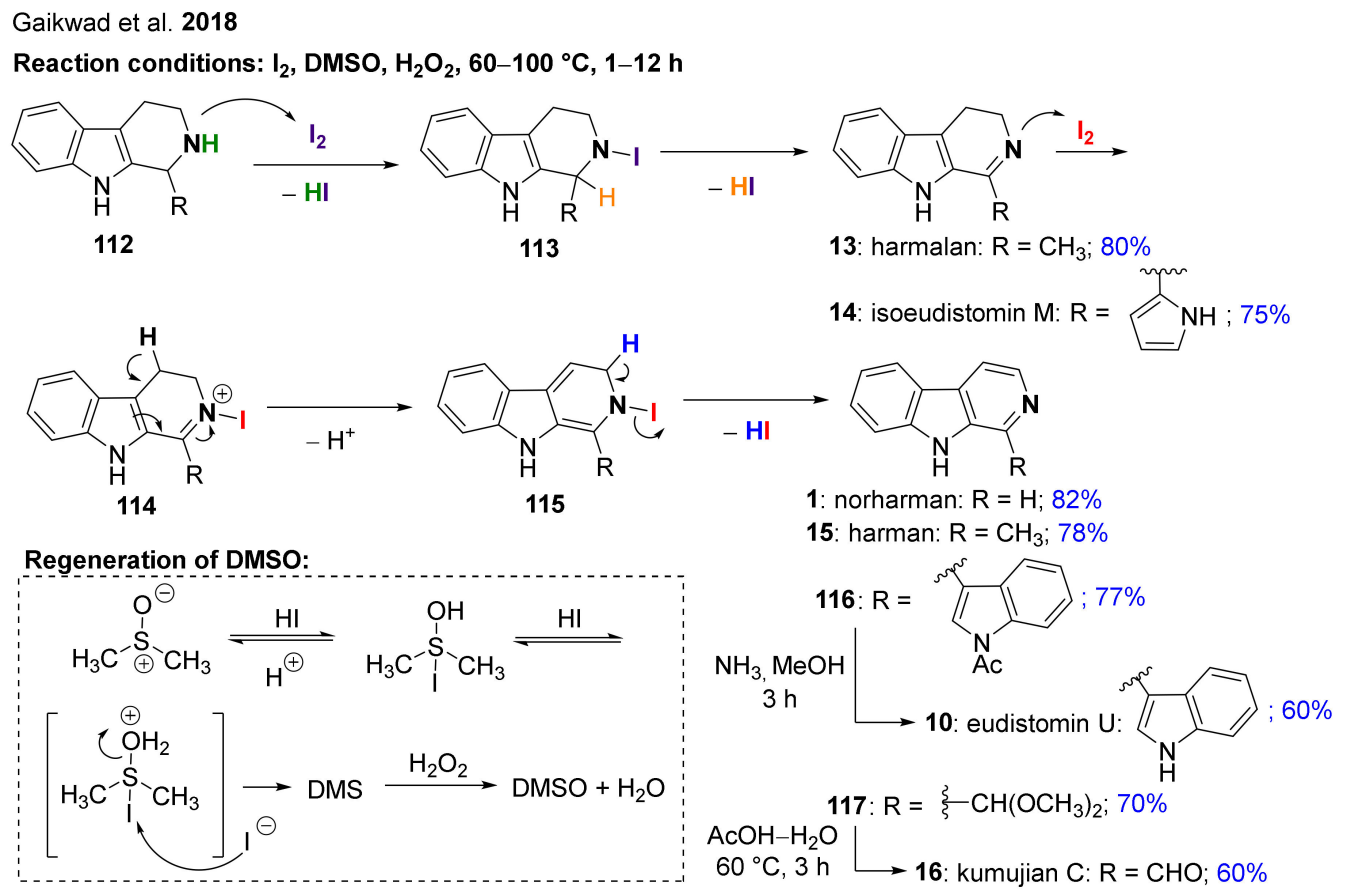
Synthesis of harmalan (**13**), isoeudistomin M (**14**), norharman (**1**), harman (**15**), eudistomin U (**10**) and kumujian C (**16**) via iodine-mediated dehydrogenation.

Gaikwad et al. have developed an iodine-mediated metal-free protocol for the chemoselective dehydrogenation and aromatization of THβCs into DHβCs and β-carbolines in the presence of H_2_O_2_ as the oxidant in DMSO (Scheme 9) [122]. The method was applicable not only for the preparation of variously substituted β-carbolines but also for that of bioactive natural products eudistomin U (**10**), harmalan (**13**), isoeudistomin M (**14**), norharman (**1**), harman (**15**) and kumujian C (**16**). The plausible reaction mechanism of the dearomatization protocol was also presented, which starts with a *N*-iodination followed by an *N*-dehydroiodination of THβCs **112** and **113**, respectively. These two steps result in the imine (**13** or **14**), which is then further transformed into an ammonium intermediate (**114**) by an additional *N*-iodination. After deprotonation and dehydro-iodination, the corresponding β-carboline (**1**, **15**, **116** or **117**) is formed. The generated HI reacts with DMSO to give molecular iodine and dimethyl sulfide (DMS). The role of H_2_O_2_ is to speed up the reaction by the oxidation of DMS back into DMSO. Intermediates **116** and **117** could then be transformed into eudistiomin U (**10**) or kumujian C (**16**) in the final step.

**Scheme 10:**
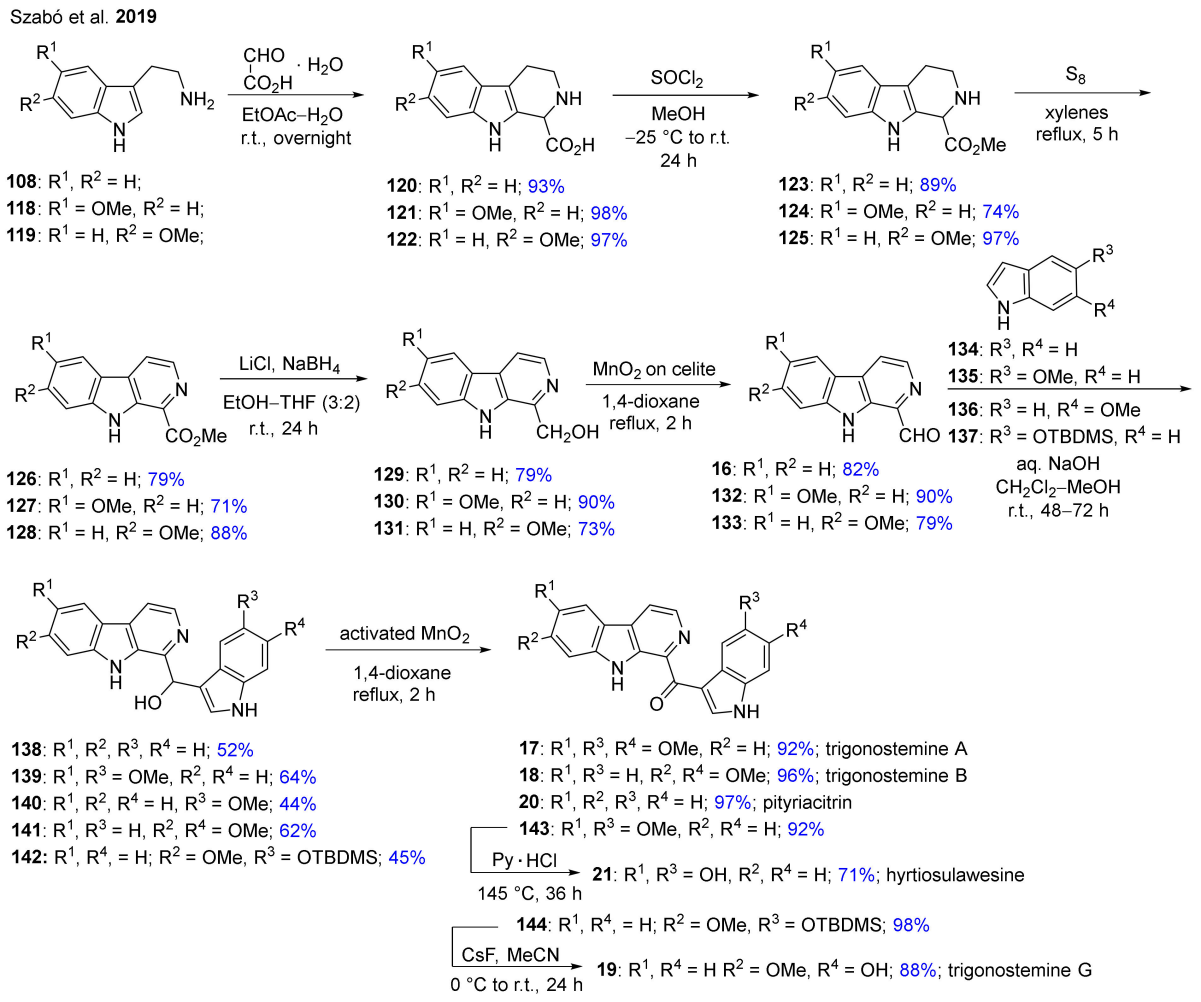
Total synthesis of trigonostemines A (**17**), B (**18**) and G (**19**) and a novel synthetic route for the preparation of pityriacitrin (**20**) and hyrtiosulawesine (**21**).

Szabó et al. have recently published the first total synthesis of alkaloids trigonostemine A, B and G (**17**–**19**) and the preparation of pityriacitrin (**20**) and hyrtiosulawesine (**21**) (Scheme 10) [123,124]. The appropriate key intermediates kumujian C (**16**) and its derivatives (**132**, **133**) were prepared via a novel and practical synthesis route. In the first step, the Pictet–Spengler reaction of tryptamines (**108**, **118**, **119**) and glyoxylic acid monohydrate gave THβC carboxylic acids **(120**–**122)**. This was followed by the esterification of compounds **120**–**122**. Aromatization of the C-ring was carried out in the presence of elemental sulfur. Afterwards the ester group of **126**–**128** was reduced to give alcohols. Finally, oxidation of the hydroxy group in alcohols **129**–**131** was achieved using MnO_2_ on Celite^®^, and aldehydes **16**, **132**, **133** were isolated in good yields. The synthesis of alkaloids **17**–**21** was then performed from these building blocks (**16**, **132**, **133**), which were reacted with indoles (**134**–**137**) to afford secondary alcohols and then oxidized with active MnO_2_ to provide the first total synthesis of trigonostemines A (**17**) and B (**18**), carried out in 7 steps. At the same time, this pathway represents a new synthetic method for the preparation of pityriacitrin (**20**). An additional step was needed to produce hyrtiosulawesine (**21**), where the target compound was obtained by *O*-demethylation of derivative **143** in pyridine hydrochloride. The first total synthesis of trigonostemine G (**19**) was performed in 8 steps analogously to the above method. The sensitive OH moiety of the indole was protected by a TBDMS group, which was removed under mild conditions in the final step with CsF (Scheme 10).

**Scheme 11:**
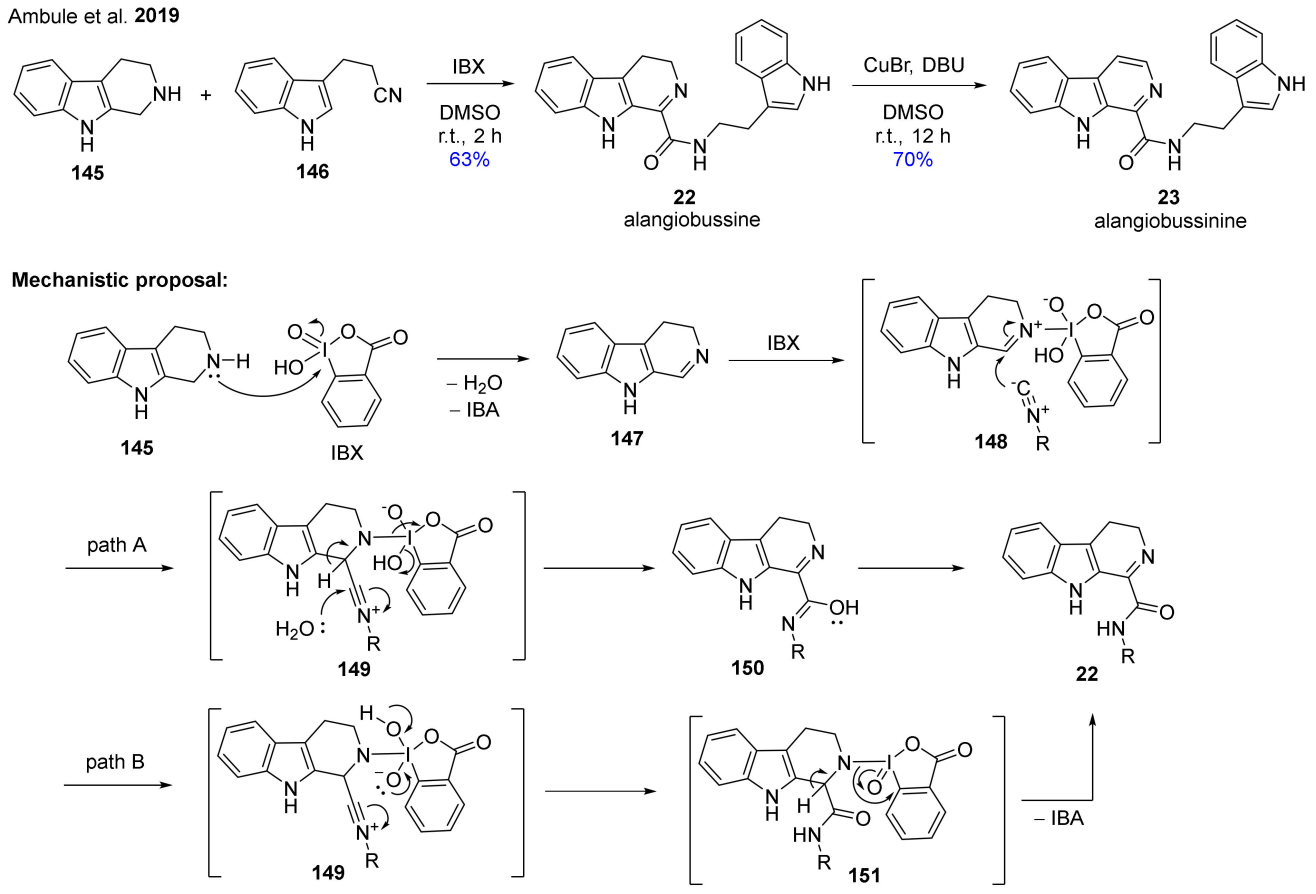
Synthesis of alangiobussine (**22**) and alangiobussinine (**23**).

The IBX-mediated total synthesis of alangiobussine (**22**) and alangiobussinine (**23**) was achieved through the oxidative addition of isocyanide **146** to tryptoline (**145**) [125]. The general method (Scheme 11) represents an efficient preparation for imino-carboxamides, where tetrahydroisoquinolines and tryptolines were reacted with isocyanides. The IBX-mediated oxidative addition protocol was successfully applied for the gram-scale synthesis of natural product alangiobussine (**22**), which was further transformed to alangiobussinine (**23**) through the dehydrogenation of the C-ring in the presence of CuBr and DBU. The plausible reaction mechanism was also investigated by isotopic labeling and real-time NMR experiments, which made two pathways probable.

**Scheme 12:**
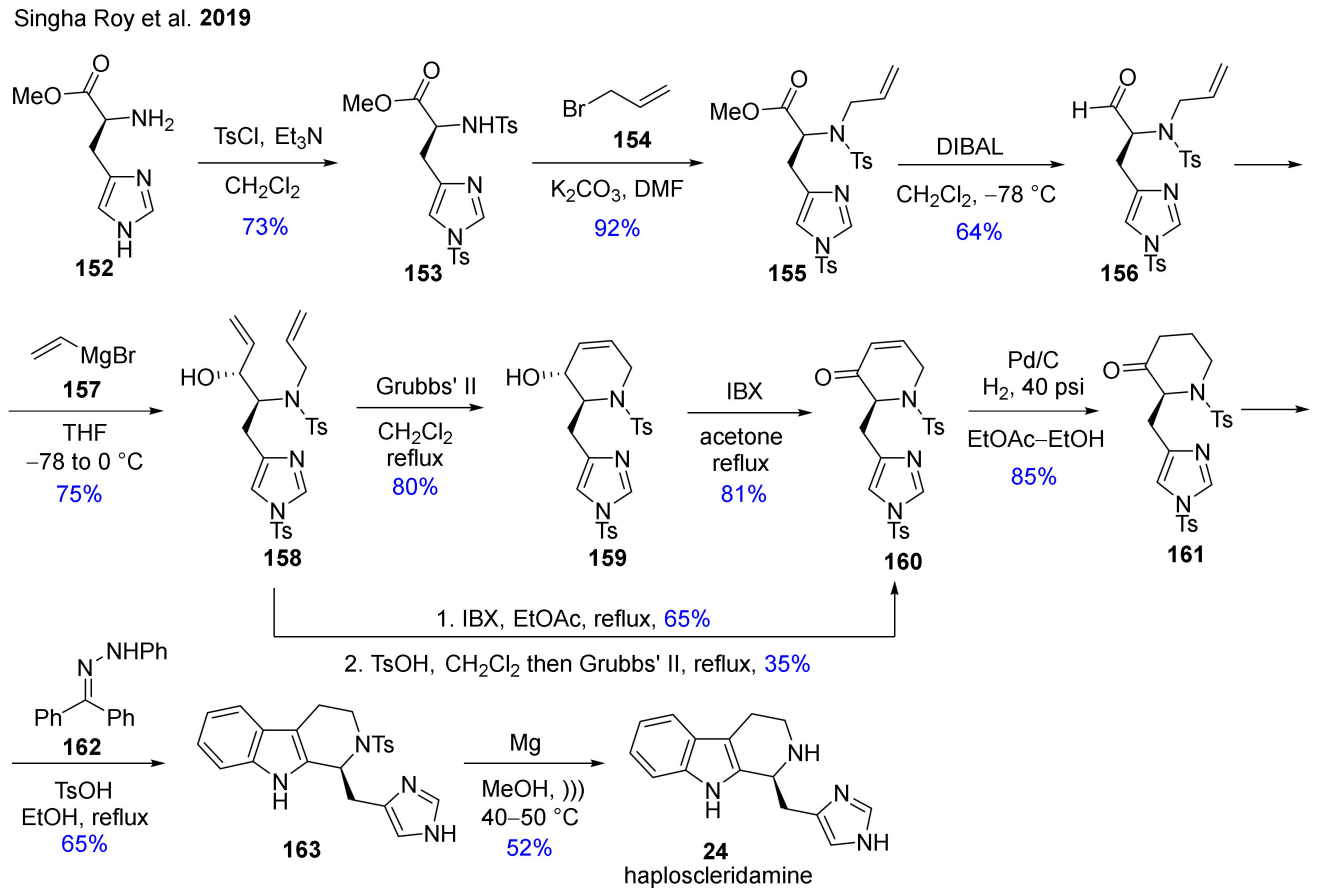
Synthesis of haploscleridamine (**24**).

The first asymmetric total synthesis of haploscleridamine (**24**) was accomplished from histidine (Scheme 12) [126]. The synthesis starts with the tosylation of histidine methyl ester (**152**), then *N*-allylation with allyl bromide (**154**) was applied on intermediate **153** leading to compound **155**, followed by a partial ester reduction to afford aldehyde **156**. After Grignard reaction with vinylmagnesium bromide (**159**), alcohol **158** was formed, which was transformed into tetrahydropyridine congener **159** via ring-closing metathesis. After oxidation of alcohol to ketone **160**, and reduction C=C double bond to intermediate **161**, indole formation took place via Buchwald modification of the classical Fischer indole synthesis to obtain **163**. Finally, **24** was synthesized by the reductive deprotection of **163** with Mg under ultrasonication conditions.

**Scheme 13:**
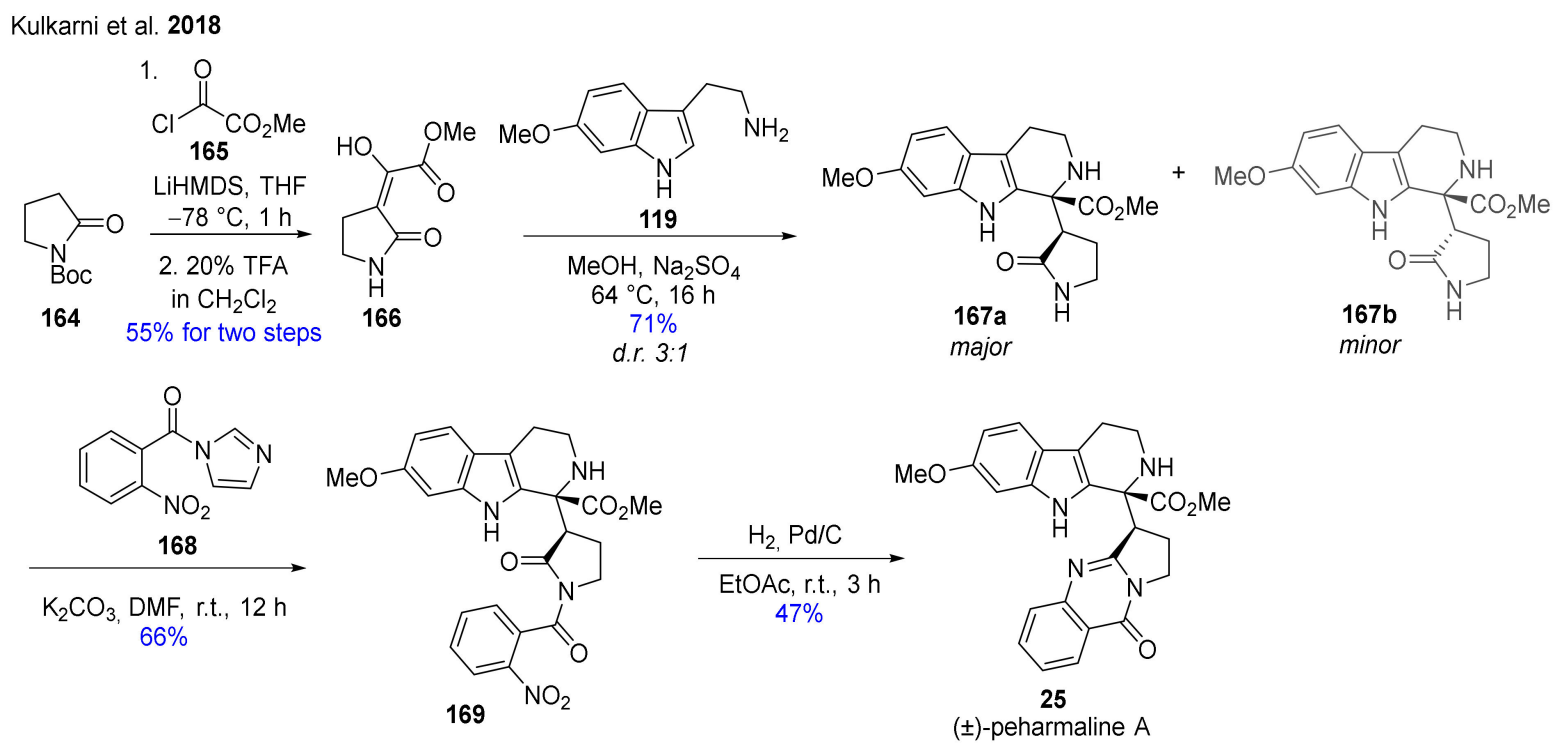
Synthesis of (±)-peharmaline A (**25**).

The first total synthesis of the potential anticancer agent, the β-carboline-vasicinone hybrid alkaloid (±)-peharmaline A (**25**) was accomplished in a short sequence starting from readily available starting materials (Scheme 13) [127]. Boc-protected pyrrolidone **164** was reacted with acyl chloride **165** in the presence of LiHMDS. After treatment with TFA, the compound thus formed (**166**) was reacted with 6-methoxytryptamine (**119**). The Pictet–Spengler reaction did not take place in a stereoselective manner: besides major diastereomer **167a**, minor product **167b** was also obtained. However, the inversion of the chiral center was observed in the minor diastereomer **167b** during the next step. Natural product **25** was synthesized by *N*-acylation of the mixture of compounds **167a** and **167b** with reagent **168** and subsequent catalytic hydrogenation of nitro derivative **169**.

**Scheme 14:**
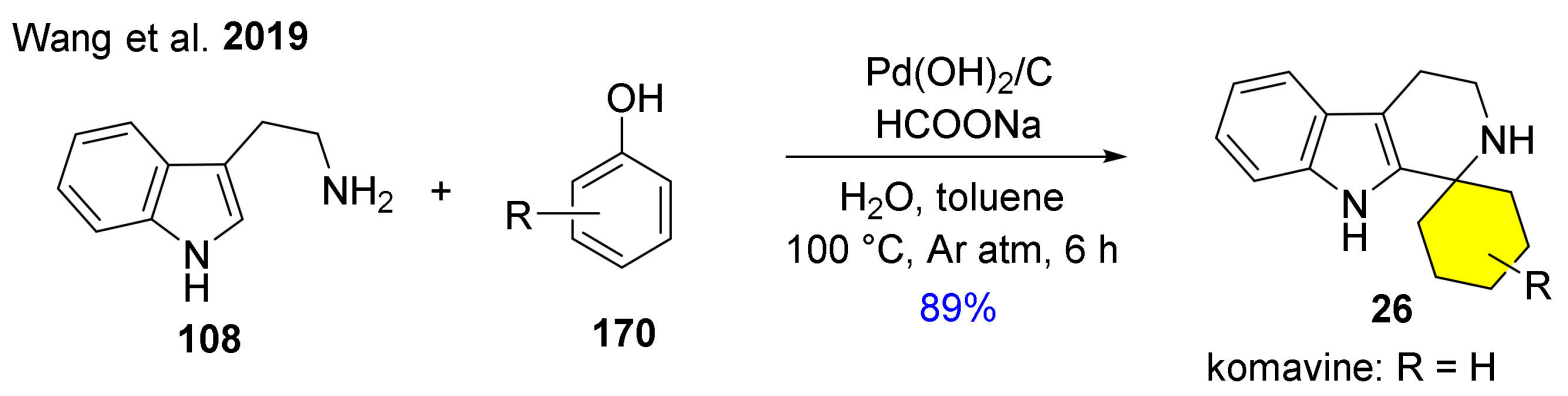
Pd-catalyzed cross-annulation of phenols (**170**) and tryptamines (**118**).

Recently, Wang et al. [128] developed a new synthesis of komavine (**26**), a spiro-THβC derivative, and several analogous compounds via Pd-catalyzed cross-annulation of phenols (**170**) and tryptamine (**108**) with good functional group tolerance (Scheme 14). With this alternative protocol, THβC derivatives were synthesized with moderate to excellent yield through the C–O bond cleavage of phenols, C–H bond activation of tryptamines and C–N/C–C bond formation. The presented work offers an efficient protocol for converting phenolic lignin model monomers into valuable compounds.

**Scheme 15:**
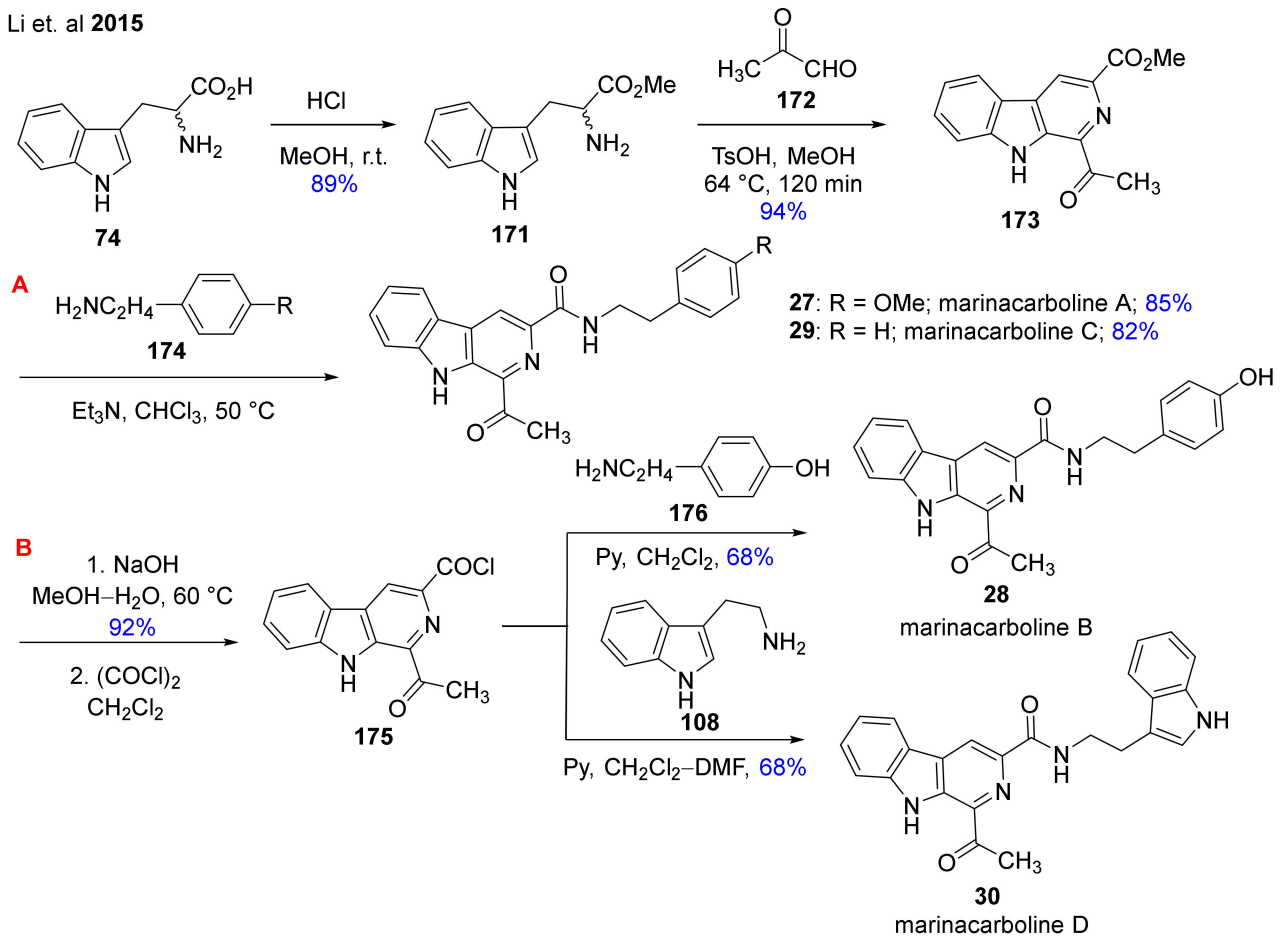
Synthesis of alkaloids marinacarboline A–D (**27**–**30**).

A series of marine β-carboline alkaloids marinacarboline A–D (**27**–**30**) was achieved by Li et al. (Scheme 15) [57]. Their strategy applies classical synthetic methods starting from racemic tryptophan (**74**). First, esterification of **74** was implemented, then a Pictet‒Spengler reaction was performed to construct the β-carboline skeleton and the key intermediate (**173**) of the synthetic route. Amidation of **173** with the appropriate amines (**174**) resulted in marinacarbolines A (**27**) and C (**29**). For the synthesis of the other two marinacarbolines (**28** and **30**), the ester group of the key intermediate **173** was hydrolyzed, then transformed into an acid chloride (**175**), which was reacted with tyramine (**176**) or tryptamine (**108**) to achieve the synthesis of marinacarboline B (**28**) and D (**30**).

**Scheme 16:**
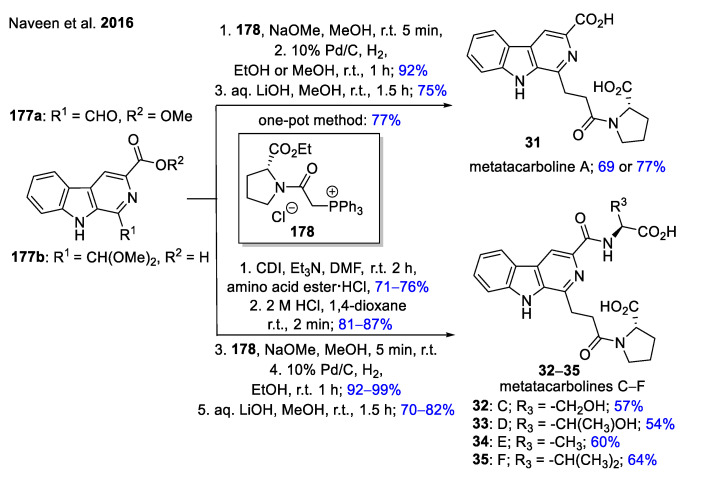
Total synthesis of metatacarbolines A, C, D, E and F (**31**–**35**).

The simple total synthesis of water-soluble metatacarbolines A (**31**) and C–F (**32**–**35**) has been accomplished starting from β-carboline building blocks **177** [59]. Metatacarboline A was prepared in 3 steps by applying the Wittig reaction of aldehyde **177a** with **178**, followed by the catalytic reduction of the previously formed double bond and hydrolysis of ester groups to afford the desired product **31** (Scheme 16). Metatacarbolines C–F were produced in 5 steps starting from the protected aldehyde precursor **177b**. First, the coupling of **177b** with various amino acid esters was performed, then a formyl moiety was formed via acidic treatment of the acetal, followed by the same three steps described previously to form the other four metatacarbolines (**32**–**35**).

**Scheme 17:**
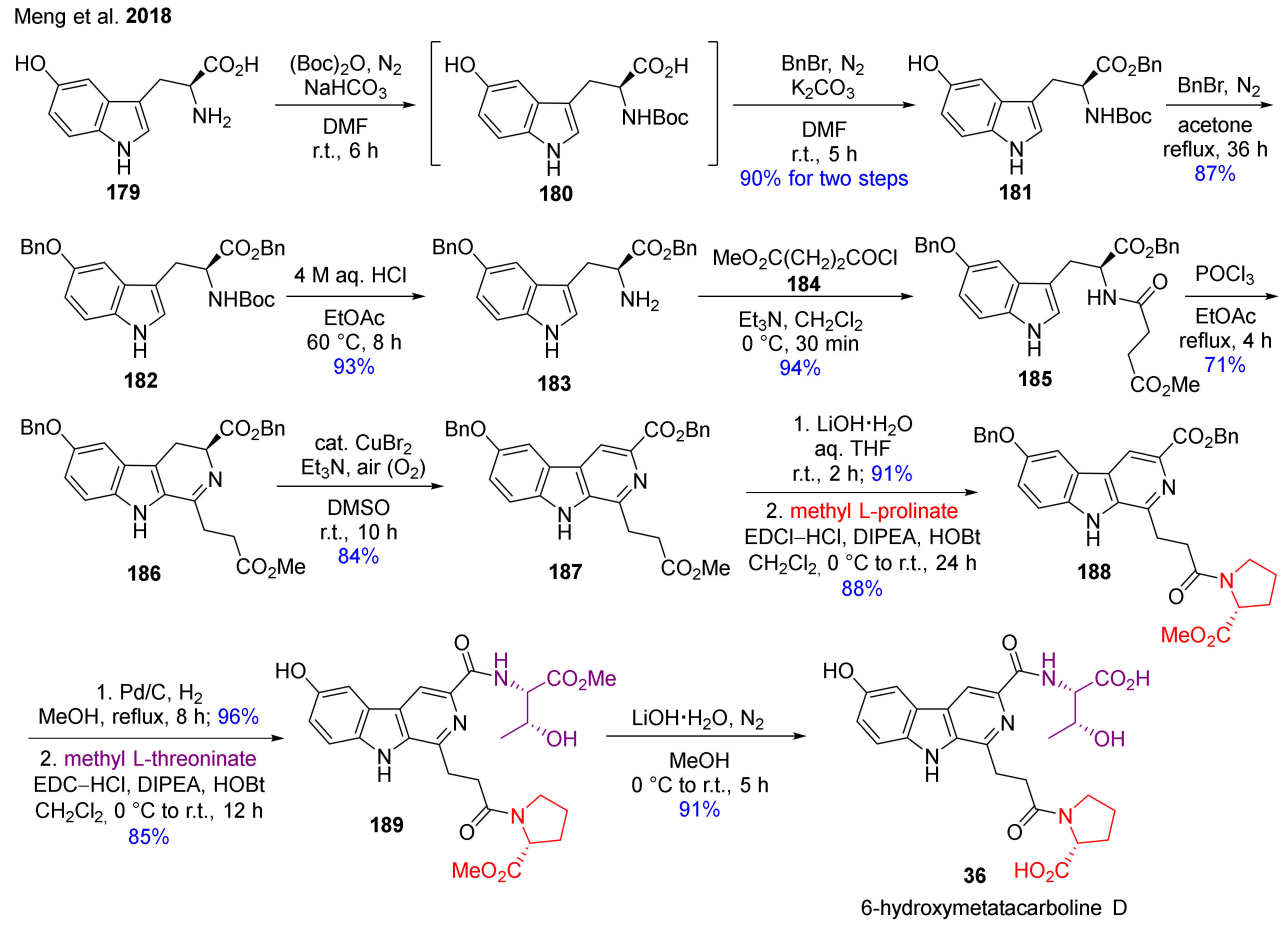
Synthesis of 6-hydroxymetatacarboline D (**36**).

The first total synthesis of 6-hydroxymetatacarboline D (**36**) has been developed in 12 steps by Meng et al. via CuBr_2_-catalyzed mild oxidation method by the dehydrogenation of the DHβC’s C-ring (Scheme 17) [129]. The synthesis starts from 5-hydroxytryptophan (**179**), which was converted into Boc- and benzyl-protected compound **182** by applying protecting group chemistry. Compound **185** was obtained through deprotection and acylation. The β-carboline skeleton of **186** was formed via Bischler–Napieralski reaction, which was followed by the mild CuBr_2_-catalyzed conversion of DHβC **186** into β-carboline **187**. The hydrolysis of ester **187** and the reaction with methyl l-prolinate followed by a catalytic debenzylation and amide formation with methyl l-threoninate led to the formation of **189**. After all, double ester hydrolysis resulted in final product **36**.

**Scheme 18:**
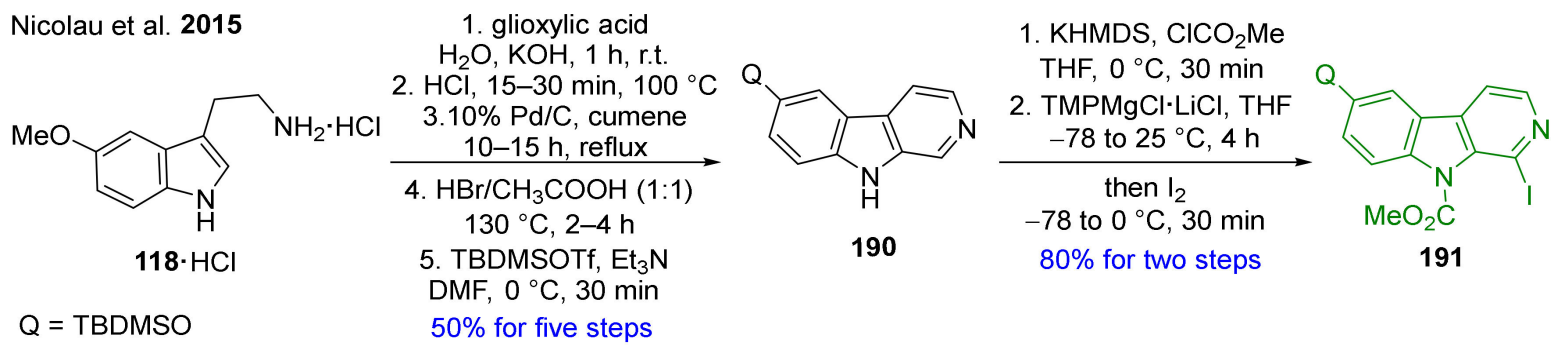
Total synthesis of shishijimicin A (**37**)—Part 1: Synthesis of β-carboline framework **191**.

**Scheme 19:**
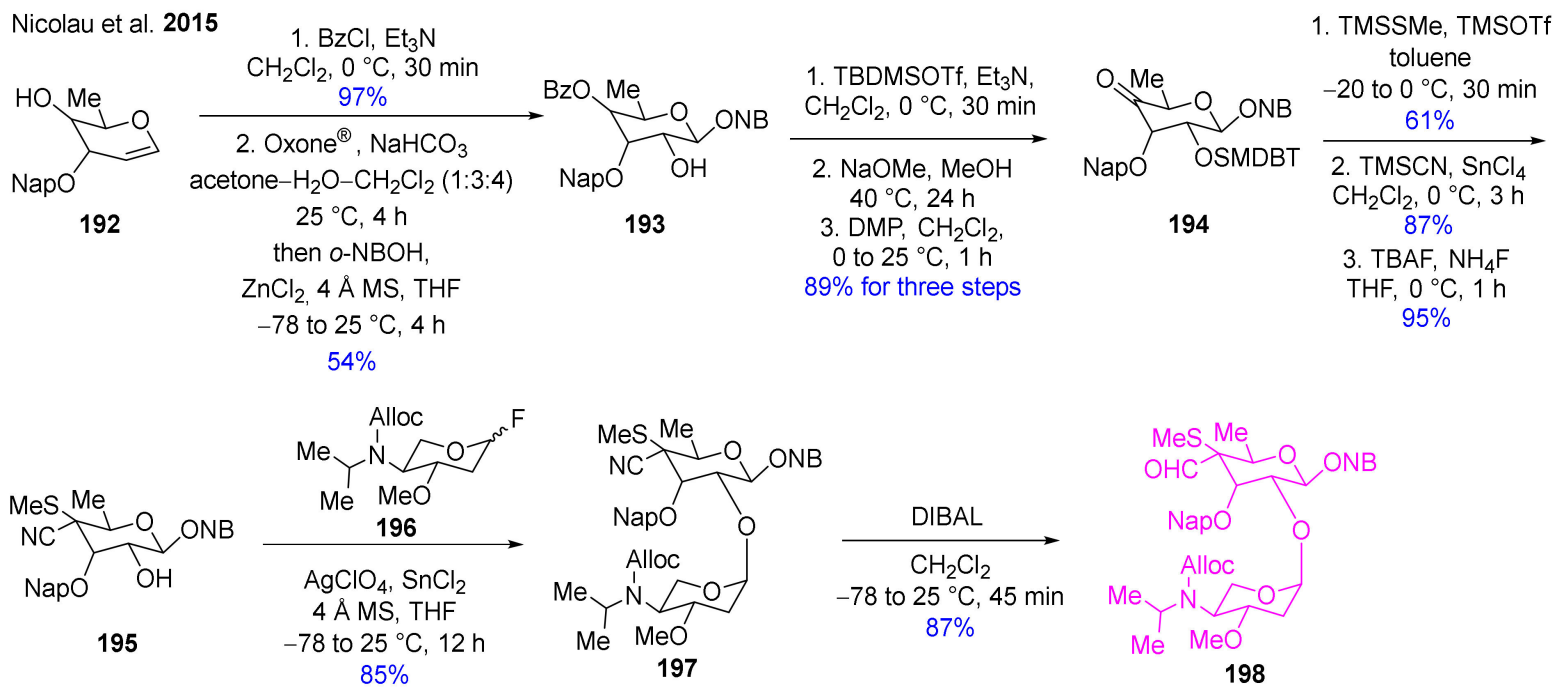
Total synthesis of shishijimicin A (**37**)—Part 2: Synthesis of disaccharide aldehyde **198**.

**Scheme 20:**
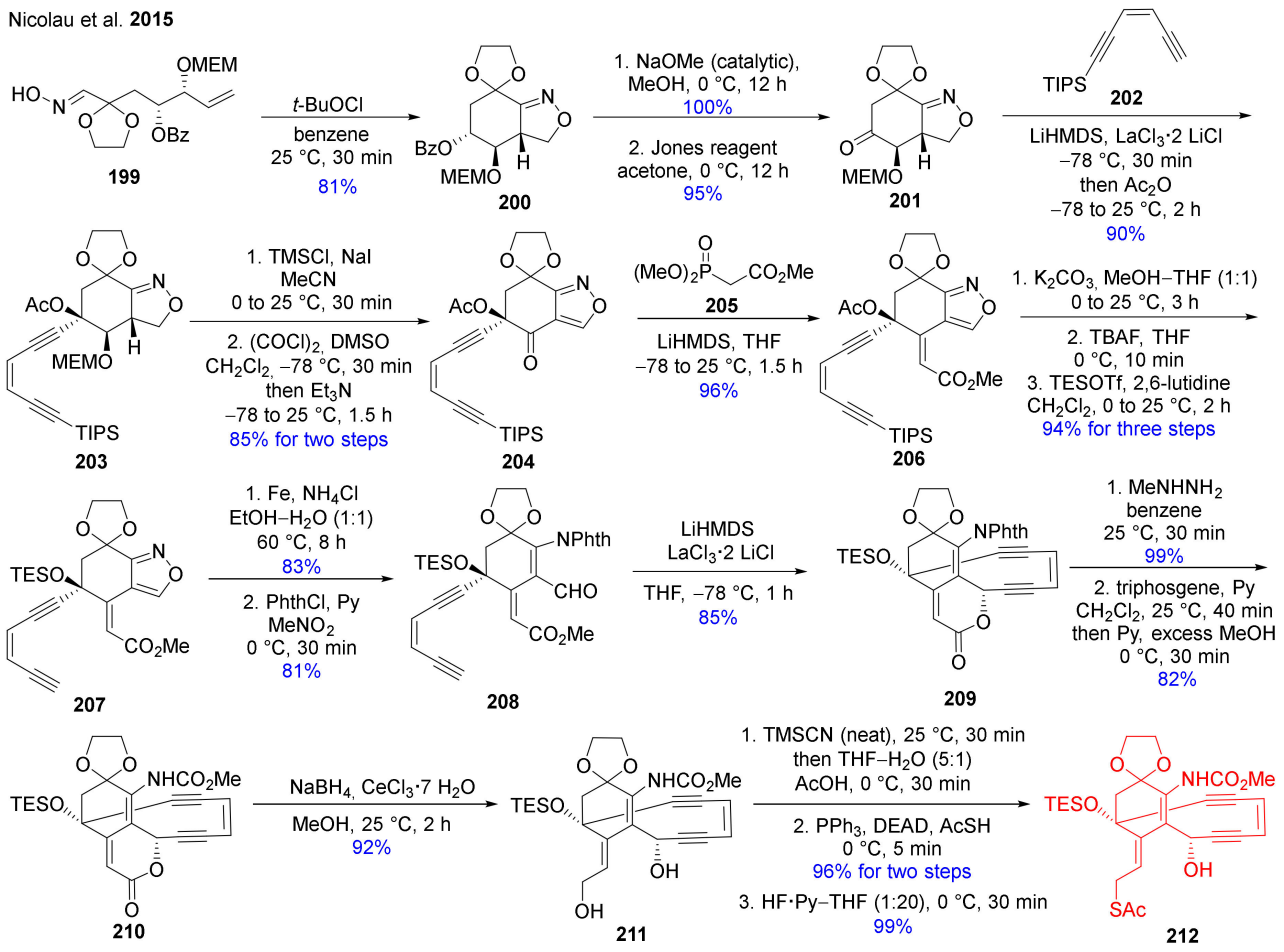
Total synthesis of shishijimicin A (**31**)—Part 3: Synthesis of enediyne thioacetate precursor **212**.

**Scheme 21:**
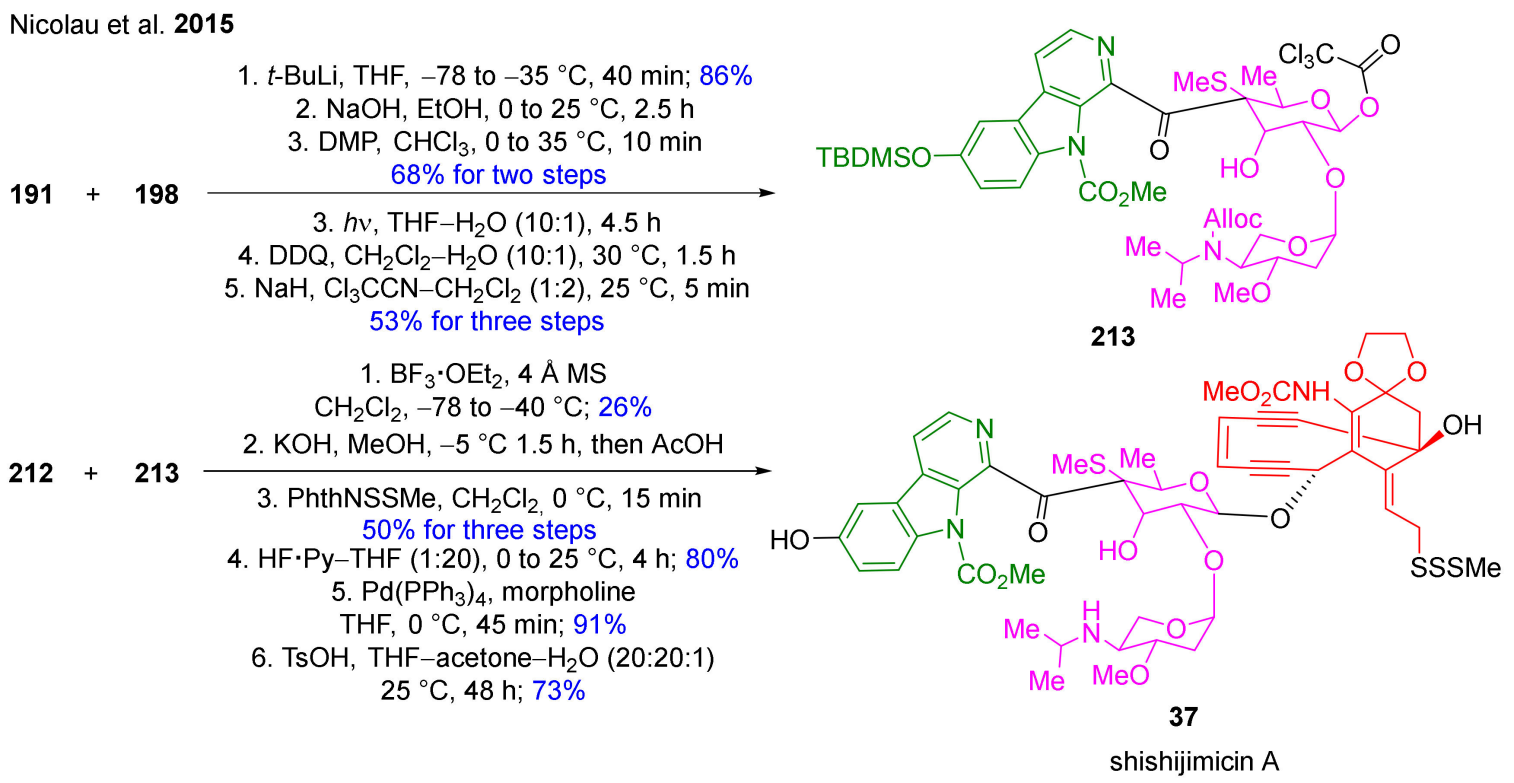
Total synthesis of shishijimicin A (**37**).

The total synthesis of shishijimicin A (**37**, Table 1), a marine natural product with remarkable antitumor activity has been described by Nicolau et al. in 48 steps (Scheme 18, Scheme 19, Scheme 20 and Scheme 21) [130]. Their method separates the natural product into three key intermediates. One of them, the β-carboline framework (**191**, Scheme 18) has been constructed in 8 steps by applying methods known from the literature [131] and functional group transformations to prepare the protected and in position 1 iodine-functionalized key intermediate (**191**).

Their convergent strategy for the construction of the complex structure of the target alkaloid **37** was continued with the synthesis of disaccharide aldehyde **198** where commercially available glucal (**192**) and glycosyl fluoride (**196**) were used as building blocks and transformed into **198** by using functional group chemistry and protecting group transformations (Scheme 19).

The synthesis of the required enediyne thioacetate precursor (**212**) started from key building block **199** proceeding in 19 steps with 25% overall yield (Scheme 20). The oxidation of oxime **199** with *t*-BuOCl resulted in a spontaneous [3+2] dipolar cycloaddition, which led to **200**. This step was followed by a smooth deprotection and oxidation, then the key enediyne fragment **202** was added to the molecule. Removal of the MEM group followed by Swern oxidation and a further oxidation step furnished ketone **204**. Horner−Wadsworth−Emmons olefination was performed, then intermediate **207** bearing a terminal acetylene bond was prepared through a sequence involving removal of the acetate group, cleavage of the TIPS moiety and silylation. Opening of the isoxazole ring was followed by the direct and stereoselective cyclization of **208** into **209** with the inversion of the configuration. The *N*-phthalide moiety was then converted to a methyl carbamate group in two steps to afford enediyne lactone **210**. Reductive cleavage of the lactone function resulted in diol **211**. Subsequent bis-silylation, selective cleavage of the primary OTMS moiety, Mitsunobu reaction, thioacetate formation and cleavage of the secondary OTMS group led to target molecule **212**.

In the final steps, the β-carboline skeleton **131** was connected to the disaccharide **198,** then various transformations were carried out to achieve intermediate **213** (Scheme 21). Finally, enediyne **212** and **213** were combined to complete the synthesis of shishijimicin A (**37**).

## 3. Fused Ring Systems Bearing a β-Carboline Skeleton

**Scheme 22:**
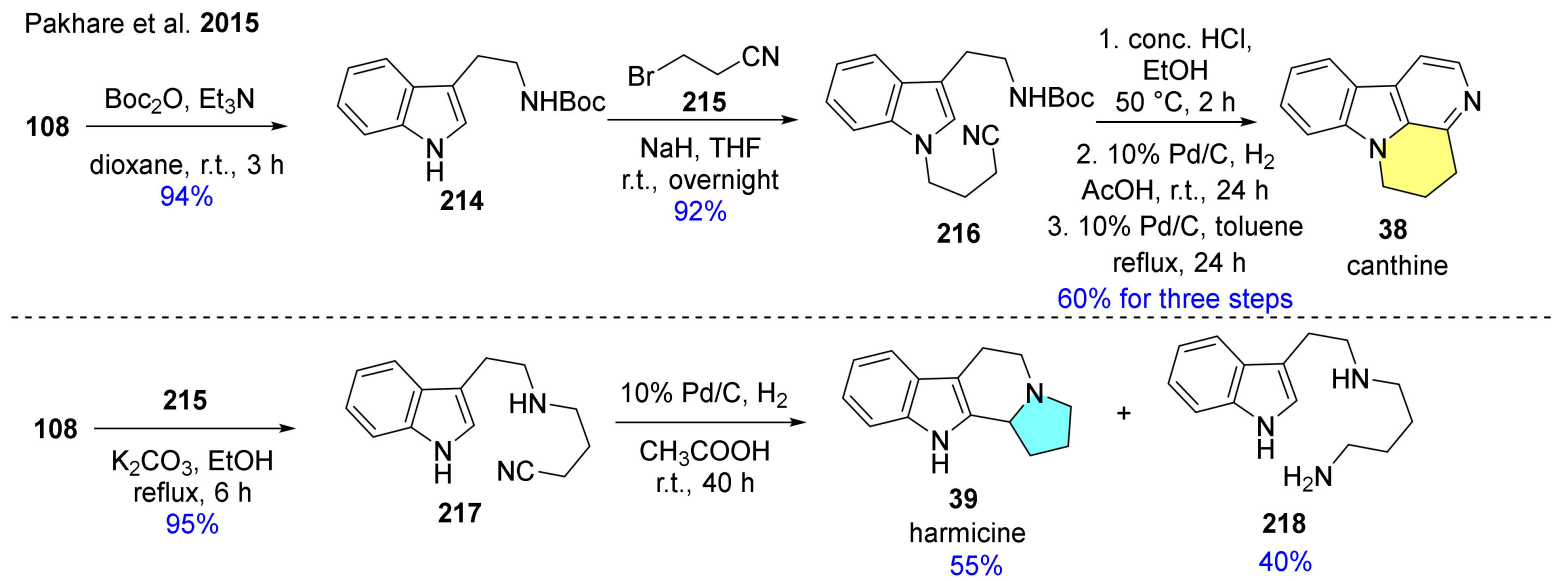
Synthesis of canthine (**32**) and harmicine (**33**) via reductive Pictet–Spengler cyclization.

The reductive Pictet–Spengler reaction has been applied successfully for the preparation of canthine (**38**), the framework of biologically active canthine-6-ones, and harmicine (**39**) (Scheme 22) [120]. Instead of the commonly used aldehydes, nitriles (**216**, **217**) were applied in the reductive intramolecular cyclization protocol, which was carried out with 10% Pd/C and H_2_. An additional dehydrogenation of the C-ring in the presence of 10% Pd/C catalyst was needed to form **38**.

**Scheme 23:**
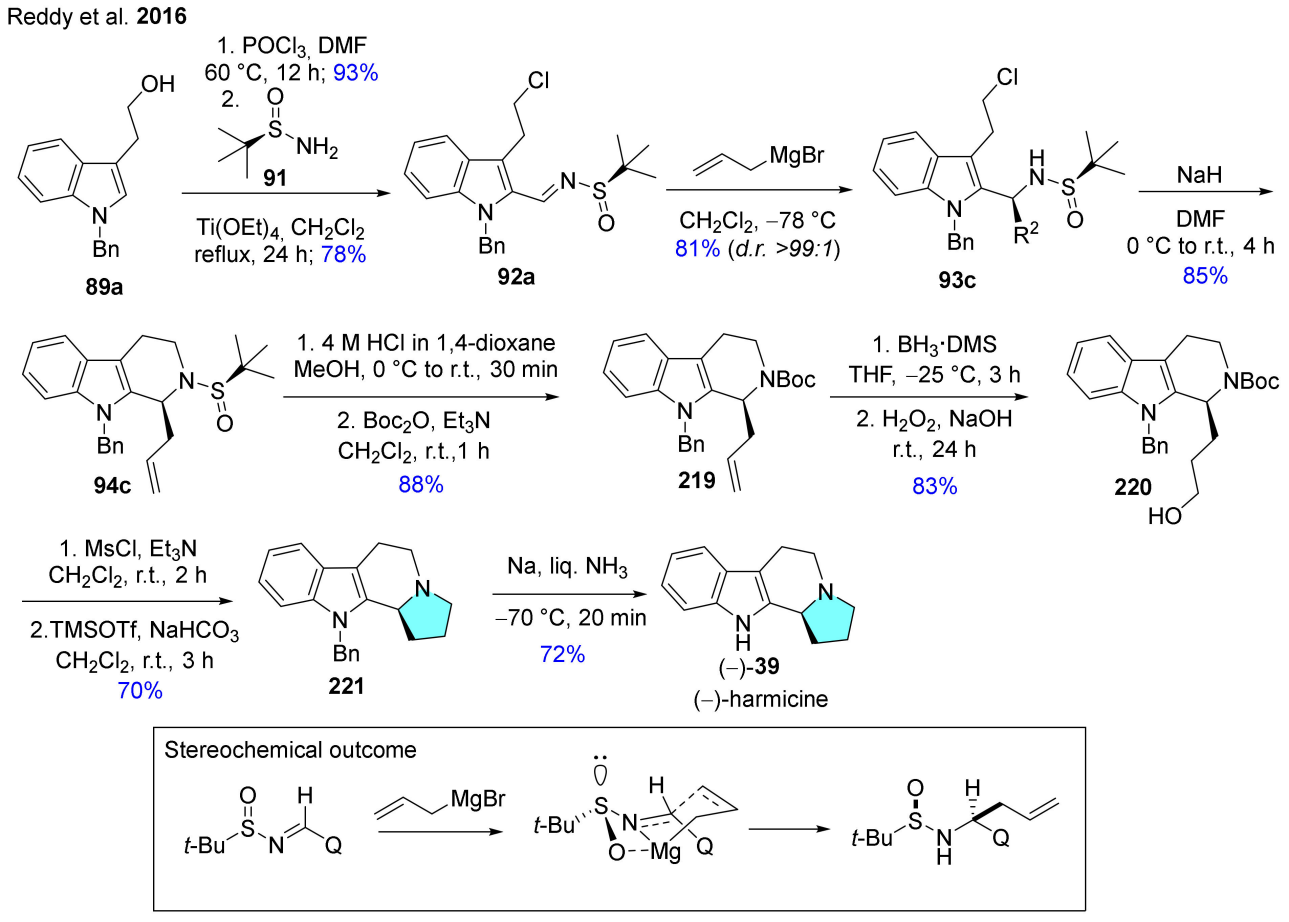
Synthesis of (−)-harmicine ((−)-**39**).

The synthetic route of (−)-harmicine ((−)-**39**) was performed as previously mentioned in part in Scheme 2, however with some differences (Scheme 23) [112]. *N*-Sulfinylimine was reacted with allylmagnesium bromide to afford the homoallylic sulfinamide with >99:1 ratio of diastereomers. In this reaction, a six-membered transition state also occurred, providing a high level of stereocontrol [132,133,134]. Compound **94c** was prepared via base-catalyzed cyclization, then deprotection of the sulfinyl group and protection of the free amine with Boc_2_O furnished **219**. Hydroboration of **219** resulted in alcohol **220**, which was converted in the next step into its mesylate. Then deprotection of the Boc group and S_N_2 substitution of mesylate with a secondary amine took place. In the final step, debenzylation of compound **221** led to (−)-**39**.

**Scheme 24:**
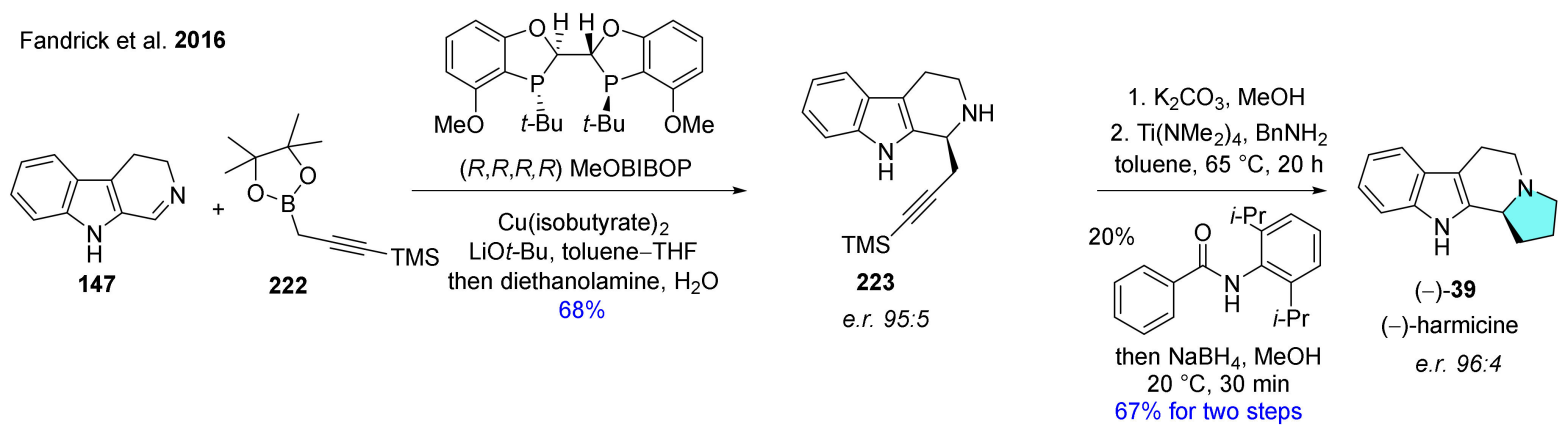
Synthesis of (−)-harmicine ((−)-**39**).

The copper-catalyzed propargylation of cyclic aldimines was developed by Fandrick et al. (Scheme 24) [135]. By applying this procedure, (−)-(*S*)-harmicine ((−)-**39**) was synthesized from DHβC (**147**), using coupling agent **222**, copper(II)isobutyrate and a chiral ligand to give intermediate **223**, which was deprotected and transformed into (−)-**39** in a titanium-catalyzed ring closure and subsequent C=N bond reduction with good yield and excellent stereoselectivity.

**Scheme 25:**
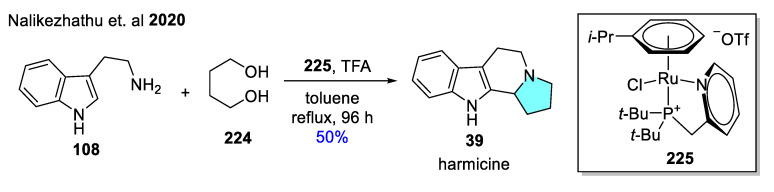
One-step synthesis of racemic harmicine (**39**).

The one-step synthesis of racemic harmicine (**39**) has been recently reported by Nalikezhathu et al. (Scheme 25) [136]. The new method is based on the pyridyl-phosphine ruthenium(II)-catalyzed tandem Pictet‒Spengler reaction through the amination of alcohols. The β-carboline skeleton was effectively constructed providing several synthetic analogues. The subsequent cycloamination was also successfully applied for the synthesis of carbolines including harmicine **39**, where its tetracyclic structure was built up in only one step from tryptamine (**108**) and 1,4-butanediol (**224**), in the presence of trifluoroacetic acid and Ru-catalyst (**225**).

**Scheme 26:**
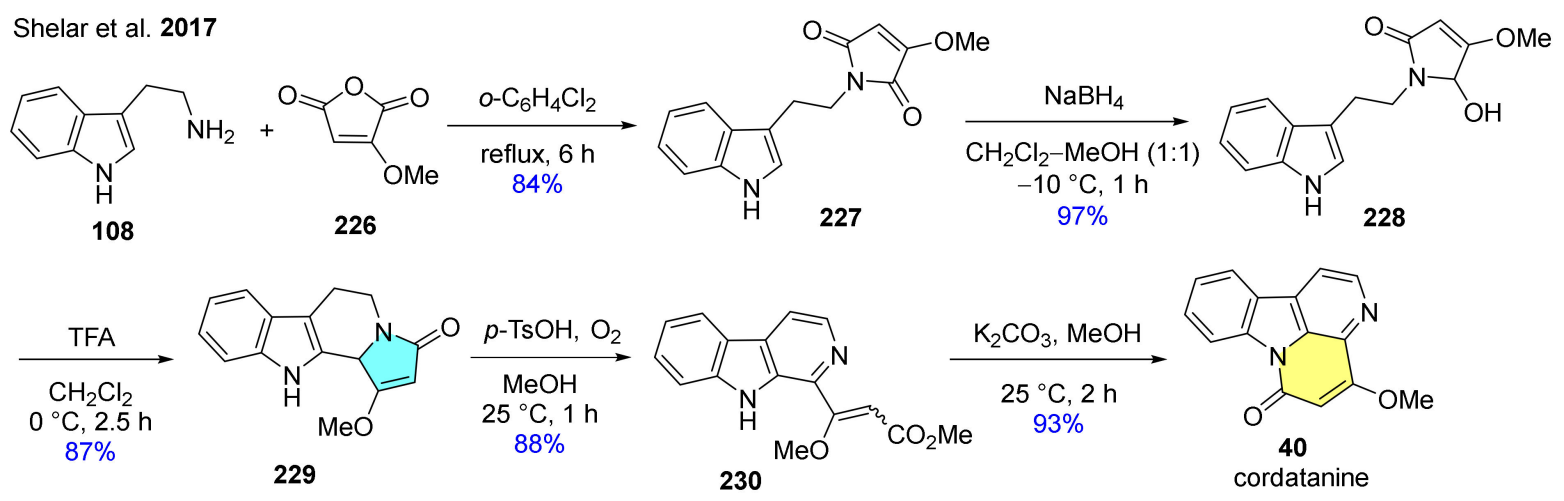
Five-step synthesis of cordatanine (**40**).

The practical total synthesis of canthine derivative cordatanine (**40**) has also been accomplished (Scheme 26) [137] starting from tryptamine (**108**) and methoxymaleic anhydride (**226**), and by subsequent regioselective reduction of methoxymaleimide intermediate **227** into hydroxy derivative **228**. Acid-catalyzed intramolecular dehydrative cyclization of **228** furnished compound **229**. The methanolysis of lactam to ester and double oxidative aromatization of the C-ring took place in a one-pot reaction to give **230**. The intramolecular cyclization in the presence of K_2_CO_3_ in MeOH led finally to cordatanine (**40**).

**Scheme 27:**
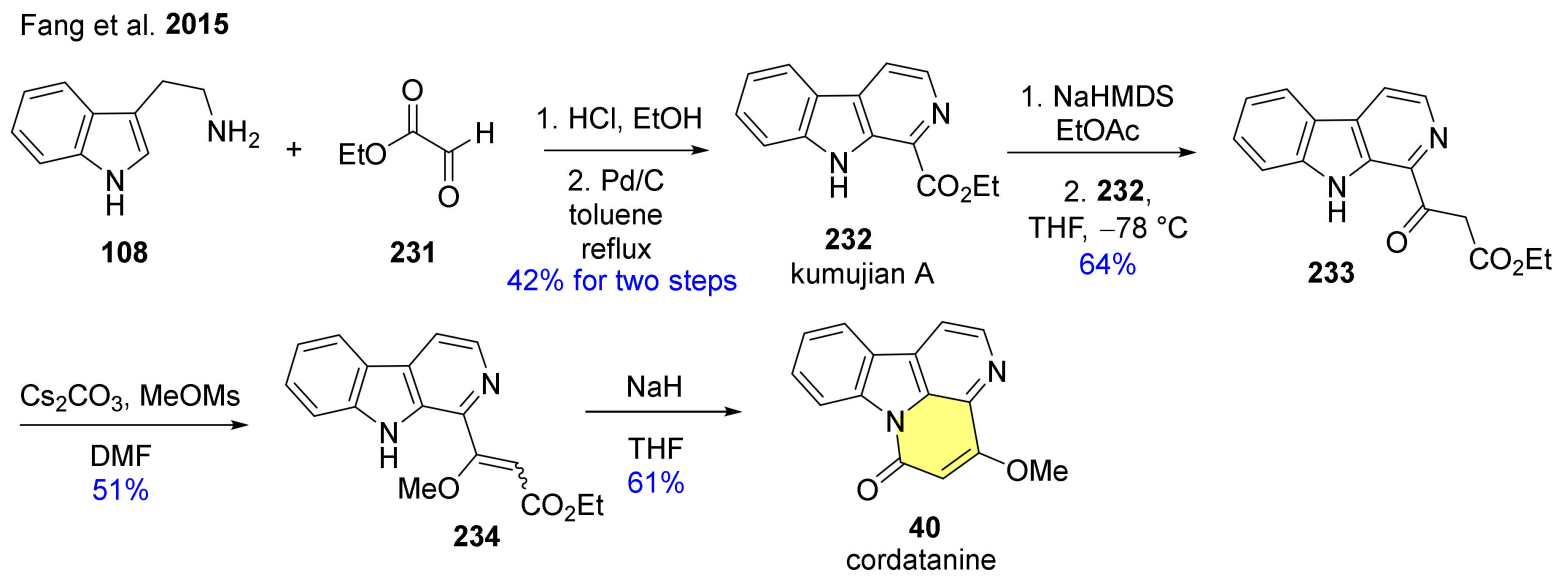
Alternate synthesis of cordatanine (**40**).

An alternate synthesis of cordatanine (**40**) has also been developed (Scheme 27) [138]. Tryptamine (**108**) was used again as the starting material which underwent a Pictet–Spengler reaction with ethyl glyoxalate (**232**), followed by direct oxidative aromatization catalyzed by Pd/C to give the synthetic intermediate kumujian A (**232**). Intermediate **232** was transformed into **233** via Claisen condensation. *O*-Methylation of **233** with methyl methanesulfonate in the presence of cesium carbonate resulted in a cis-trans mixture of **234**. The final ring closure reaction was achieved by intramolecular amidation using NaH to obtain target molecule **40**.

**Scheme 28:**
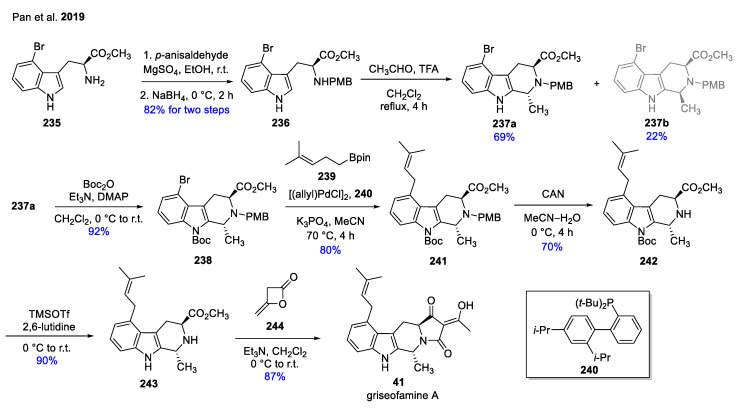
Total synthesis of griseofamine A (**41**).

The first total synthesis of natural product griseofamine A (**41**) together with its diastereomer 16-epi-griseofamine A was described by Pan et al. (Scheme 28) [67]. The synthesis commences with the introduction of PMB group to commercially available **235** via reductive amination step to prepare **236**. Then, a stereoselective Pictet–Spengler reaction was performed with the appropriate acetaldehyde for the construction of β-carboline skeleton. The reaction resulted in a mixture of diastereomers which were separated by column chromatography. The synthesis continued with the major diastereomer **237a**. First, the Boc-protection took place to afford intermediate **238**, which was further transformed with reagent **239** in a Suzuki–Miyaura cross-coupling reaction using Buchwald’s protocol with ligand **240**. The linear prenylated product **241** went through multiple deprotection protocol (from **241** to **242**). Finally, the tandem acylation/Lecay–Dieckmann condensation was performed avoiding the commonly used strong bases to achieve the first synthesis of **41**. The diastereomer of **41**, i.e., 16-epi-griseofamine A was prepared on the same reaction pathway with the slight difference that **237b** was applied instead of **237a**.

**Scheme 29:**
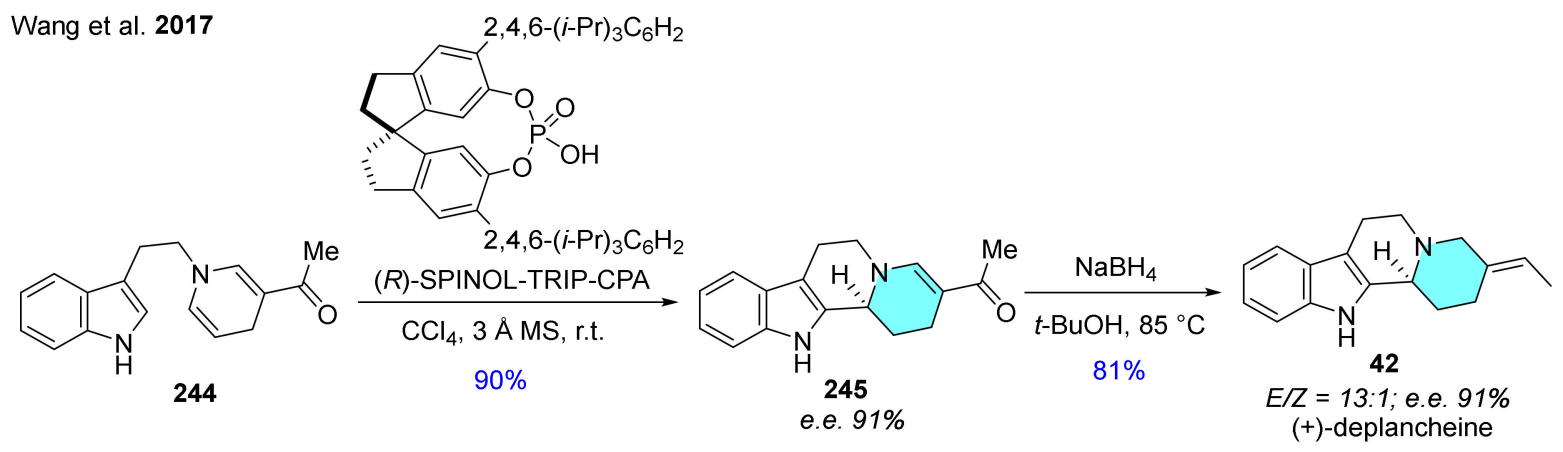
Total synthesis of (+)-deplancheine (**42**).

The construction of chiral β-carboline alkaloid (+)-deplancheine (**42**) has been achieved via (*R*)-SPINOL-TRIP-CPA-catalyzed asymmetric Pictet–Spengler reaction of indolyl dihydropyridine derivative **244** in two steps (Scheme 29) [54]. Intermediate **245** obtained with good enantiomeric excess was reduced with NaBH_4_ to obtain the desired product **42**.

**Scheme 30:**
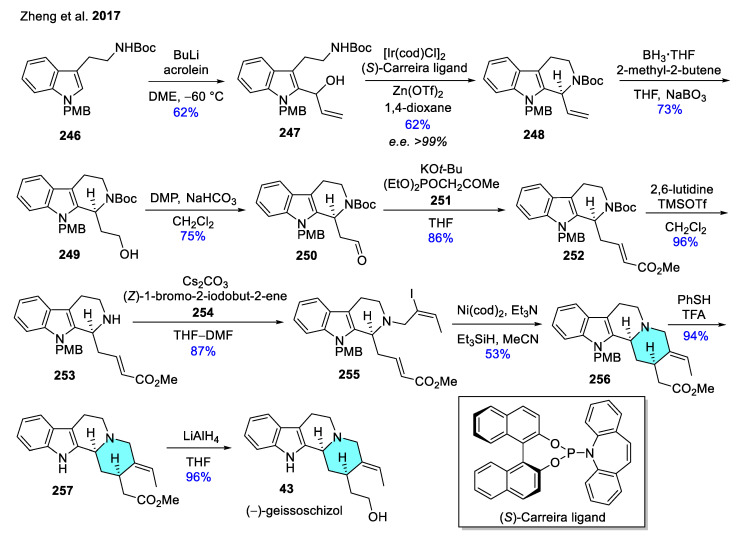
Synthesis of (−)-geissoschizol (**43**).

The enantioselective synthesis of (−)-geissoschizol (**43**) through Ir-catalyzed intramolecular ring closure as the key step has been accomplished by Zheng et al. (Scheme 30) [139]. The secondary allylic alcohol **247** was prepared from the reaction of protected tryptamine **246** and BuLi in the presence of acrolein. With key intermediate **247** in hand, the authors have attained a cyclization to forge the β-carboline skeleton. Intermediate **248** was prepared with Ir-catalyst in the presence of Zn(OTf)_2_ promoter and (*S*)-Carreira ligand in 62% yield with excellent e.e. The synthesis was continued with functional group transformations, aiming at the preparation of the corresponding aldehyde **250**. Then, a Wittig reaction was performed to give **252**, followed by deprotection and *N*-alkylation (from **253** to **255**). The formation of D-ring has been implemented via Ni-catalyzed cyclization to form intermediate **255**. Finally, deprotection of the indole nitrogen and reduction of the ester group of **257** resulted in target molecule **43**.

**Scheme 31:**
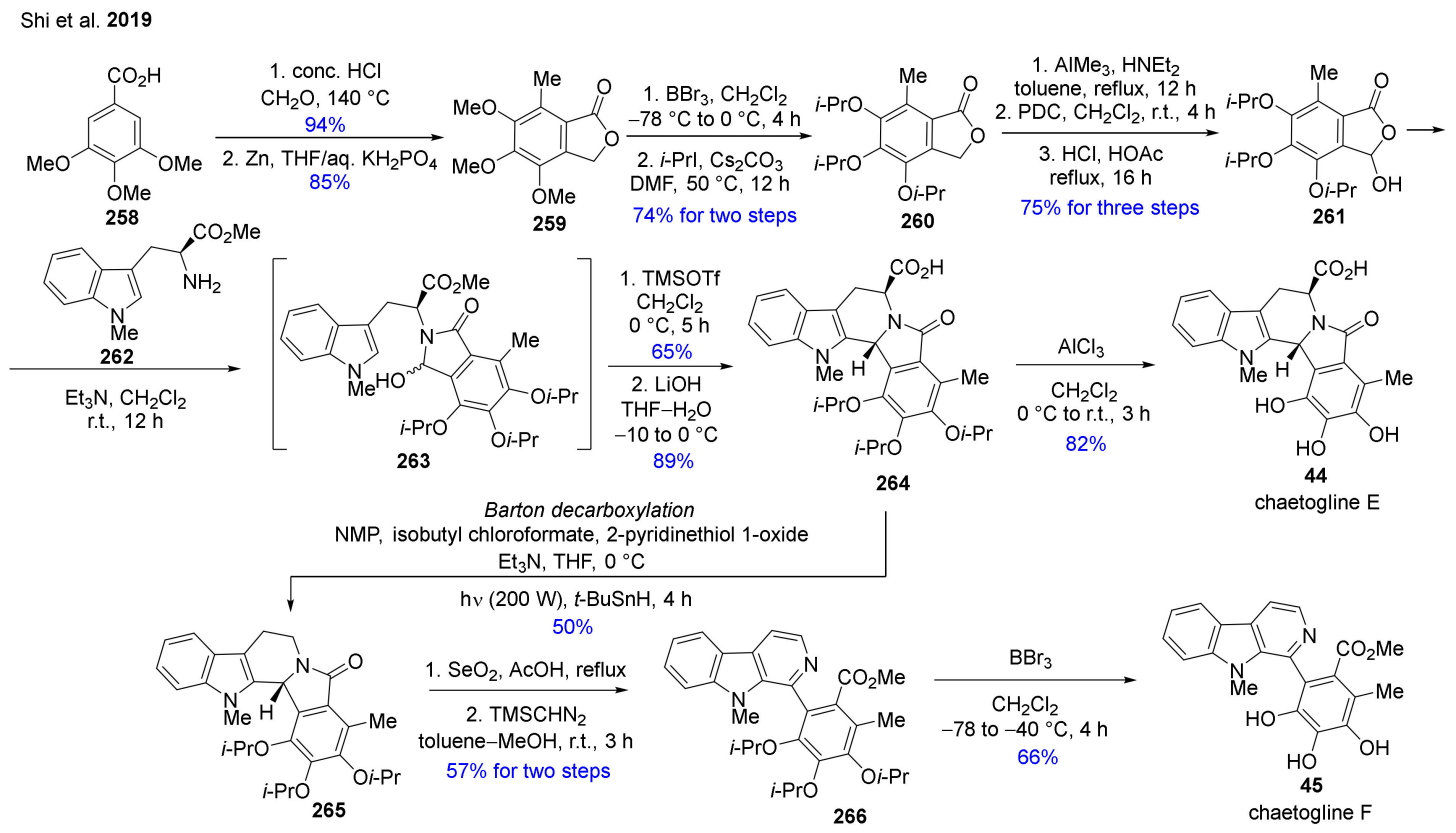
Synthesis of chaetoglines E (**44**) and F (**45**).

The silent fungal Pictet–Spenglerase gene of *Chaetomium globosum* 1C51 residing in *Epinephelus drummondhayi* guts has been described to enable Pictet–Spengler reaction between 1-methyltryptophane and flavipin to give unnatural products containing novel skeletons in a natural way. Chaetogline alkaloids are representative compounds of this gene-implied strategy as a biosynthetic machinery to produce bioactive compounds [71]. Lei et al. have recently described the first total synthesis of alkaloids chaetogline C–F, among which chaetogline E (**44**) and F (**45**) are β-carbolines, derived from the genetically modified fungus via a divergent synthesis way (Scheme 31) [140]. The synthesis began with the commercially available 3,4,5-trimethoxybenzoic acid (**258**) to produce previously described lactone **259**. After protecting group transformations, **260** was formed, which was transformed into **261** through aminolysis, oxidation and hydrolysis to achieve spontaneous cyclization. Condensation of **261** with **262** resulted the intermediate **263**, which went through a Pictet–Spengler reaction and hydrolysis to give key intermediate **264** with the desired β-carboline skeleton. Further transformation of **264** led to chaetogline E (**44**) via global deprotection with AlCl_3_. For the synthesis of caethogline F (**45**), Barton decarboxylation was used to eliminate the carboxyl group and to provide **265.** After that, **265** was oxidized, hydrolyzed and methylated to afford **266**, which was deprotected with BBr_3_ and formed the desired product **45**.

**Scheme 32:**
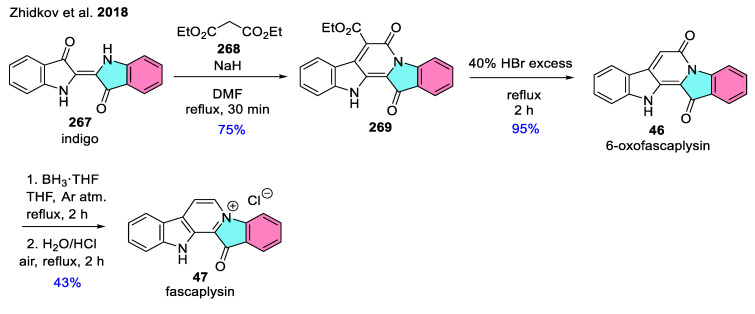
First synthesis of 6-oxofascaplysin (**46**) and a new synthetic route for the preparation of fascaplysin (**47**).

Natural products containing a 12*H*-pyrido-[1,2-a:3,4-*b*’]diindole framework like 6-oxofascaplysin (**46**) and fascaplysin (**47**) show a broad range of bioactivities. In 2018, Zhidkov et al. reported the new synthesis of **47** and the first preparation of **46** (Scheme 32) [141]. Their protocol starts with the reaction of unsubstituted indigo **267** and diethyl malonate (**268**) in the presence of NaH to give pentacycle **269**. The subsequent hydrolysis and decarboxylation were carried out under reflux conditions in an excess of 40% hydrobromic acid to furnish **46** with 70% overall yield. Reduction of the keto moiety with BH_3_·THF complex, followed by hydrolysis and oxidation with air gave **47** in 43% isolated yield.

**Scheme 33:**
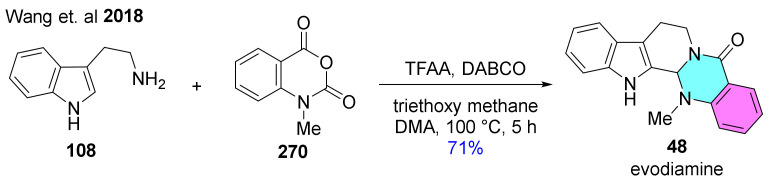
One-step synthesis of evodiamine (**48**).

The one-pot synthesis of natural product evodiamine (**48**) and a variety of its closely related derivatives was achieved using a three-component biscyclization reaction (Scheme 33) [142]. The synthesis applied readily available substrates such as tryptamine (**108**) and *N*-methylisatoic anhydride (**270**) in the presence of TFAA and DABCO. With this method, a variety of polycyclic β-carboline scaffold-containing substrates has been synthesized, however as a limitation, a few products were only obtained in poor yield.

**Scheme 34:**
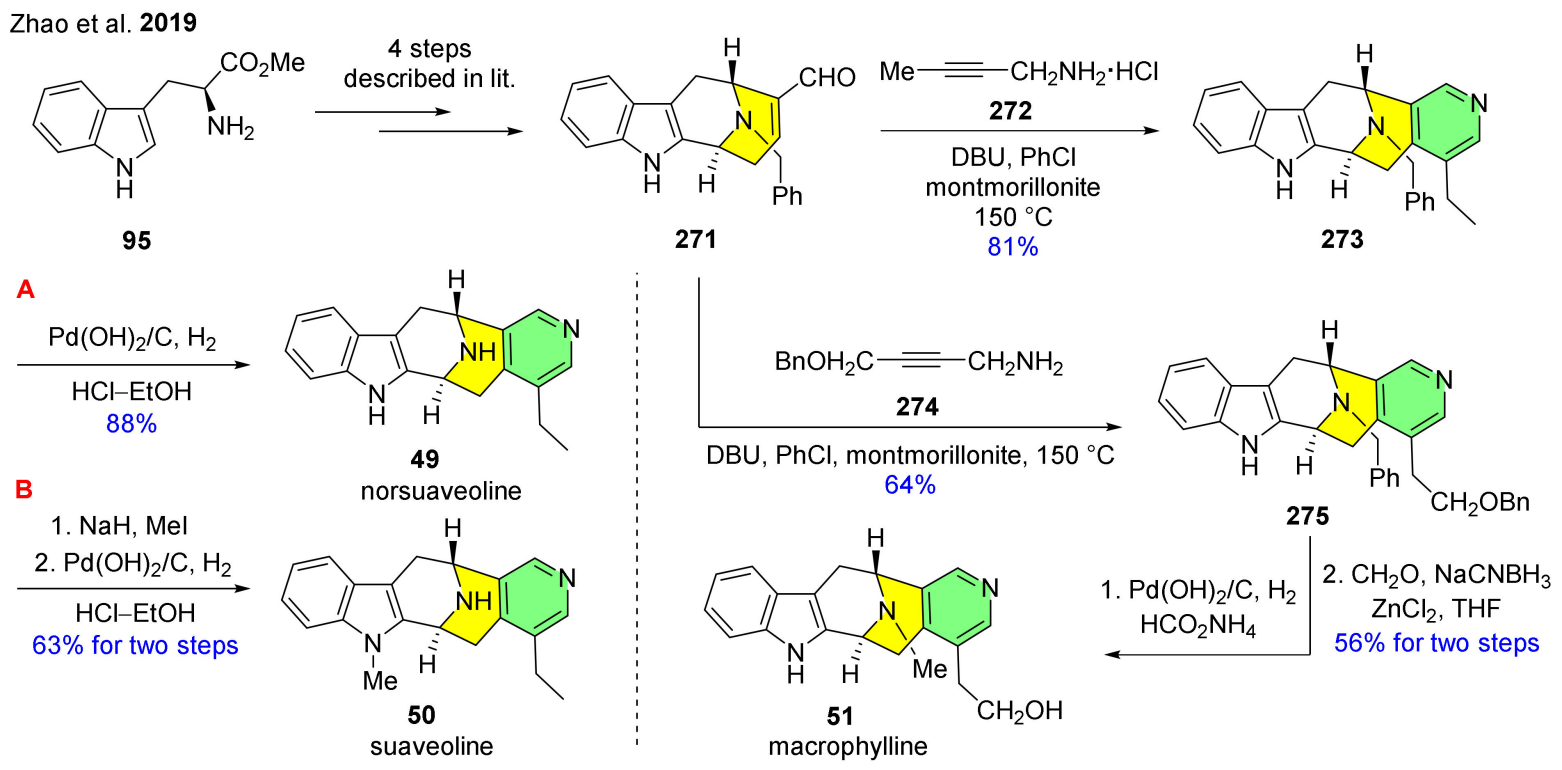
Synthesis of norsuaveoline (**49**), suaveoline (**50**) and macrophylline (**51**).

The novel synthesis of suaveoline alkaloids (**49**–**51**) was described by Zhao et al. applying a new method for pyridine incorporation starting from propargyl amines and unsaturated carbonyl compounds through a tandem reaction including condensation–alkyne isomerization–6π 3-azatriene electrocyclization sequence at the late stage of the synthesis (Scheme 34) [143]. The synthesis started from the readily available L-tryptophan methyl ester (**95**), which was converted into **271** according to previously described methods [144,145]. Next, aldehyde **271** was transformed with propargyl amine hydrochloride (**272**) into the desired pentacycle **273**, including formation of a pyridine ring. This was followed by the cleavage of the benzyl group leading to norsuaveoline (**49**). An additional methylation step was performed on indole nitrogen of **273** before *N*-debenzylation to form the other target alkaloid suaveoline (**50**). To demonstrate the flexibility of the late-stage pyridine incorporation, the authors made a reaction with **271** using another propargyl amine counterpart (**274**). The desired product **275** was further converted into macrophylline (**51**) through a Pd(OH)_2_-catalyzed debenzylation and methylation of the piperidine nitrogen atom.

**Scheme 35:**
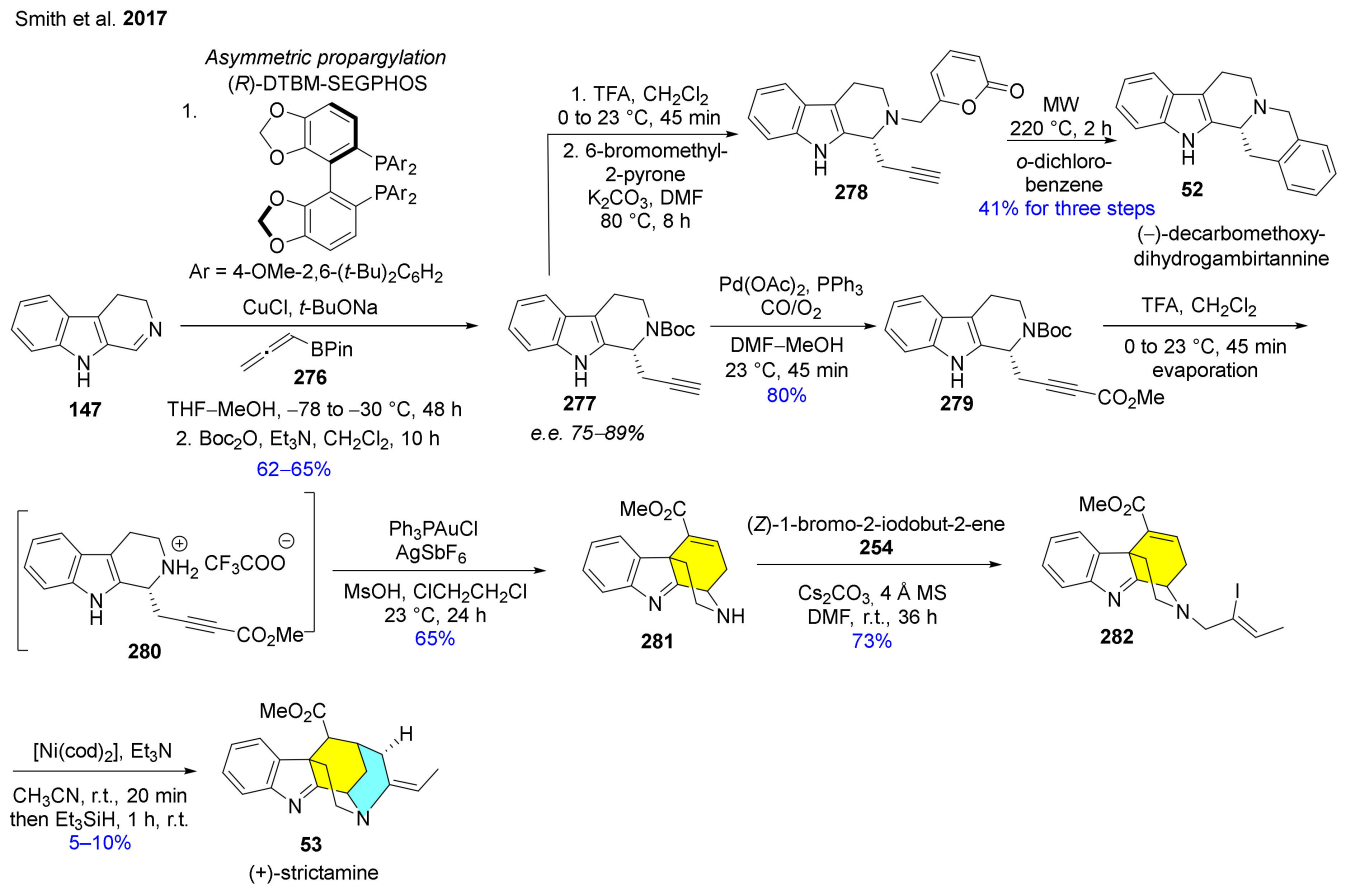
Synthesis of (*S*)-(−)-decarbomethoxy-dihydrogambirtannine (**52**) and formal synthesis of (+)-strictamine (**53**).

Smith et al. elaborated a novel asymmetric propargylation as a new approach for the preparation of β-carboline alkaloids, highlighted by the formal synthesis of (–)-decarbomethoxy-dihydrogambirtannine (**52**) and (+)-strictamine (**53**) (Scheme 35) [146]. Starting from DHβC (**147**), the propargylation was accomplished with allenylboronic acid pinacol ester (**276**) under asymmetric conditions to form **277**. To obtain **278**, **277** was reacted with TFA and 6-bromomethyl-2-pyrone. Finally, an MW-assisted cyclization was performed to complete the synthesis of target molecule **52**. For the synthesis of **53**, **277** went through further transformations and an Au(I)/Ag(I)-based 6-endo-dig cyclization to afford the core framework in compound **281**. According to previously described transformations by Zhu and co-workers [147], alkylation and Ni-promoted cyclization in the presence of Et_3_SiH led to **53** with poor yield.

**Scheme 36:**
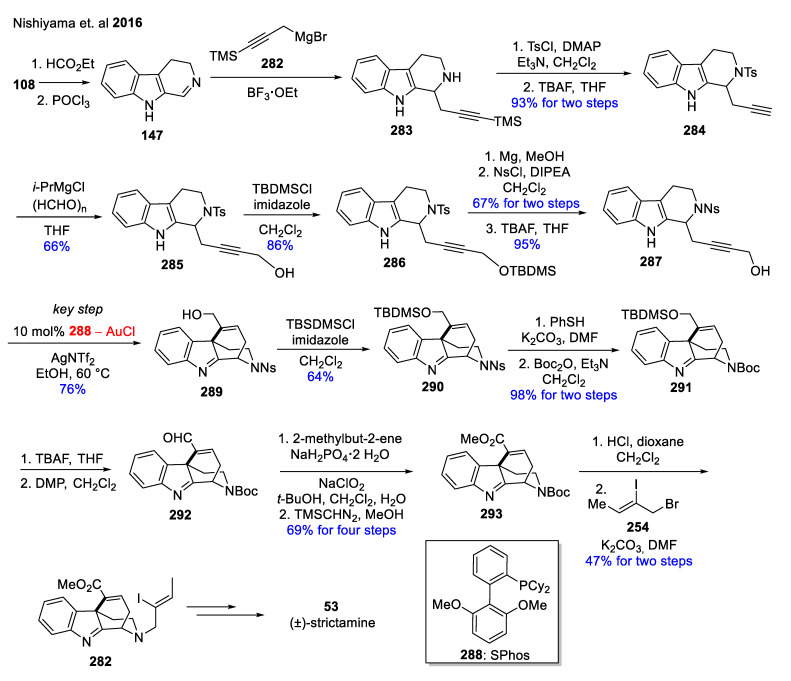
Formal total synthesis of (±)-strictamine (**53**).

Formal total synthesis of (±)-strictamine (**53**) based on gold-catalyzed cyclization was developed by a Japanese research group (Scheme 36) [148]. Starting from readily available tryptamine (**108**), a formylation with HCO_2_Et and cyclization with POCl_3_ was performed to give DHβC (**147**), followed by Grignard reactions and protecting group chemistry to afford **287** [149]. The key intermediate tetracyclic indolenine **289** was formed via SPhosAuCl (**288**)-promoted cyclization to construct the A–D ring system of akuammiline alkaloids. Functional group modifications on **289** operating with a Boc protecting group and ester group formation followed by an *N*-alkylation led to key intermediate **282.** After that, it is possible to prepare **53**, based on a previous publication [147].

**Scheme 37:**
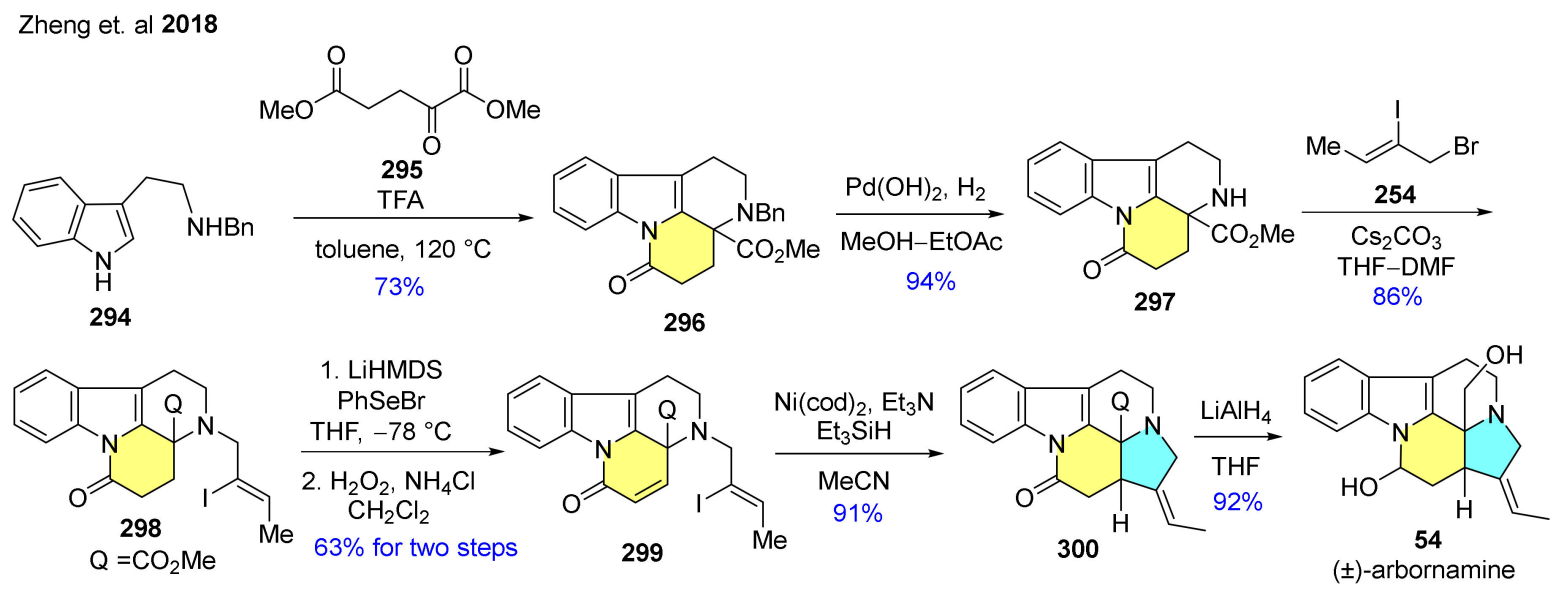
Synthesis of (±)-arbornamine (**54**).

**Scheme 38:**
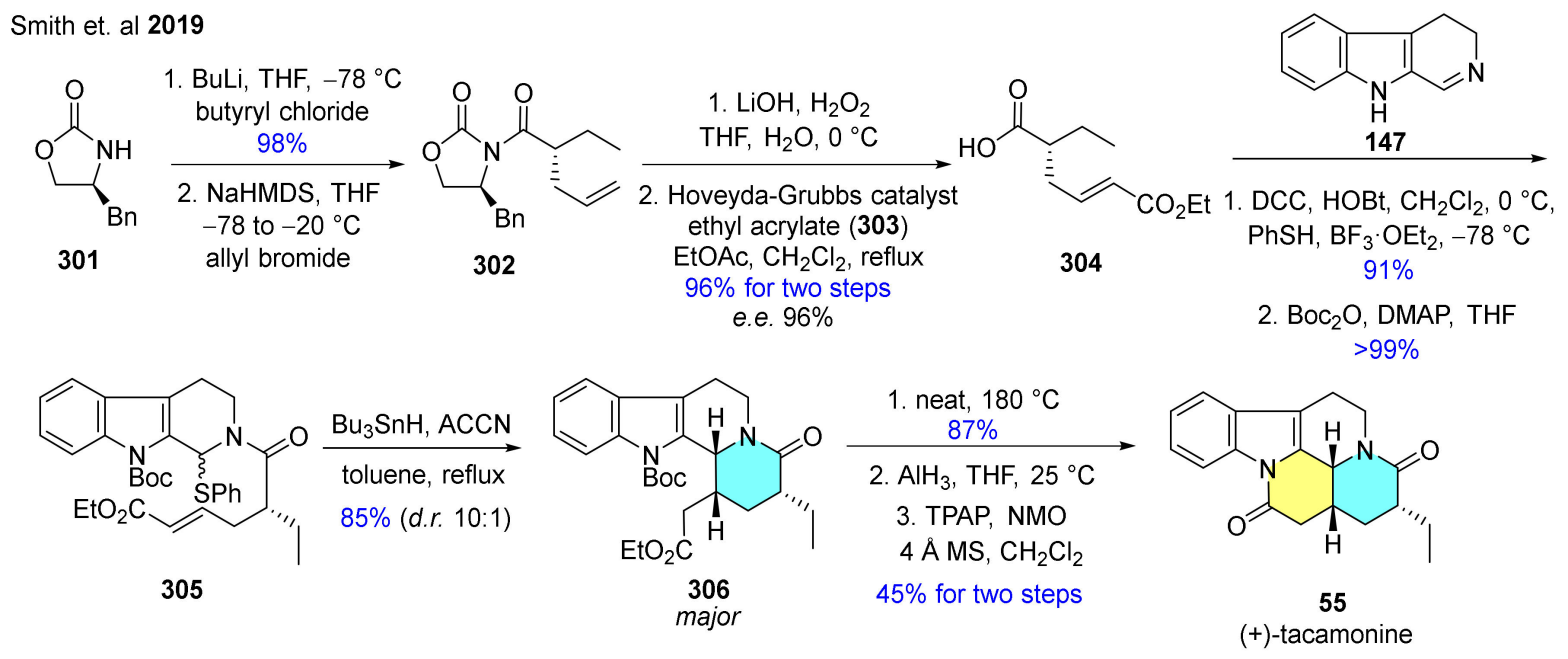
Synthesis of (+)-tacamonine (**55**).

The synthesis of monoterpene indole alkaloid (±)-arbornamine (**54**) was described in only 6 steps (Scheme 37) [150]. First, benzyl-protected tryptamine **294** was reacted with dimethyl ester **295**, resulting in intermediate **298**. Debenzylation was carried out under catalytic hydrogenation conditions to afford **297**, which was further transformed into **298** with (*Z*)-1-bromo-2-iodobut-2-ene (**254**). A general selenylation-elimination protocol resulted in the δ-lactam containing product **299**. The closure of the final ring was achieved via reductive Heck cyclization promoted by Ni(cod)_2_, leading to the appropriate pentacyclic product **300**. In the final stage, reduction of the ester moiety of **300** provided the desired alkaloid **54**.

(+)-Tacamonine (**55**), containing a pentacyclic framework, as well as its closely related bioactive derivatives like eburnamine [151] or vincamine [152] are considered as pharmacologically promising molecules. The first synthesis of (±)-tacamonine containing 3 stereocenters has been achieved by Massiot and co-workers [153]. Recently, the asymmetric synthesis of (+)-tacamonine (**55**) has been reported involving a stereoselective radical cyclization as the key step (Scheme 38) [154]. The synthesis starts from a commercially available oxazolidinone auxiliary (**301**) through an acylation followed by an allylation to form intermediate **302**. After that, **302** was modified with the removal of oxazolidinone part followed by a cross-metathesis with ethyl acrylate (**303**) resulting in chiral acid **304**. Next, intermediate **304** was reacted with DHβC (**147**) using DCC and HOBt, followed by the reaction with PhSH in the presence of BF_3_·OEt_2_ to afford α-phenylsulfanyl amide. The precursor for radical cyclization was provided by Boc-protection of the indole nitrogen atom. Then, **305** was treated with Bu_3_SnH and ACCN to form **306** which went through the thermolytic removal of the Boc group, then the reduction of the ester followed by an oxidation step with concomitant cyclization to provide **55**.

**Scheme 39:**
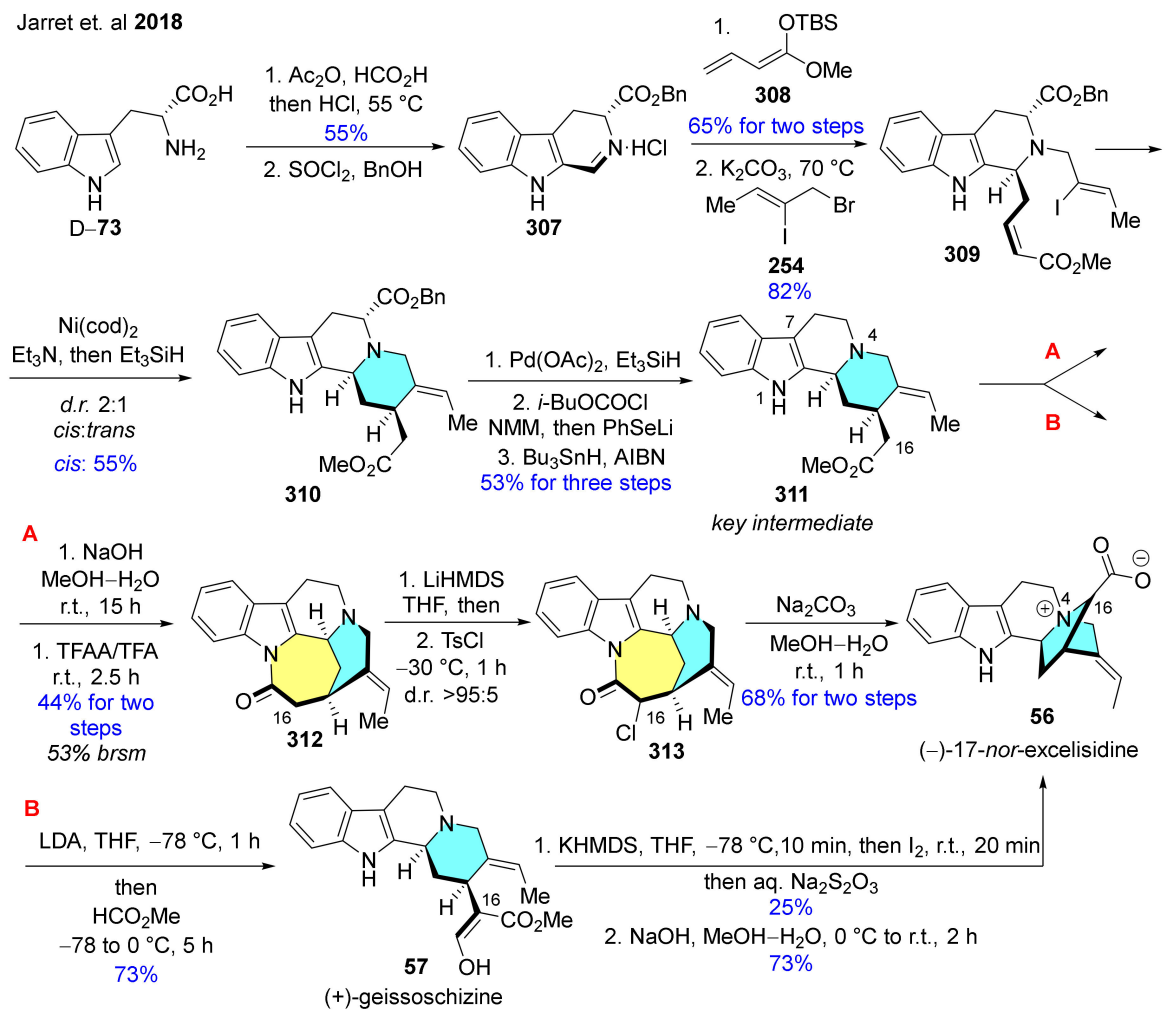
Total synthesis of (−)-17-nor-excelisidine (**56**) and (+)-geissoschizine (**57**) by applying direct oxidative cyclization.

In 2018, alkaloids containing the complete geissoschizine skeleton were synthesized via bioinspired oxidative cyclization, which led to the selective formation of the N4-C16 bond [155]. In this research, (−)-17-nor-excelsinidine (**56**) was synthesized (Scheme 39), including an α-electrophilic chlorination of a 7-membered-ring lactam and its ring rearrangement via nucleophilic substitution. Two oxidative cyclization strategies were developed to construct (−)-17-nor-excelsinidine starting from enantiopure d-trypthophan (**D–73**), which was transformed into **307** via Pictet–Spengler reaction followed by a protecting maneuver. After this, Mannich addition with **308** and *N*-alkylation with 1-bromo-2-iodobut-2-ene (**254**) were accomplished to give **309**. In the presence of Ni(cod)_2_, radical cyclization of **309** occurred, then removal of the benzyl ester resulted in key intermediate **311** containing the required scaffold. Next, the oxidative cyclization took place which was achieved on two alternative pathways (Scheme 39A,B) to produce alkaloid **56**. Following the reaction pathway A, the indole nitrogen and the carbonyl of the ester of **311** were connected by forming 7-membered-lactam **312**. The oxidative process was accomplished in two stages. First, the C16-carbon was subjected to umpolung by diastereoselective chlorination to obtain **313**. This was followed by the addition of sodium carbonate in aqueous methanol was induced the nucleophilic substitution of the chlorine by the N4-nitrogen and the cleavage of the lactam to form enantiopure (−)-**56**. Choosing another synthetic route B, **311** was transformed into (+)-geissoschizine (**57**) through a formylation step. After that, **57** was deprotonated with KHMDS and the resulted dianion was then submitted to oxidation with iodine leading to (−)-**56** methyl ester, which was then saponified to **56**.

**Scheme 40:**
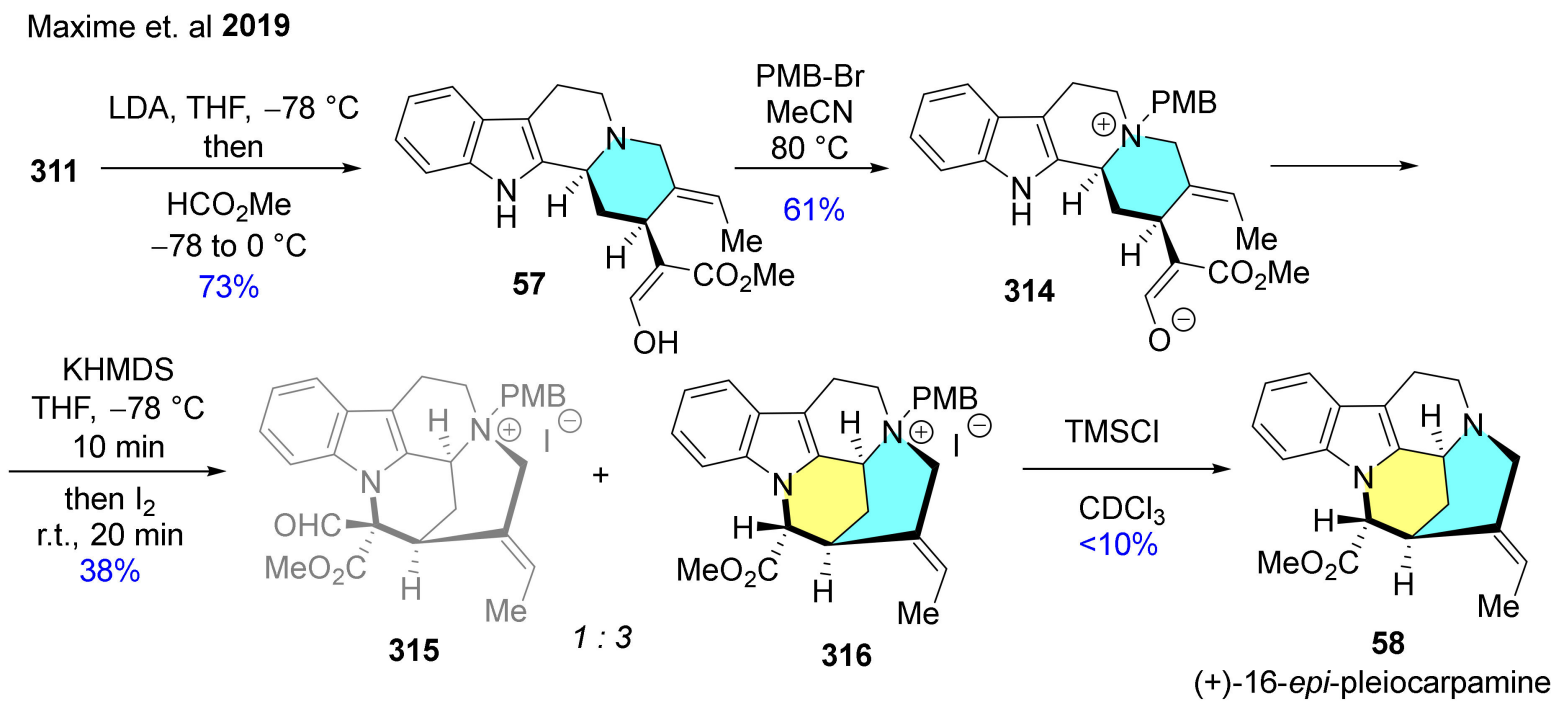
Synthesis of (+)-16-epi-pleiocarpamine (**58**).

**Scheme 41:**
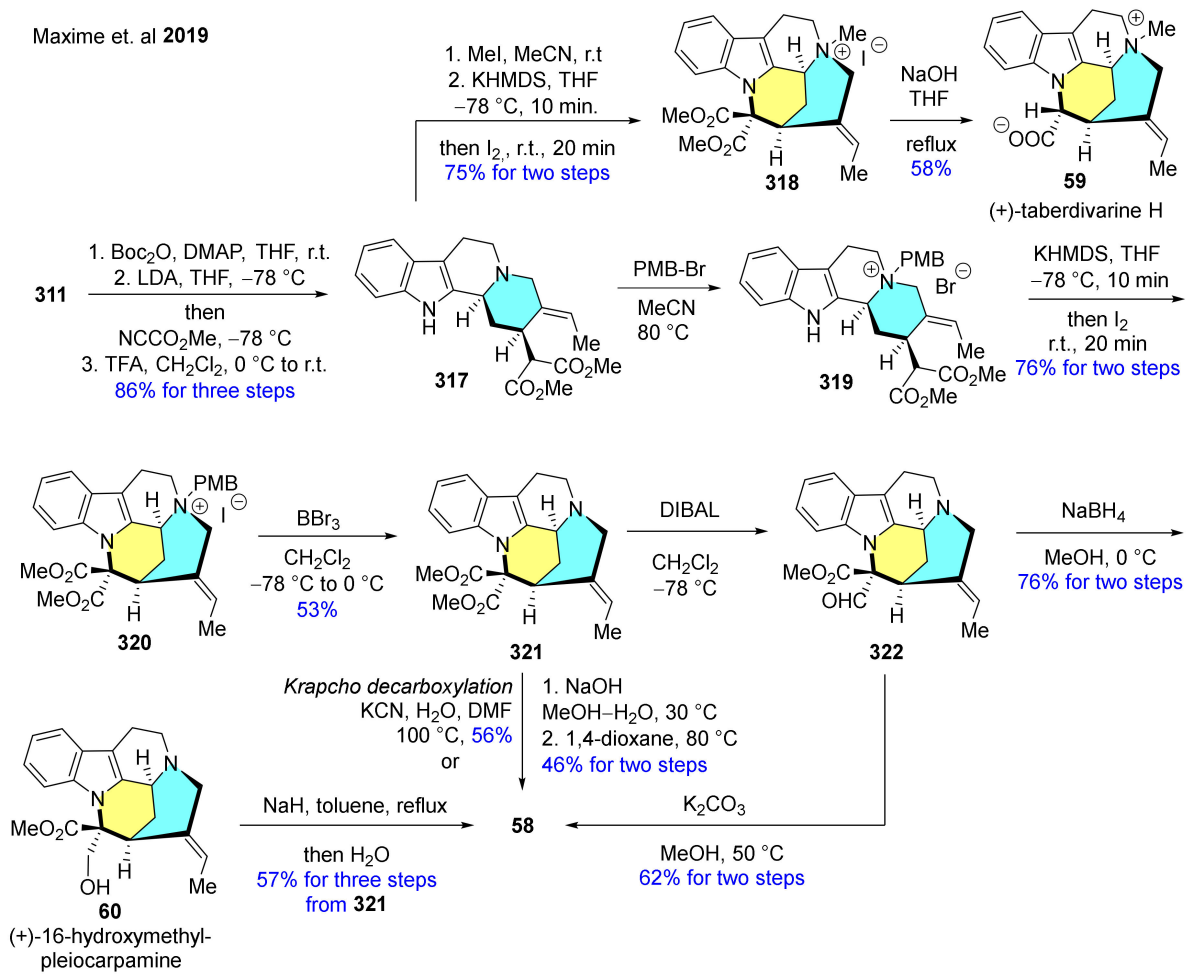
Synthesis of (+)-16-epi-pleiocarpamine (**58**), (+)-taberdivarine H (**59**) and (+)-16-hydroxymethyl-pleiocarpamine (**60**).

The same authors reported the total synthesis of further 3 closely related mavacuran alkaloids: (+)-16-epi-pleiocarpamine (**58**), (+)-taberdivarine H (**59**) and (+)-16-hydroxymethyl-pleiocarpamine (**60**) by applying their method described above for the construction of geissoschizine skeleton (Scheme 40 and Scheme 41) [156]. The synthesis of **58** starts with the formylation step of **311** leading to the formation of **57**. Heating of **57** with PMB-Br in MeCN resulted in quaternary ammonium compound **314**. In the following reaction step, deprotonation of the indole NH of **314** followed by the addition of I_2_ gave a mixture of **315** and **316** containing the desired mavacuran skeleton. Finally, dequaternization of **316** with TMSCl furnished the target molecule **58**, although with a poor yield (Scheme 40).

Similarly to the method described above, the basic framework of the target molecules was built by applying the key oxidative coupling induced by KHMDS in the presence of I_2_ as this latter masks the nucleophilicity of the aliphatic nitrogen center and locks it in the suitable *cis* configuration leading to intermediates **318** and **319** (Scheme 41). (+)-Taberdivarine H (**59**) was synthesized by quaternization of the tertiary nitrogen atom of **317** with MeI followed by an oxidative coupling to give **318**, and subsequent ester hydrolysis and selective decarboxylation. The synthesis continued with the oxidative coupling of **319** and deprotection of **320** with BBr_3_. Selective Krapcho decarboxylation of diester **321** occurred to form compound **58**. Selective reduction of key intermediate **321** with DIBAL resulted in the desymmetrized product **322**, followed by a deformylation step in the presence of NaBH_4_ to accomplish the synthesis of **60**. Compound **60** was transformed into **58** with NaH in boiling toluene and by subsequent addition of water. 

**Scheme 42:**
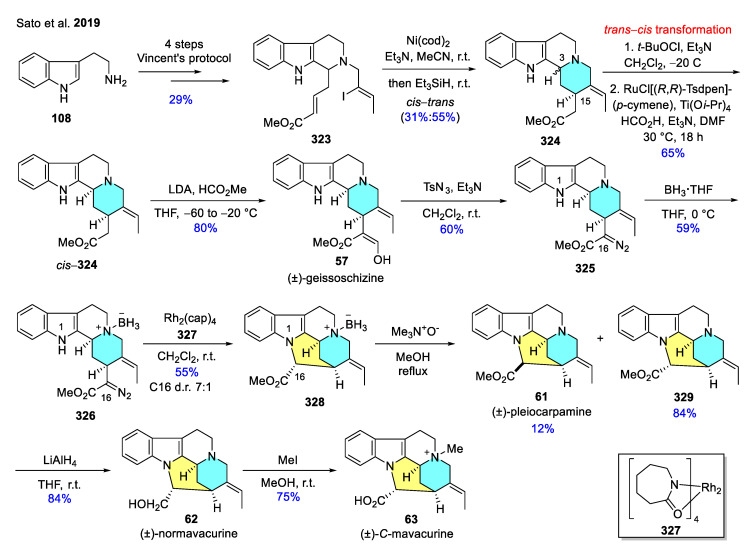
Total synthesis of C-mavacurine-type alkaloids (**61**–**63**).

The total synthesis of C-mavacurine alkaloids (±)-pleiocarpamine (**61**), (±)-normavacurine (**62**) and (±)-*C*-mavacurine (**63**) was accomplished by Sato et al. (Scheme 42) [157]. The innovative key step of the synthesis is a metal carbenoid cyclization by an N–H insertion within a Corynanthe-type compound bearing a diazo function, where the molecular conformation is fixed by an amine-borane complex. The synthesis starts with the preparation of α,β-unsaturated ester **323** from tryptamine (**108**), applying the previously reported protocol by Vincent and co-workers [155]. This was followed by the Ni(cod)_2_-mediated reductive cyclization to afford a mixture of *trans*-**324** and the desired *cis*-**324**. After that, *trans*-**324** was treated with *t*-BuOCl, then with HCl in MeOH to form an iminium intermediate, which was effectively transformed into *cis*-**324** in the presence of Noyori-type catalyst, formic acid and Et_3_N in DMF solvent. Next, *cis*-**324** was treated with LDA and methyl formate to obtain the D-ring containing (±)-geissoschizine (**57**). Natural product **57** was converted into the key intermediate of the reaction, diazo compound **325** using tosyl azide in the presence of Et_3_N. As an essential compound for the further steps, **325** was transformed into borane-complex **326** with BH_3_·THF to fix the appropriate conformation for the next steps. The cyclization precursor **326** underwent a carbene-generated cyclization with catalyst **327** to form the E-ring containing derivative **328**. The amine-borane bond was easily cleaved by heating in the presence of trimethylamine oxide to obtain (±)-pleiocarpamine (**61**) and intermediate **329**. For the synthesis of (±)-normavacurine (**62**), **329** was used in a reduction step carried out with LiAlH_4_ andfurther transformation into (±)-*C*-mavacurine (**63**) by treatment with an excess of MeI.

**Scheme 43:**
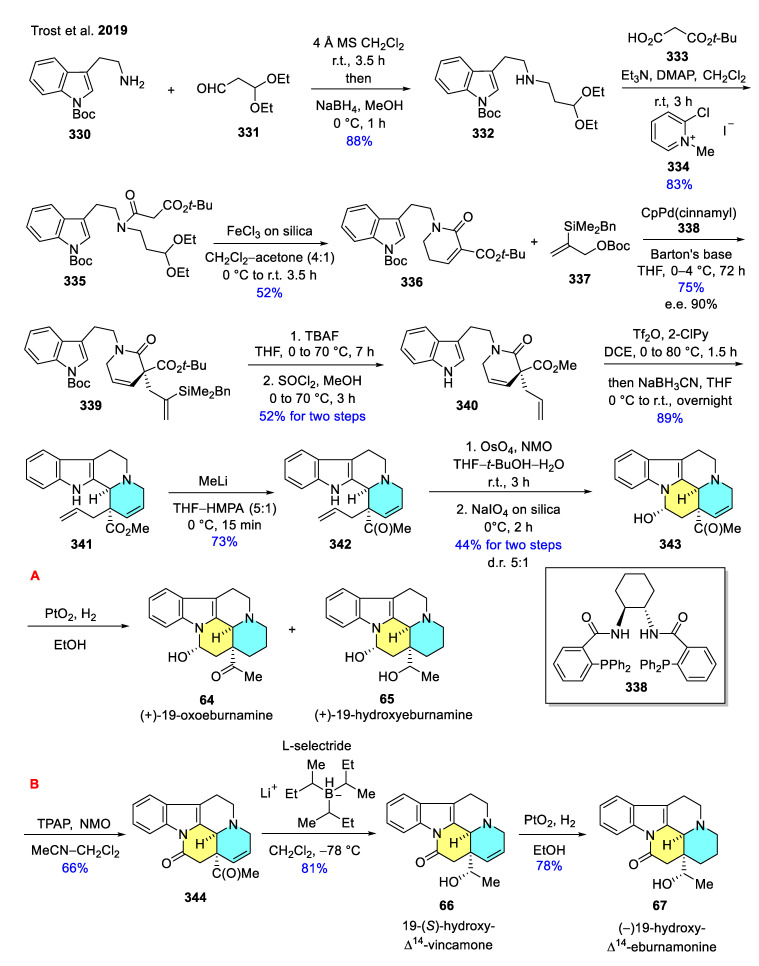
Synthesis of eburnane alkaloids (**64**–**67**).

The C19-oxo-functionalized eburnane alkaloids (**64**–**67**) were synthesized by Trost et al. (Scheme 43) [158]. The synthesis involves a Pd-catalyzed asymmetric allylic alkylation step of an *N*-alkyl-α,β-unsaturated lactam as a key step for the further ring system construction. Using Corey and Baran’s method [159], Boc-protected tryptamine (**330**) and aldehyde **331** were transformed into tryptamine derivative **332**. After that, amine **332** was reacted with *tert*-butyl malonate (**333**) in the presence of Mukaiyama’s reagent (**334**) to perform an amidation reaction. The formed intermediate **335** was treated with FeCl_3_ on silica via ketal cleavage and cyclization to provide the α,β-unsaturated lactam **336**. The reaction of intermediate **336** with nucleophile **337**, i.e., the key step of the synthesis was accomplished applying CpPd(cinnamoyl) as a catalyst, Trost ligand (**338**) and Barton’s base as an additive, giving 75% yield with e.e., 90%. The key intermediate **339** thus obtained underwent a desilylation in the presence of TBAF, followed by a simultaneous Boc-group removal and transesterification to afford **340**. In the next step, the formation of the C-ring was accomplished through a Bischler–Napieralski reaction using Movassaghi’s protocol to prepare **341** [160]. The exposure of **341** to excess of MeLi led to the corresponding ketone **342**, where no by-product was observed because of the intramolecular chelate formation. This was followed by the chemoselective dihydroxylation of terminal olefin bond in the presence of OsO_4_ and NMO, then by the reaction with NaIO_4_ on silica leading to an aldehyde intermediate, which directly cyclized to give pentacycle **343**. From this point, the synthesis was continued via divergent routes to the target molecules. First, hydrogenation took place, where the saturation of the double bond of the tetrahydropyridine ring and concomitant partial reduction of the C19 carbonyl group resulted in a mixture of (+)-19-oxoeburnamine (**64**) and 19-hydroxyeburnamine (**65**). Focusing on the other two target molecules, a Ley–Griffith oxidation was performed on **343**, which led to compound **344**. The selective reduction of C19-ketone **344** was performed in the presence of l-selectride to obtain 19-(*S*)-hydroxy-Δ^14^-vincamone (**66**). Finally, hydrogenation of **66** using 10% PtO_2_ resulted in (−)-19-hydroxy-Δ^14^-eburnamonine (**67**).

**Scheme 44:**
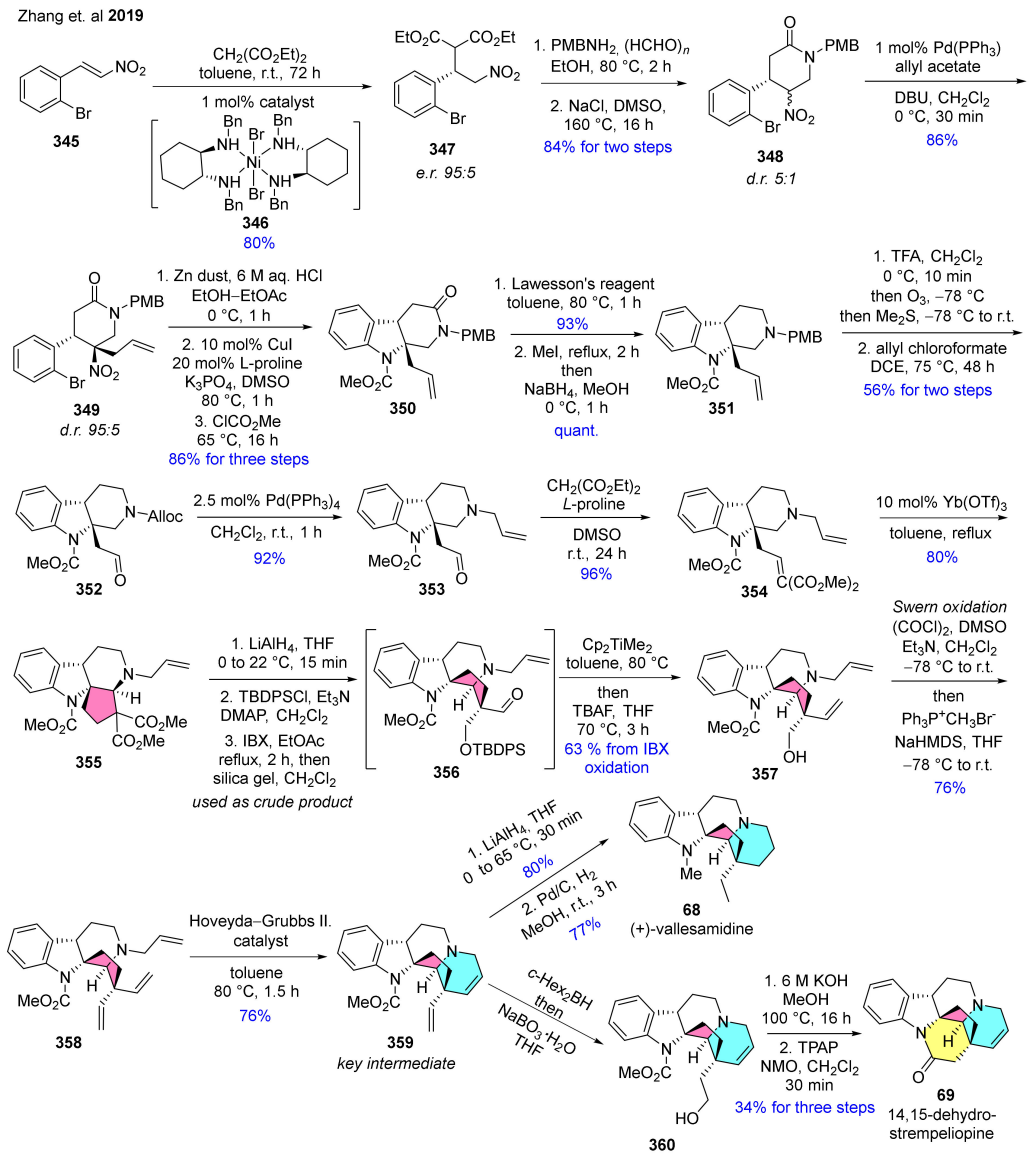
Synthesis of (+)-vallesamidine (**68**) and (+)-14,15-dehydrostrempeliopine (**69**) via key intermediate **359**.

The total synthesis of (+)-vallesamidine (**68**) in 23 steps and that of (+)-14,15-dehydrostrempeliopine (**69**) in 24 steps were completed by Anderson and co-workers through an asymmetric synthesis using the tetracyclic key intermediate **359** (Scheme 44) [161]. For the preparation of target molecules, the synthesis starts with asymmetric Michael addition with 2-bromonitrostyrene **345** using chiral Ni(II)-complex **346** as the catalyst [162]. Next, a nitro-Mannich and lactamization cascade was accomplished, which continued with a Krapcho decarboxylation to give the nitro lactam **348**. Then, a palladium-catalyzed Tsuji–Trost allylation was performed, which was followed by a nitro reduction and an intramolecular C–N coupling to construct the basic tricyclic β-carboline skeleton on gram scale (from **348** to **350**). After that, **350** underwent an in situ thionium ion formation by the sequential addition of Lawesson’s reagent, MeI and NaBH_4_. Protonation of the basic piperidine nitrogen with trifluoroacetic acid and ozonolysis gave the aldehyde, which was treated with allyl chloroformate to obtain **352**. Subsequent treatment of allyl carbamate **352** with catalytic Pd(PPh_3_)_4_ induced the decarboxylative allylation to give **353**. This was followed by the Knoevenagel condensation of **353** with dimethyl malonate to afford the α,β-unsaturated malonate **354**. Using precursor **354** in the reaction with Yb(OTf)_3_, a Lewis acid-catalyzed [1,4]-hydride transfer and Mannich-type cascade cyclization was performed, which resulted in compound **355** containing the appropriate E-ring. The D-ring with the C14=C15 double bond was installed via alkene formation through diastereoselective ring-closing metathesis (from **355** to **358**). To form the metathesis precursor from **355**, the reduction of this malonate to the corresponding diol was carried out. The diol was selectively protected, then the oxidation of the other OH-group was performed, which was followed by an olefination and the removal of the protecting group. After that, Swern oxidation and Wittig reaction took place to furnish the appropriate trialkene. **358**. Then **358** was introduced to the Hoveyda–Grubbs catalyst and the subsequent metathesis reaction occurred to afford key intermediate **359**. From intermediate **359**, the target molecules were formed via two different pathways: **68** was obtained after simple functional group transformations, while **69** was formed by further functionalization and ring closure reaction of **360**. 

**Scheme 45:**
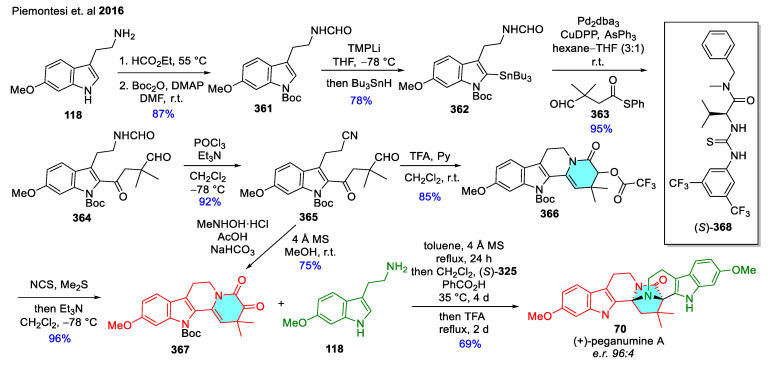
First total synthesis of (+)-peganumine A (**70**).

The enantioselective total synthesis of (+)-peganumine A (**70**) was achieved by Zhu and co-workers in 7 steps by applying multiple bond formation methods (Scheme 45) [163]. The synthesis of the octacyclic complex natural product starts from readily available 6-methoxytryptamine (**118**). By combining functional group transformation and a Liebeskind–Srogl cross-coupling (**361** into **364**), this starting material was transformed into advanced intermediate **365**. Further reactions from **365** led to the construction of tetracyclic intermediate **367** via one-pot C–C bond forming lactamization and transannular condensation. Finally, a one-pot chiral (*S*)-**368** thiourea/benzoic acid-catalyzed domino reaction merged the two achiral building blocks (**118**, **367**) into the octacyclic compound (**70**) via enantioselective Pictet–Spengler reaction followed by TFA-catalyzed transannular cyclization.

**Scheme 46:**
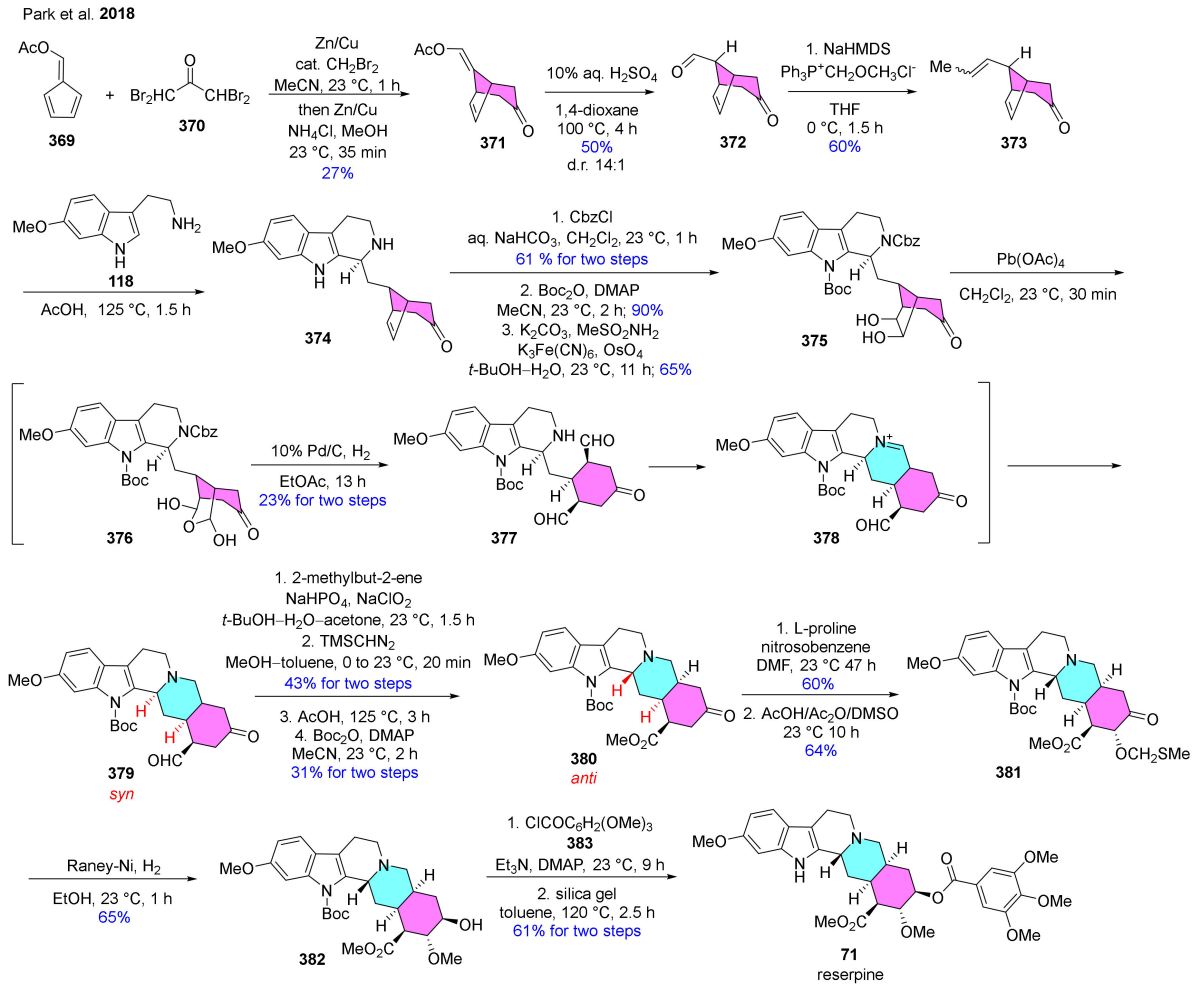
Synthesis of reserpine (**71**).

Reserpine (**71**) is a well-known drug for the treatment of high blood pressure. From the first total synthesis of **71** reported by Woodward and co-workers, [164] numerous synthetic approaches have been reported [165]. Recently, a new total synthesis of reserpine (**71**) has been accomplished using a desymmetrization protocol, which applied a simplified nonstereogenic E-ring precursor [166]. The developed strategy constitutes an unconventional form of remote stereoinduction. Late-stage functionalization of the reserpine E-ring, including a contra-steric C17 oxygenation and a stereoselective C18 ketone reduction, also played pivotal roles in completing the synthesis. As shown in Scheme 46, the synthesis began with the preparation of the bicyclic ketoaldehyde (**372**) through a reductive [4+3] cycloaddition between acetoxyfulvene (**369**) and 1,1,3,3-tetrabromoacetone (**370**) affording bicyclic enolacetate **371**, followed by an enolacetate hydrolysis with aldehyde epimerization. One-carbon homologation of **372** was performed by engaging its aldehyde functionality resulting in the methyl enol ether mixture of geometrical isomers **(373**) as an E-ring precursor. This was followed by an improved Pictet–Spengler protocol in the reaction with tryptamine derivative **118** to construct the THβC skeleton-containing compound **374**. The N atoms of **374** were protected with Cbz and Boc. At this stage, a desymmetrization protocol was performed starting with an OsO_4_-catalyzed alkene dihydroxylation in the presence of K_3_Fe(CN)_6_ to afford diol **375**. Intermediate **375** was then treated with Pb(OAc)_4_ to form a mixture where the bis-hemiacetal **376** was the predominant compound instead of its parent dialdehyde. This mixture went through a Pd/C-mediated hydrogenation, and Cbz group removal where the only detectable product was dialdehyde **377**. Next, an intramolecular iminium formation took place induced by an in-situ hydrogenation resulted **378**. After that, the irreversible dehydrogenation and the single stereoisomer suggested the iminium formation took place by selectively engaging one of the two diastereotopic aldehyde *sin*-**379**. Intermediate **379** went through various transformations including Pinnick oxidation and esterification, HOAc catalyzed epimerization and re-protecting with Boc_2_O, resulting in **380**, which was followed by a regio- and stereoselective C17 oxygenation with *L*-proline/nitrosobenzene to complete the substitution pattern required for the E-ring. Pummerer-type conditions (DMSO, Ac_2_O) were applied to furnish methylthio methyl ether intermediate **381**, which underwent a reductive desulfurization to prepare the oxygen methylated alcohol **382**. To complete the synthesis, **382** was reacted with 3,4,5-trimethoxybenzoyl chloride (**383**) and removal of the Boc-carbamate by heating in the presence of silica gel to achieve the target molecule reserpine (**71**).

**Scheme 47:**
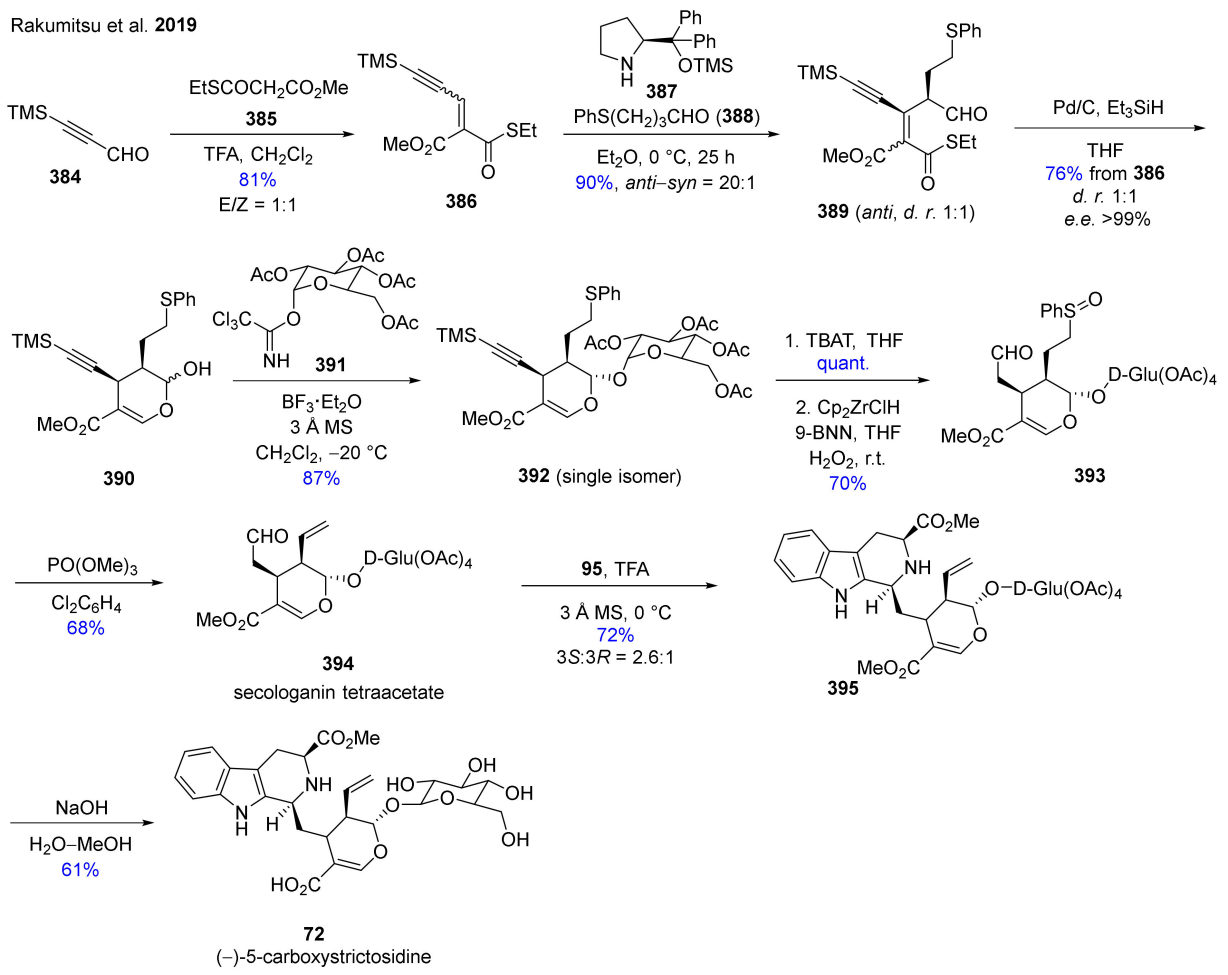
Total synthesis of (−)-5-carboxystrictosidine (**72**).

**Scheme 48:**
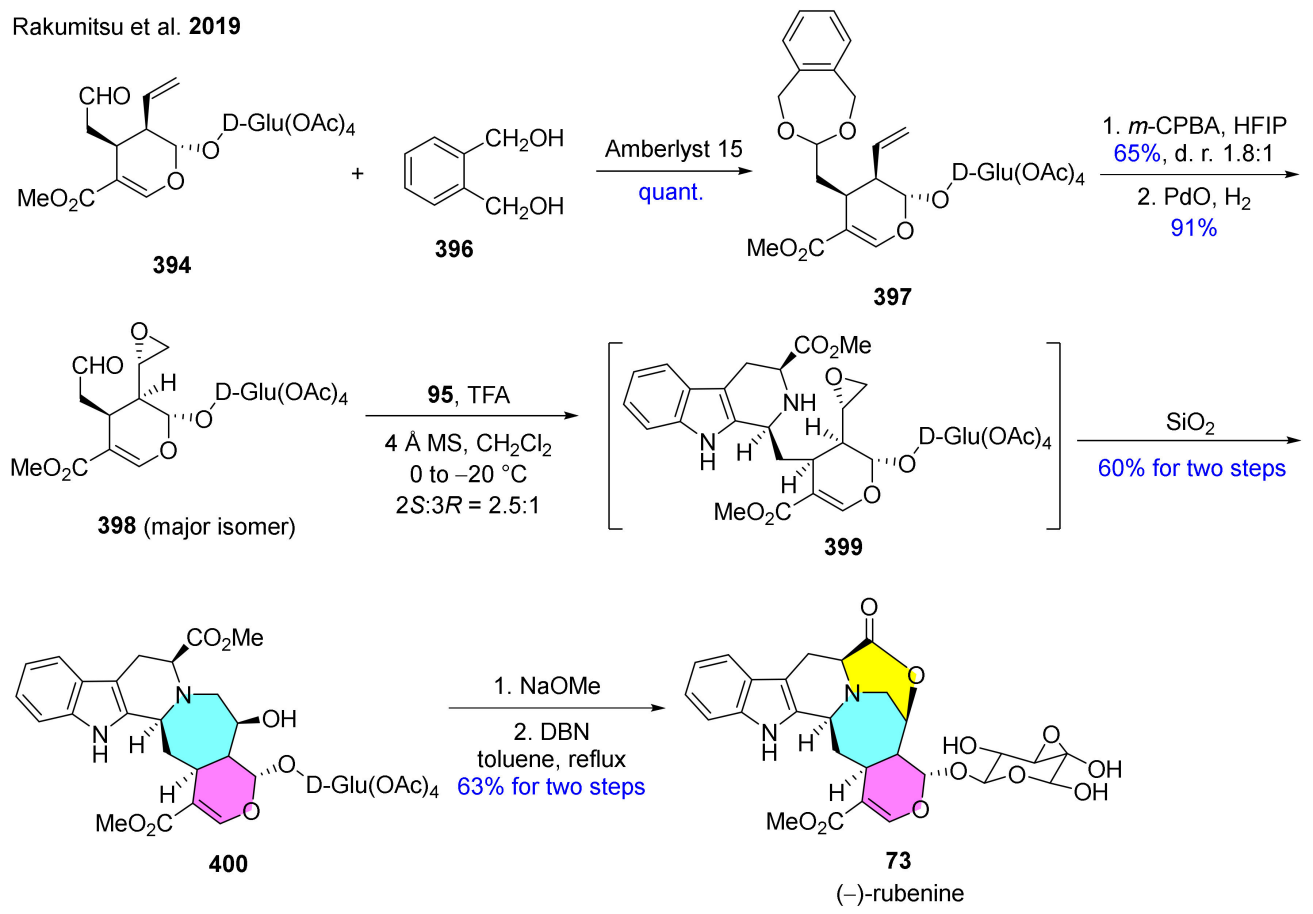
Total synthesis of (−)-rubenine (**73**).

The first enantioselective synthesis of glycosylated monoterpenoid alkaloids (–)-5-carboxystrictosidine (**72**, Scheme 47) and (−)-rubenine (**73**, Scheme 48) was accomplished by Rakamitsu et. al. [167]. The innovative part of this publication is a sequential anti-selective organocatalytic Michael reaction continued with a Fukuyama reduction and finished by a cyclization to form an optically active dihydropyran ring. The first stage of the synthesis targeted the preparation of hemiacetal **390**. First, thioester derivative **386** was prepared by Knoevenagel condensation from the readily available 3-trimethylsilylpropanal (**384**) and reagent **385**, applying a known literature method [168]. This was followed by the innovative key step, the anti-selective Michael reaction, which was finally performed with organocatalyst **387** and thioaldehyde **388** in Et_2_O solvent. The formation of the appropriate dihydropyran ring-containing key intermediate **390** was finally achieved applying Fukuyama’s protocol with Pd/C and Et_3_SiH, followed by a spontaneous cyclization. Next the glycosylation of hemiacetal diastereomeric mixture **390** was examined through the reaction with imidate **391**. The key issue of this step was to prepare **392** with controlled stereoselectivity. The silyl group removal was accomplished with TBAT, thereafter the hydroboration/oxidation was performed with Schwartz’s reagent (Cp_2_ZrClH) in the presence of 9-BNN. In this reaction the thioether was also oxidized to sulfoxide **393**, which was essential for the next sulfoxide elimination reaction. To prepare the terminal olefin bond containing moiety in **394**, Tietze’s sulfoxide elimination protocol [169] was used which resulted in another key molecule of the synthesis route called secologanin tetraacetate (**394**). To complete the total synthesis of **72**, a diastereoselective Pictet–Spengler reaction was performed [170] with **95**. Finally, hydrolysis of the acetate groups of **395** led to target molecule **72**.

In the same publication, the authors used key intermediate **394** also for the first total synthesis of (−)-rubenine (**73**, Scheme 48). Initially, aldehyde **394** was converted into cyclic acetal **397** upon reaction with 1,2-phenylenedimethanol (**396**). Then, the diastereoselective epoxidation and acetal removal of **397** was accomplished in the reaction with *m*-CPBA in HFIP solvent to obtain intermediate **398**. Next, the Pictet–Spengler reaction of **395** with **95** generated the desired 3*S*-isomer **399**. The E-ring formation was induced by the direct loading of the diastereomeric mixture on silica gel to obtain intermediate **400**. The synthesis of (−)-rubenine (**73**) was completed by the removal of the acetate moieties by methanolysis, followed by the highly strained d-ring formation upon basic lactonization reaction.

## 4. Conclusions

This review summarizes the recently reported syntheses of naturally occurring β-carbolines and closely related synthetic derivatives through the selected examples of 73 natural products. It is evident that over the last 5 years this field of alkaloid chemistry has experienced many new and novel synthetic routes, including innovative strategies for the construction of the title framework. Not only the applied methods and synthesized products are interesting for organic chemists, but they also represent a remarkable pharmacological potential. The urgent need for new drug candidates is the driving force of the development highlighted in this review. We believe that the presented synthetic methods give a valuable insight into β-carboline chemistry, while providing inspiration and encouraging further explorations in this family of compounds.

## Figures and Tables

**Table 1:** Synthesized β-carbolines with their natural occurrence and bioactivity.

ID	Name	Structure	Occurrence	Bioactivity
**1**	norharman(β-carboline)	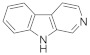	*Noctiluca milaris* [10] *Strychnos johnsonii* [11] *Lycoris longituba* [12] *Selaginella delicatula* [13] *Trichosanthes kirilowii Maxim* [14] *Rauwolfia caffra* [15] *Didemnum* sp. [16]	MAO-B inhibitor11]
**2** **3** **4** **5** **6**	(−)-tetrahydroharman/ (S)-eleagnine (−)-komaroidine *N*-(+)-methyltetrahydro-harman (+)-1-ethyl-9-methyl-tetrahydro-β-carboline (+)-*N*-acetyl-komarodine	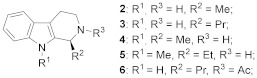	**2**: *Albizia polyphylla* [17] **3**, **6**: *N. komarovii and N.schoberi* [18] **4**: *Petalostyles labicheoides* [19] **5**: analogues of natural products	not reported
**7**	stolonine C	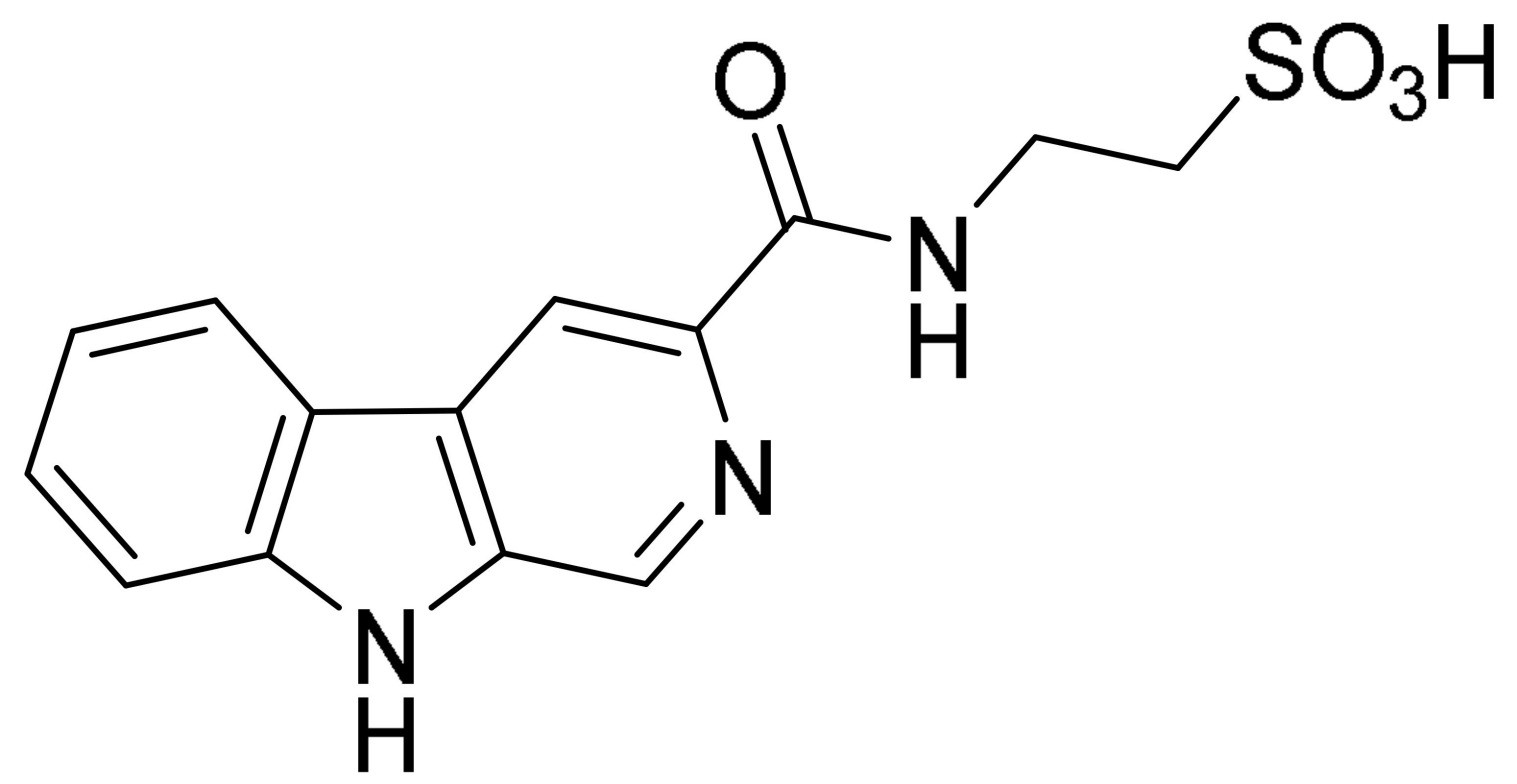	*Cnemidocarpa* stolonifera [20]	apoptosis in PC3 human prostate cancer cell line [20]
**8** **9** **10** **11** **12**	eudistomin I eudistomin N eudistomin U eudistomin T isoeudistomin U	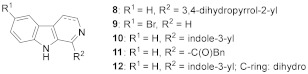	**8:***Eudistoma olivaceum* [21] **9**: *Eudistoma olivaceum* [22] **10**: *Lissoclinum fragile* [23] **11**: *Eudistoma olivaceum* [24] **12**: analogues of natural products	**8**: antibacterial effect [25] **9**: antiviral, antibacterial and antifungal activity [26] **11**: antibiotic activity [27]
**13** **14** **15** **16**	harmalan isoeudistomin M harman kumujian C	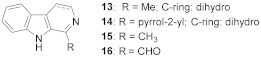	**13**: *soybean* [28] *Flindersia laevicarpa* [29] **14**: analogues of alcaloids **15**: *Flindersia Caevicarpa* [29] **16**: *Picrasma quassioides* [30]	**13**: anti-neuroin-flammatory activities [28] **14**: n.a. **15**: vasorelaxant effect [31] binding affinity to imidazoline I_2B_ receptors [32] **16**: cytotoxic activity [33] AMP phosphodiesterase inhibitor [34] anti-inflammatory agent [35,36]
**17** **18** **19** **20** **21**	trigonostemine A trigonostemine B trigonostemine G pityriacitrin hyrtiosulawesine	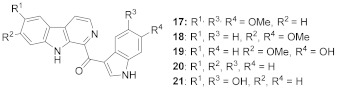	**17**, **18**: *Trigonostemone lii* [37] **17**, **18**, **19**: *Trigonostemone filipes* [38] **20**: *Malassezia furfur* [39] *Paracoccus marine bacteria* [40] *Dichotomomyces cejpii* [41] **21**: *Hyrtio erectus* [42] *Alocasia macrorrhiza* [43]	**17**, **18**, **19**: cytotoxic activity [44] **20**: antiproliferative activity [45] anticancer activity [46,47] **21**: antiproliferative activity [48,49] antimalarial activity [46] antiphospholipase A2 [50] and antioxidant activity [46]
**22** **23**	alangiobussine alangiobussinine	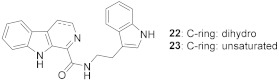	*Alangium bussyanum* [51]	not reported
**24**	haploscleridamine	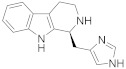	*Sponge of the order Haplosclerida* [52]	inhibitor of cathepsin K [52]
**25**	(±)-peharmaline A	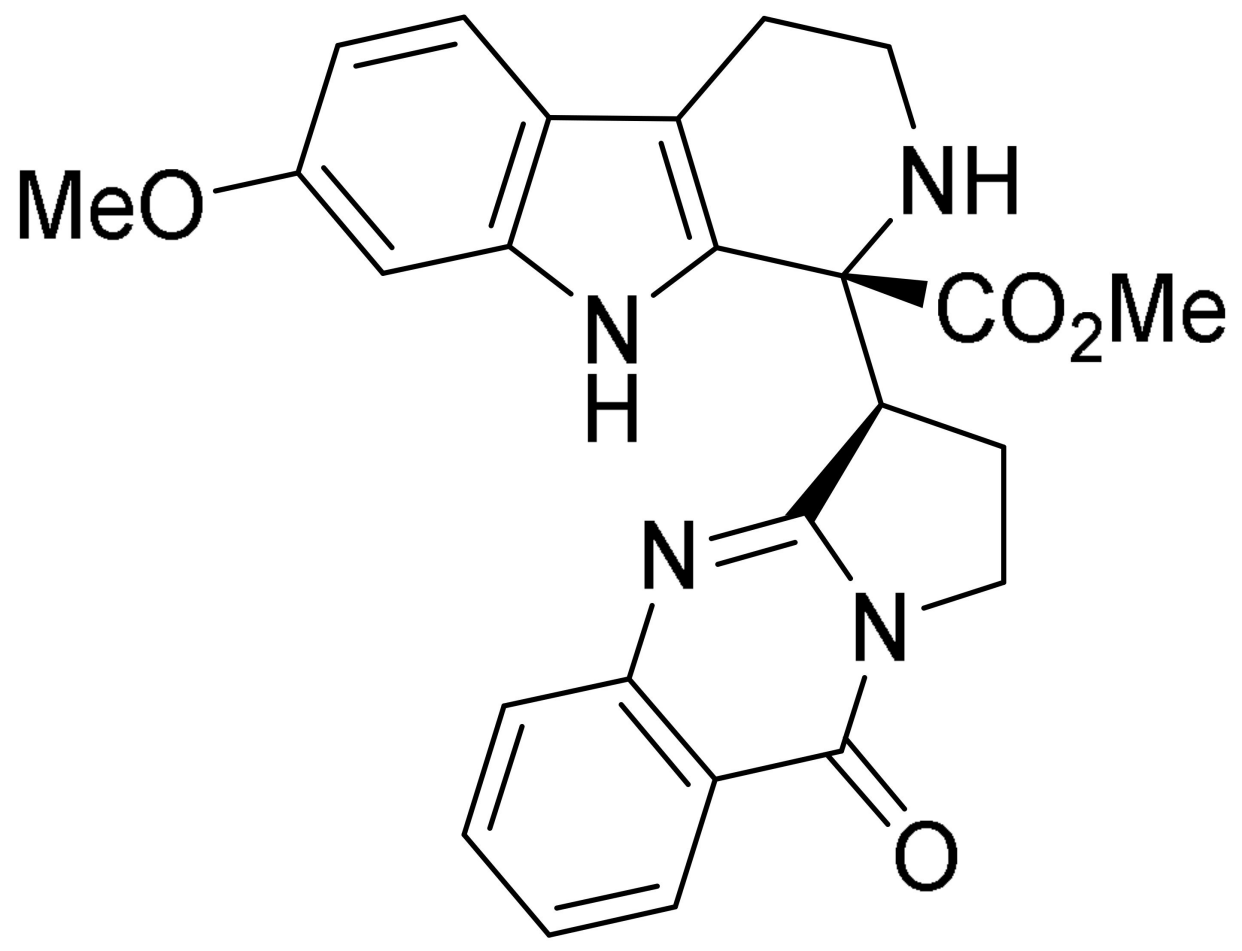	*Peganum harmala* [53]	cytotoxic activity (HL-60, PC-3, SGC-7901) [54]
**26**	komavine	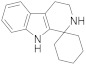	*Nitraria komarorii* [55] *Nitraria schoberi* [55]	not reported
**27** **28** **29** **30**	marinacarboline A marinacarboline B marinacarboline C marinacarboline D	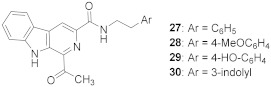	*Marinactinospora thermotolerans* SCSIO 00652 [56]	antiplasmodial activity [56] cytotoxic activity [57]
**31**	metatacarboline A	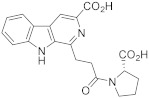	*Mycena metata* [58]	not reported
**32** **33** **34** **35**	metatacarboline C metatacarboline D metatacarboline E metatacarboline F	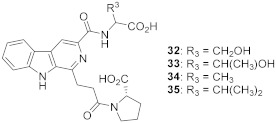	*Mycena metata* [58]	**32**, **34**: antiproliferative activity, proapoptotic effect [59]
**36**	6-hxdroxy- metatacarboline D	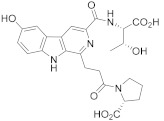	*Mycena metata* [58]	not reported
**37**	shishijimicin A	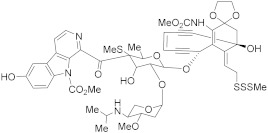	*Didemnum proliferum* [60]	antitumor activity (P388 leukemia cells) [60]
**38**	canthine	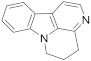	basic framework of canthin-6-ones, which are widely distributed in nature [61]	not reported
**39**	harmicine	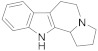	*Kopsia griffithii* [62]	not reported
**40**	cordatanine	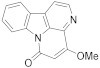	*Drymaria cordata* [63] *Drymaria diandra* [64,65]	anti-HIV activity [64]
**41**	griseofamine A	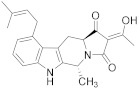	*Penicillium griseofulvum* [66]	antibacterial activity [67]
**42**	(+)-deplancheine	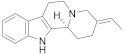	*Alstonia deplanchei* [68]	not reported
**43**	(−)-geissoschizol	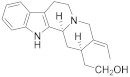	*Tabernaemontana bufalina* [69] *Hunteria zeylanica* [70]	not reported
**44**	chaetogline E	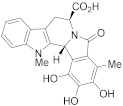	*modified Epinephelus drummondhayi with a silent fungal gene from Chaetomium globosum* [71]	not reported
**45**	chaetogline F	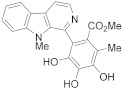	*modified Epinephelus drummondhayi with a silent fungal gene from Chaetomium globosum* [71]	antibacterial activity [71]
**46**	6-oxofascaplysin	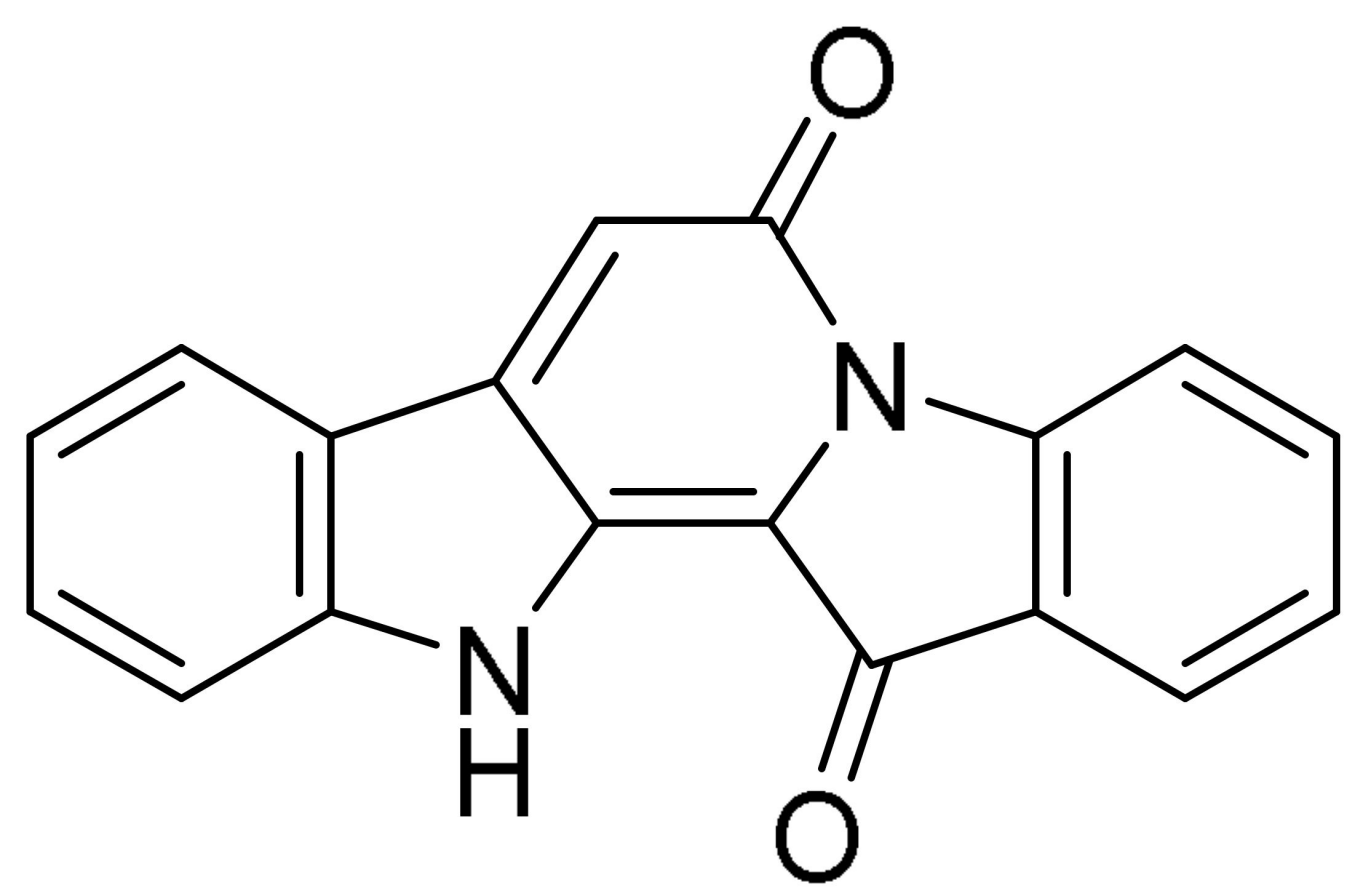	*Hyrtios sp.* [72]	weak cytotoxic activity [73]
**47**	fascaplysin	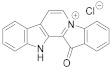	*Fascaplisynopsis sp* [74]	anticancer activity [73,75]
**48**	evodiamine	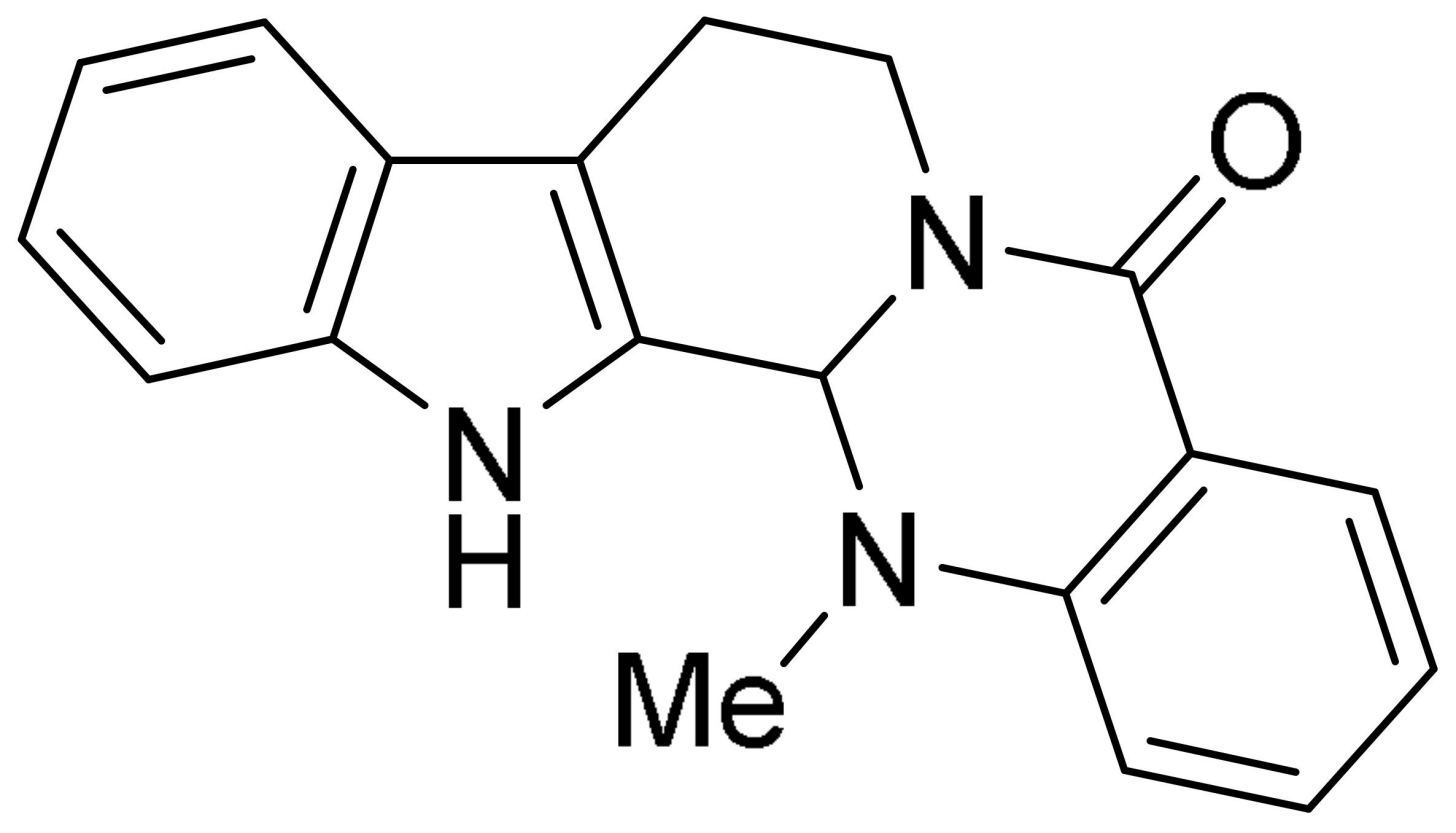	*Evodia rutaecarpa* [76]	anticancer anti-inflammatory antimicrobial and many others [77]
**49** **50** **51**	norsuaveoline suaveoline marcophylline	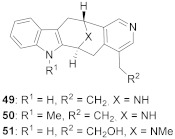	**49:***Rauvolfia caffra* [78] **50:** *Rauwolfia suaveolens S. Moore (apocynaceae)* [79] **51**: *Rauwolfia macrophylla* [80]	**49**: not reported **50**: acetylcholinesterase inhibitor [81] **51**: not reported
**52**	(*S*)-(−)-decarbo-methoxy-dihydro-gambirtannine	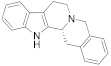	enantiomer of **52** was isolated from *Hunteria zeylanica* [82]	not reported
**53**	(+)-strictamine	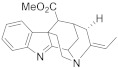	*Alstonis scholaris* *Rhazya strictu* [83,84]	antitumor, antiviral anti-inflammatory antibiosis activities [83,84,85]
**54**	(±)-arbornamine	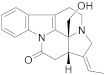	*Kopsia arborea* [86]	not reported
**55**	(+)-tacamonine	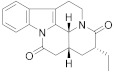	*Tabernaemontana eglandulosa* [87]	not reported
**56**	(−)-17-nor-excelisidine	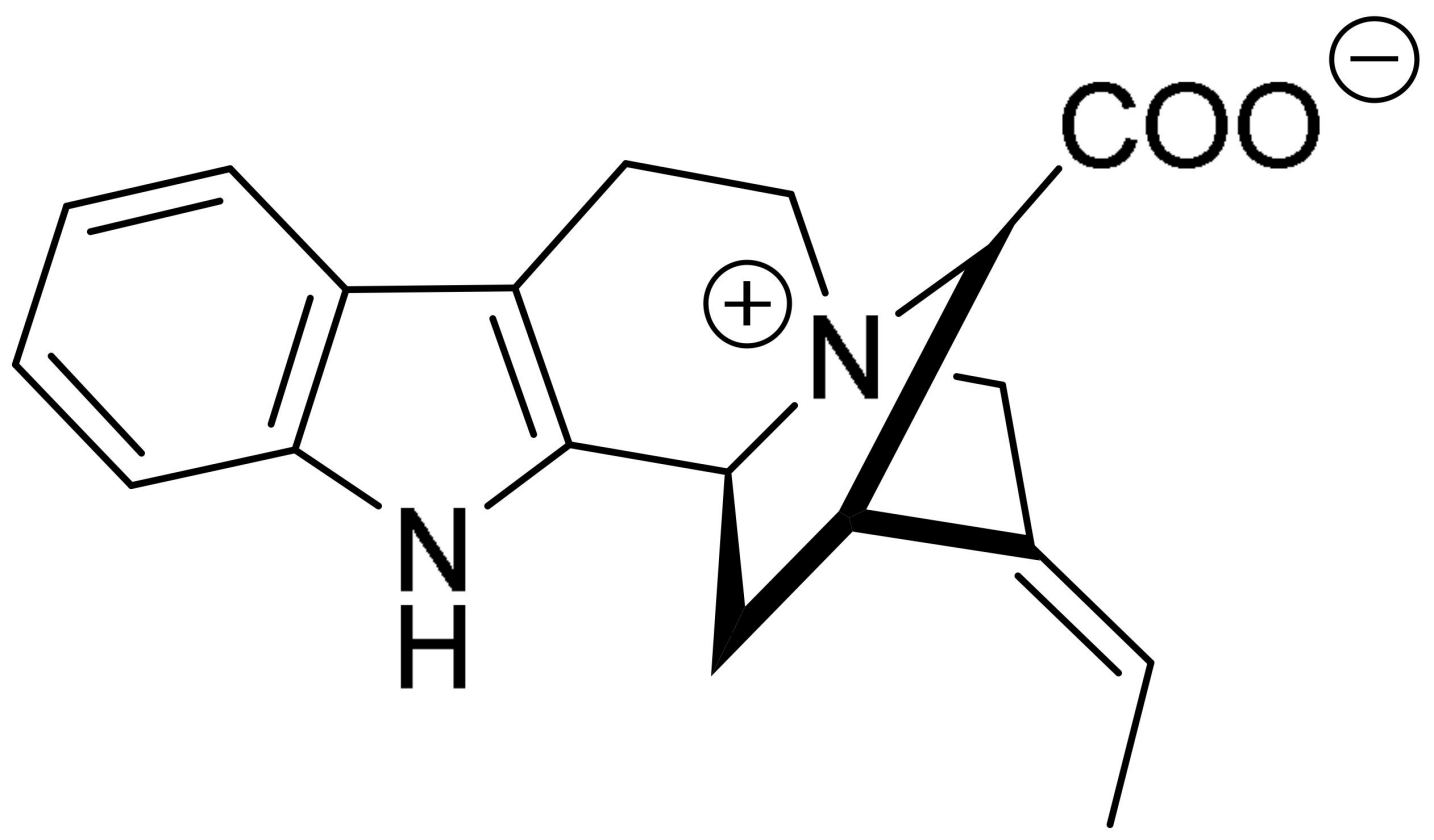	*Alstonia scholaris* [85]	antiviral activity [85]
**57**	(+)-geissoschizine	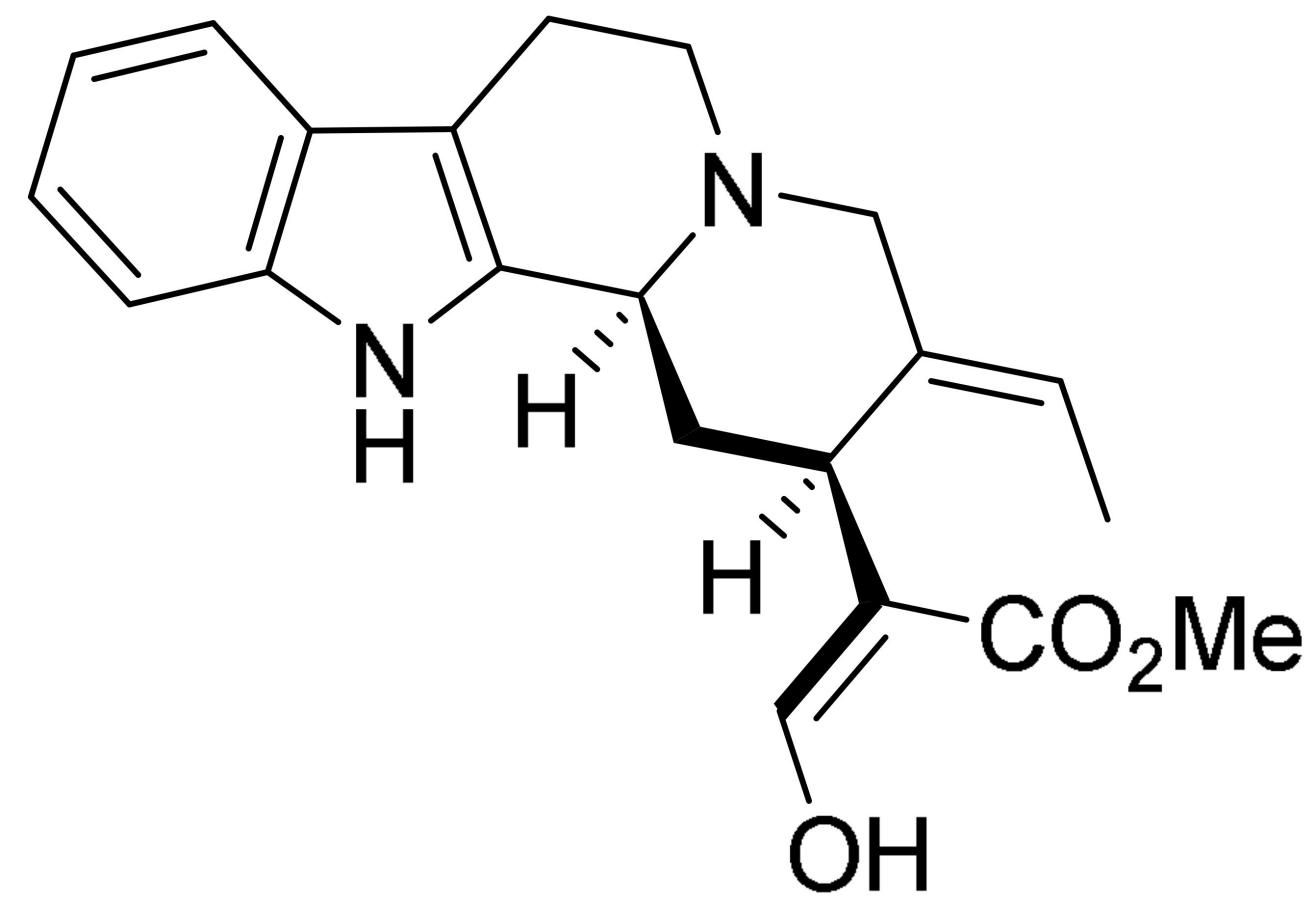	*Rhazya stricta* [88]	not reported
**58** **59** **60** **61** **62** **63**	(+)-16-epi-pleiocarpamine (+)-taberdivarine H (+)-16-hydroxymethyl-pleiocarpamine (+)-pleiocarpamine (±)-normavacurine (±)-*C*-mavacurine	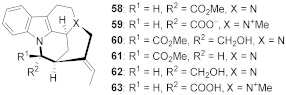	**58**: *Ervatamia cumingiana* [89] *Pleiocarpa pycnantha* [90] **59***: Tabernaemontana officinalis* [91] **60**: *Kopsia singapurensis* [92] *Kopsia teoi* [93] **62***: Alstonia scholaris* [94] **63**: *Strychnos guianensis* [95]	**61, 58**: anticholinergic activity [96] **59**: cytotoxic activity [91] **60**: not reported **62**: antibacterial activity [94] **63**: not reported
**64** **65** **66** **67**	(+)-19-oxoeburnamine (+)-19-OH-eburnamine 19-(*S*)-hydroxy-Δ^14^-vincamone (–)-19-hydroxy-Δ^14^-eburnamonine	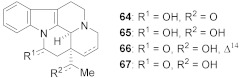	*Kopsia jasminiflora* [97] *Kopsia pauciflora* [98]	not reported closely related analogues show antitumor activity
**68**	(+)-vallesamidine	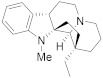	*Vallesia dichotoma* [99]	not reported
**69**	(+)-14,15-dehydro-strempeliopine	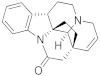	*Schizozygia caffaeoides* [100]	not reported
**70**	(+)-peganumine A	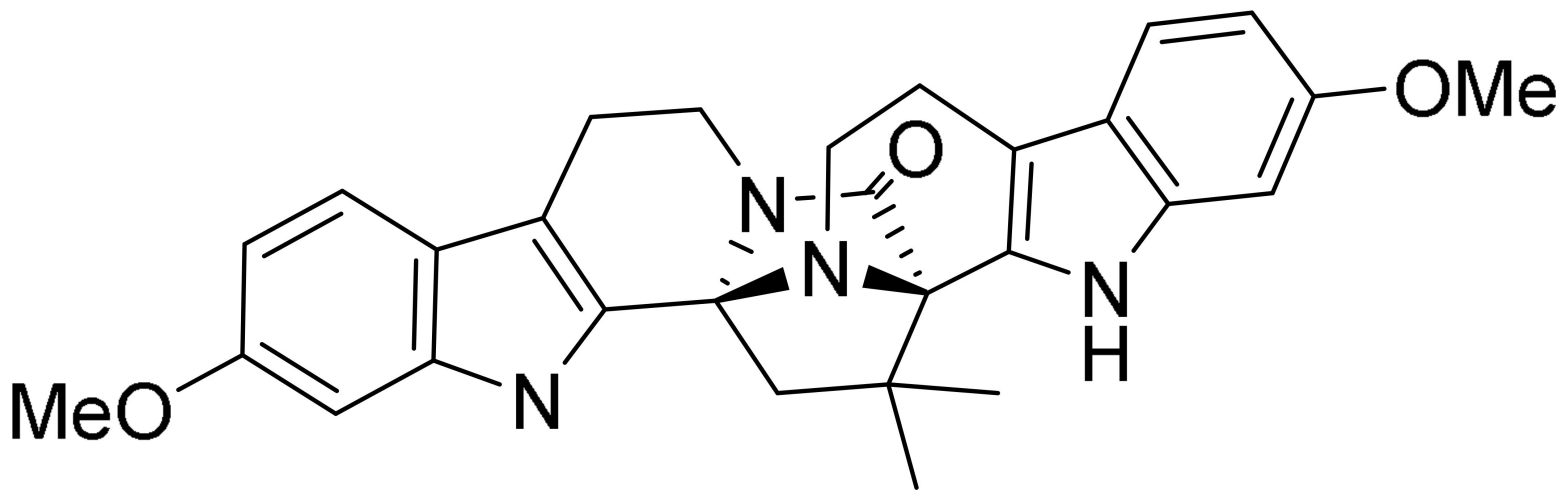	*Peganum harmala* L. [101]	cytotoxic activity (MCF-70, PC-3, HepG2, HL-60) [101]
**71**	reserpine	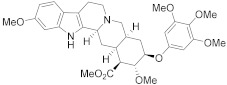	*Rauwolfia serpentina* [102]	Commercially available drug for the treatment of high blood pressure [103]
**72**	(–)-5-carboxystrictosidine	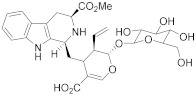	*Rhazya orientalis* [104] *Guenttarda platypoda* [105] *Uncaria tomentosa* [106] *Ophiorrhiza nutans* [107]	not reported
**73**	(–)-rubenine	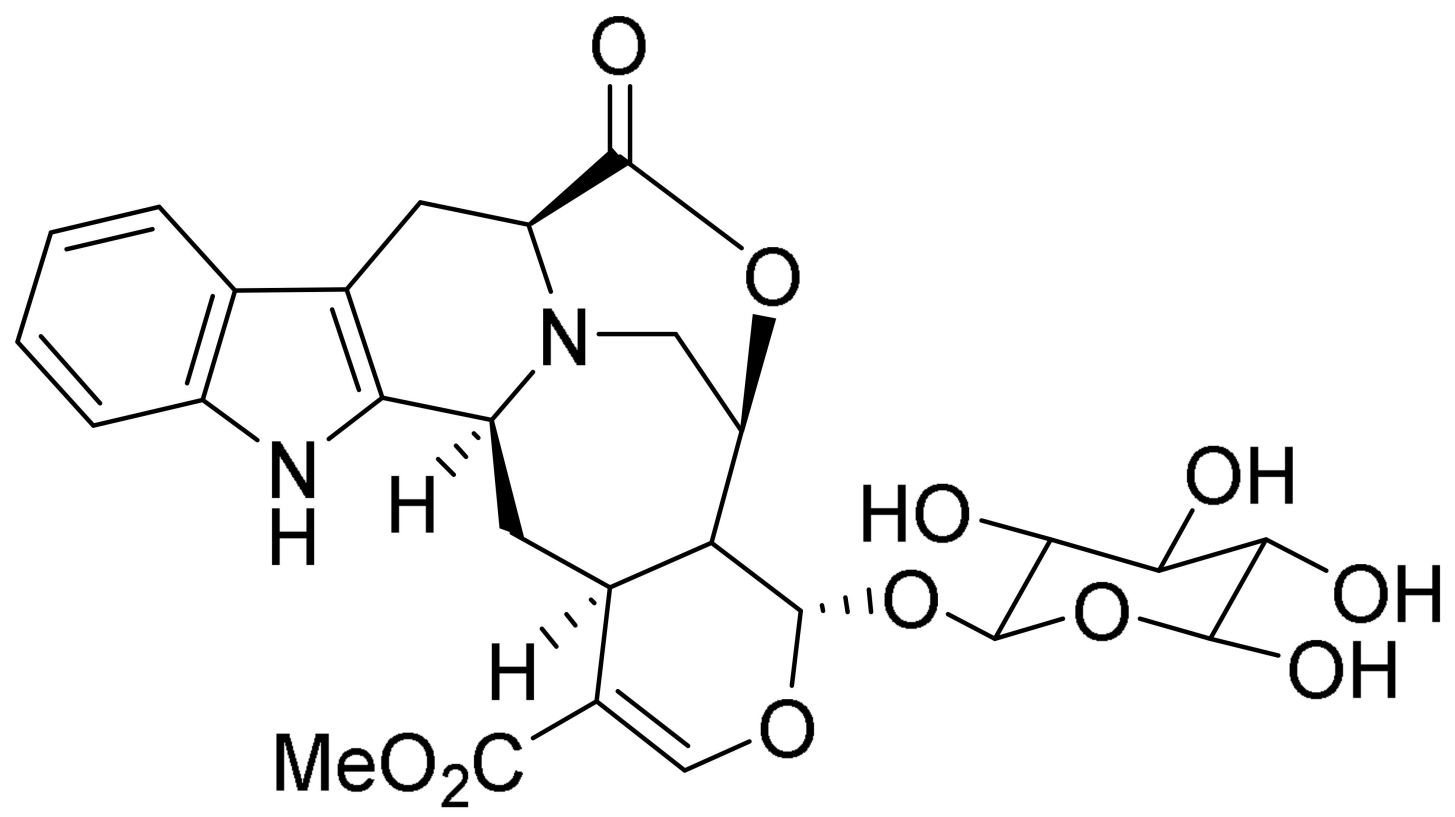	*Adina rubescens* [108]	not reported

## Abbreviations

List of Abbreviations and Named reagents.

9-BNN	9-borabicyclo[3.3.1]nonane
Å	angstrom
Ac	acetyl
ACCN	1,1′-azobis(cyclohexanecarbo-nitrile)
AIBN	azobisisobutyronitrile
Alloc	allyloxycarbonyl
aq	aqueous
Barton’s base	2-*tert*-butyl-1,1,3,3-tetra-methylguanidine
Bn	benzyl
Boc	*tert*-butyloxycarbonyl
brsm	yield based on recovered starting material
BuLi	*n*-butyllithium
Bz	benzoyl
CAN	cerium ammonium nitrate
cap	caprolactamate
CDI	carbonyldiimidazole
*c*-Hex_2_BH	dicyclohexylborane
cod	cyclooctadiene
conc.	concentrated
Cp_2_TiMe_2_	bis(η^5^-cyclopentadienyl)-dimethyl-titanium
CuDPP	copper(I)-diphenyl phosphinate
d	day
d.r.	diastereomeric ratio
DABCO	1,4-diazabicyclo[2.2.2]octane
dba	dibenzylideneacetone
DBU	1,8-diazabicyclo[5.4.0]undec-7-ene
DBN	1,5-diazabicyclo[4.3.0]non-5-ene
DCC	*N*,*N*′-dicyclohexylcarbodiimide
DDQ	2,3-dichloro-5,6-dicyano-1,4-benzoquinone
DEAD	diethyl azodicarboxylate
DHβC	3,4-dihydro-β-carboline
DIBAL	diisobutylaluminum hydride
DIPEA	*N*,*N*-diisopropylethylamine
DMAP	4-dimethylaminopyridine
DME	1,2-dimethoxyethane
DMF	dimethylformamide
DMP	Dess–Martin periodinane
DMS	dimethyl sulfide
DMSO	dimethyl sulfoxide
e.e.	enantiomeric excess
e.r.	enantiomeric ratio
EDCl	1-ethyl-3-(3-dimethylaminopropyl)-carbodiimide
Et	ethyl
h	hour
HFIP	1,1,1,3,3,3-hexafluoroisopropyl alcohol
HOBt	1-hydroxybenzotriazole
IBA	2-iodosobenzoic acid
IBX	2-iodoxybenzoic acid
KHMDS	potassium bis(trimethylsilyl)-amide
Lawesson’s reagent	2,4-bis(4-methoxy-phenyl)-1,3,2,4-dithiadiphosphetane-2,4-disulfide
LDA	lithium diisopropylamide
LiHMDS	lithium bis(trimethylsilyl)amide
*L*-selectride	lithium tri-*sec*-butyl(hydrido)-borate
*m*-CPBA	*meta*-chloroperbenzoic acid
Me	methyl
MEM	2-methoxyethoxymethyl ether
min.	minute
MS	molecular sieve
MW	microwave
Nap	2-naphthylmethyl ether
NB	2-nitrobenzyl
NBS	*N*-bromosuccinimide
NCS	*N*-chlorosuccinimide
NMM	*N*-methylmorpholine
NMO	4-methylmorpholine *N*-oxide
NMP	*N*-methyl-2-pyrrolidone
Ns	2-nitrobenzenesulfonyl
*o*-NBOH	*o*-nitrobenzylalcohol
Oxone^®^	2 KHSO_5_·KHSO_4_·K_2_SO_4_
PDC	pyridinium dichromate
Pf	phenylfluorenyl
Phth	phthaloyl
pin	pinacolato
PMB	*p*-methoxybenzyl
Pr	propyl
psi	pound-force per square inch
Py	pyridine
r.t.	room temperature
Schwartz’s reagent	chloridobis(η^5^-cyclo-pentadienyl)hydridozirconium
T3P^®^	propylphosphonic anhydride
TBAF	tetrabutylammonium fluoride
TBAT	tetrabutylammonium difluoro-triphenylsilicate
TBDPS	*tert*-butyldiphenylsilyl
TBDMS	*tert*-butyldimethylsilyl
*t*-Bu	*tert*-butyl
TES	triethylsilane
TMG	tetramethylguanidine
Tf	trifluoromethanesulfonyl
TFA	trifluoroacetic acid
TFAA	trifluoroacetic anhydride
THF	tetrahydrofuran
THβC	1,2,3,4-tetrahydro-β-carboline
TIPS	triisopropylsilyl
TMG	1,1,3,3-tetramethylguanidine
TMP	2,2,6,6-tetramethylpiperidine
TMS	trimethylsilyl
TPAP	tetrapropylammonium perruthenate
Ts	*p*-toluenesulfonyl (tosyl)
Tsdpen	*N*-tosyl-1,2-diphenylethylene-1,2-diamine

## References

[1] Smirnova O.B., Golovko T.V., Granik V.G. (2011). Carbolines. Part 2: Comparison of some of the properties of α-, γ-, and δ-carbolines. Pharm. Chem. J..

[2] Singh V., Batra S. (2012). 1-Formyl-9*H*-β-carboline: A useful scaffold for synthesizing substituted and fused β-carbolines. Curr. Org. Synth..

[3] Piechowska P., Zawirska-Wojtasiak R., Mildner-Szkudlarz S. (2019). Bioactive β-carbolines in food: A review. Nutrients.

[4] Dai J., Dan W., Schneider U., Wang J. (2018). β-Carboline alkaloid monomers and dimers: Occurrence, structural diversity, and biological activities. Eur. J. Med. Chem..

[5] Casal S. (2019). Chapter 20. Potential effects of β-carbolines on human health. Coffee: Consumption and Health Implications.

[6] Cao R., Peng W., Wang Z., Xu A. (2007). b-Carboline alkaloids: Biochemical and pharmacological functions. Curr. Med. Chem..

[7] Maity P., Adhikari D., Jana A.K. (2019). An overview on synthetic entries to tetrahydro-β-carbolines. Tetrahedron.

[8] Domínguez G., Pérez-Castells J. (2011). Chemistry of β-carbolines as synthetic intermediates. Eur. J. Org. Chem..

[9] Devi N., Kumar S., Pandey S.K., Singh V. (2018). 1(3)-Formyl-β-carbolines: Potential aldo-X precursors for the synthesis of β-carboline-based molecular architectures. Asian J. Org. Chem..

[10] Inoue S., Okada K., Tanino H., Kakoi H., Goto T. (1980). Trace characterization of the fluorescent substances of a dinoflagellate, *Noctiluca miliaris*. Chem. Lett..

[11] Herraiz T. (2007). Identification and occurrence of β-carboline alkaloids in raisins and inhibition of monoamine oxidase (MAO). J. Agric. Food. Chem..

[12] Zhu Y.-Y., Li X., Yu H.-Y., Xiong Y.-F., Zhang P., Pi H.-F., Ruan H.-L. (2015). Alkaloids from the bulbs of *Lycoris longituba* and their neuroprotective and acetylcholinesterase inhibitory activities. Arch. Pharmacal Res..

[13] Yao C.-P., Zou Z.-X., Zhang Y., Li J., Cheng F., Xu P.-S., Zhou G., Li X.-M., Xu K.-P., Tan G.-S. (2019). New adenine analogues and a pyrrole alkaloid from *Selaginella delicatula*. Nat. Prod. Res..

[14] Lei X., Li N., Bai Z., Di J., Zhang H., Dong P., Zhang P. (2020). Chemical constituent from the peel of *Trichosanthes kirilowii* Maxim and their NF-κB inhibitory activity. Nat. Prod. Res..

[15] Nasser A.M., Court W.E. (1983). Leaf alkaloids of *Rauwolfia caffra*. Phytochemistry.

[16] Schumacher R.W., Davidson B.S. (1995). Didemnolines A-D, new N9-substituted β-carbolines from the marine ascidian *Didemnum* sp.. Tetrahedron.

[17] Rajemiarimoelisoa C.F., Boyère C., Pellissier L., Peuchmaur M., Randrianarivo H.R., Rakoto D.A.D., Jeannoda V.L., Boumendjel A. (2016). Chemical composition of the pods of *Albizia polyphylla*. Nat. Prod. Res..

[18] Tulyaganov T., Kozimova N., Allaberdiev F.K. (2006). Alkaloids from plants of the genus *Nitraria*. Chem. Nat. Compd..

[19] Badger G.M., Beecham A.F. (1951). Isolation of tetrahydroharman from *Petalostyles labicheoides*. Nature.

[20] Tran T.D., Pham N.B., Ekins M., Hooper J.N.A., Quinn R.J. (2015). Isolation and total synthesis of stolonines A–C, unique taurine amides from the australian marine tunicate *Cnemidocarpa stolonifera*. Mar. Drugs.

[21] Hudson J.B., Saboune H., Abramowski Z., Towers G.H., Rinehart K.L. (1988). The photoactive antimicrobial properties of eudistomins from the caribbean tunicate *Eudistoma olivaceum*. Photochem. Photobiol..

[22] Tarzi O.I., Erra-Balsells R. (2005). Photochemistry of the alkaloids eudistomin N (6-bromo-nor-harmane) and eudistomin O (8-bromo-nor-harmane) and other bromo-β-carbolines. J. Photochem. Photobiol. B Biol..

[23] Badre A., Boulanger A., Abou-Mansour E., Banaigs B., Combaut G., Francisco C. (1994). Eudistomin U and isoeudistomin U, new alkaloids from the carribean ascidian *Lissoclinum* fragile. J. Nat. Prod..

[24] Kinzer K.F., Cardellina J.H. (1987). Three new β-carbolines from the bermudian tunicate *Eudistoma olivaceum*. Tetrahedron Lett..

[25] Rajesh R.P., Murugan A. (2019). Spectroscopic identification of brominated, non-brominated alkaloids and evaluation of antimicrobial activity of Eudistomin-I, Eudistomin H, from green ascidian *Eudistoma viride*. J. Appl. Pharm. Sci..

[26] Kobayashi J., Harbour G.C., Gilmore J., Rinehart K.L. (1984). Eudistomins A, D, G, H, I, J, M, N, O, P, and Q, bromo, hydroxy, pyrrolyl and iminoazepino. beta-Carbolines from the antiviral caribbean tunicate *Eudistoma olivaceum*. J. Am. Chem. Soc..

[27] Rinehart K.L., Kobayashi J., Harbour G.C., Gilmore J., Mascal M., Holt T.G., Shield L.S., Lafargue F. (1987). Eudistomins A-Q,. beta-Carbolines from the antiviral caribbean tunicate Eudistoma olivaceum. J. Am. Chem. Soc..

[28] Kim D.-C., Quang T.H., Yoon C.-S., Ngan N.T.T., Lim S.-I., Lee S.-Y., Kim Y.-C., Oh H. (2016). Anti-neuroinflammatory activities of indole alkaloids from kanjang (Korean fermented soy source) in lipopolysaccharide-induced BV2 microglial cells. Food Chem..

[29] Picker K., Ritchie E., Taylor W. (1976). The chemical constituents of australian Flindersia species. XXI. An examination of the bark and the leaves of *F. laevicarpa*. Aust. J. Chem..

[30] Guo E., Hu Y., Du T., Zhu H., Chen L., Qu W., Zhang J., Xie N., Liu W., Feng F. (2019). Effects of *Picrasma quassioides* and its active constituents on Alzheimer’s disease in vitro and in vivo. Bioorg. Chem..

[31] Shi C.-C., Chen S.-Y., Wang G.-J., Liao J.-F., Chen C.-F. (2000). Vasorelaxant effect of harman. Eur. J. Pharmacol..

[32] Miralles A., Esteban S., Sastre-Coll A., Moranta D., Asensio V.J., García-Sevilla J.A. (2005). High-affinity binding of β-carbolines to imidazoline I2B receptors and MAO-A in rat tissues: Norharman blocks the effect of morphine withdrawal on DOPA/noradrenaline synthesis in the brain. Eur. J. Pharmacol..

[33] Abdel-Moty S., Sakai S., Aimi N., Takayama H., Kitajima M., El-Shorbagi A., Ahmed A., Omar N. (1998). Synthesis of cytotoxic 1-polyhydroxyalkyl-β-carboline derivatives. Eur. J. Med. Chem..

[34] Sung Y., Koike K., Nikaido T., Ohmoto T., Sankawa U. (1984). Inhibitors of cyclic AMP phosphodiesterase in *Picrasma quassioides* Bennet, and inhibitory activities of related beta-carboline alkaloids. Chem. Pharm. Bull..

[35] Jiao W.H., Gao H., Zhao F., Lin H.W., Pan Y.M., Zhou G.X., Yao X.S. (2011). Anti-inflammatory alkaloids from the stems of *Picrasma quassioides* Bennet. Chem. Pharm. Bull..

[36] Liu P., Li H., Luan R., Huang G., Liu Y., Wang M., Chao Q., Wang L., Li D., Fan H. (2019). Identification of β-carboline and canthinone alkaloids as anti-inflammatory agents but with different inhibitory profile on the expression of iNOS and COX-2 in lipopolysaccharide-activated RAW 264.7 macrophages. J. Nat. Med..

[37] Li S.-F., Zhang Y., Li Y., Li X.-R., Kong L.-M., Tan C.-J., Li S.-L., Di Y.-T., He H.-P., Hao X.-J. (2012). β-Carboline alkaloids from the leaves of *Trigonostemon lii*. Bioorg. Med. Chem. Lett..

[38] Wang Y., Wang P., Kong F.D., Wang J., Zuo W.J., Wang H., Dai H.F., Mei W.L. (2018). Two new alkaloids from the twigs of Trigonostemon filipes. J. Asian Nat. Prod. Res..

[39] Machowinski A., Krämer H.J., Hort W., Mayser P. (2006). Pityriacitrin—A potent UV filter produced by Malassezia furfur and its effect on human skin microflora. Mycoses.

[40] Nagao T., Adachi K., Nishida F., Nishishima M., Mochida K. (1999). New Ultraviolet-Absorbing Substance Produced by Marine Bacteria and Its Production. JP Patent.

[41] Chen Y.-X., Xu M.-Y., Li H.-J., Zeng K.-J., Ma W.-Z., Tian G.-B., Xu J., Yang D.-P., Lan W.-J. (2017). Diverse secondary metabolites from the marine-derived fungus *Dichotomomyces cejpii* F31-1. Mar. Drugs.

[42] Salmoun M., Devijver C., Daloze D., Braekman J.-C., van Soest R.W. (2002). 5-Hydroxytryptamine-derived alkaloids from two marine sponges of the genus *Hyrtios*. J. Nat. Prod..

[43] Huang W., Yi X., Feng J., Wang Y., He X. (2017). Piperidine alkaloids from *Alocasia macrorrhiza*. Phytochemistry.

[44] Li S.F., Cheng Y.Y., Zhang Y., Li S.L., He H.P., Hao X.J. (2012). β-carboline alkaloids from *Trigonostemon filipes* and *Trigonostemon lii*. Nat. Prod. Bioprospect..

[45] Zhang P., Sun X., Xu B., Bijian K., Wan S., Li G., Alaoui-Jamali M., Jiang T. (2011). Total synthesis and bioactivity of the marine alkaloid pityriacitrin and some of its derivatives. Eur. J. Med. Chem..

[46] Liew L.P., Fleming J.M., Longeon A., Mouray E., Florent I., Bourguet-Kondracki M.-L., Copp B.R. (2014). Synthesis of 1-indolyl substituted β-carboline natural products and discovery of antimalarial and cytotoxic activities. Tetrahedron.

[47] Mexia N., Gaitanis G., Velegraki A., Soshilov A., Denison M.S., Magiatis P. (2015). Pityriazepin and other potent AhR ligands isolated from *Malassezia furfur* yeast. Arch. Biochem. Biophys..

[48] Zhu L.-h., Chen C., Wang H., Ye W.-c., Zhou G.-x. (2012). Indole alkaloids from *Alocasia macrorrhiza*. Chem. Pharm. Bull..

[49] Pereira M.D., da Silva T., Aguiar A.C.C., Oliva G., Guido R.V., Yokoyama-Yasunaka J.K., Uliana S.R., Lopes L.M. (2017). Chemical composition, antiprotozoal and cytotoxic activities of indole alkaloids and benzofuran neolignan of *Aristolochia cordigera*. Planta Med..

[50] Sauleau P., Martin M.-T., Dau M.-E.T.H., Youssef D.T., Bourguet-Kondracki M.-L. (2006). Hyrtiazepine, an azepino-indole-type alkaloid from the Red Sea marine sponge *Hyrtios erectus*. J. Nat. Prod..

[51] Diallo A.O., Mehri H., Iouzalen L., Plat M. (1995). Alkaloids from leaves of Alangium bussyanum. Phytochemistry.

[52] Patil A.D., Freyer A.J., Carte B., Taylor P.B., Johnson R.K., Faulkner D.J. (2002). Haploscleridamine, a novel tryptamine-derived alkaloid from a aponge of the order *Haplosclerida*:  An inhibitor of Cathepsin K. J. Nat. Prod..

[53] Wang K.B., Li S.G., Huang X.Y., Li D.H., Li Z.L., Hua H.M. (2017). (±)-Peharmaline A: A pair of rare β-carboline–vasicinone hybrid alkaloid enantiomers from *Peganum harmala*. Eur. J. Org. Chem..

[54] Wang S.G., Xia Z.L., Xu R.Q., Liu X.J., Zheng C., You S.L. (2017). Construction of chiral tetrahydro-β-carbolines: Asymmetric Pictet–Spengler reaction of indolyl dihydropyridines. Angew. Chem..

[55] Tulyaganov T.S., Nazarov O.M., Levkovich M.G., Abdullaev N.D. (2001). Alkaloids of the *Nitraria* genus. Komavine and Acetylkomavine. Chem. Nat. Compd..

[56] Huang H., Yao Y., He Z., Yang T., Ma J., Tian X., Li Y., Huang C., Chen X., Li W. (2011). Antimalarial β-carboline and indolactam alkaloids from Marinactinospora thermotolerans, a deep sea isolate. J. Nat. Prod..

[57] Li J., Tang Y., Jin H.J., Cui Y.D., Zhang L.J., Jiang T. (2015). An efficient synthesis method targeted to marine alkaloids marinacarbolines A-D and their antitumor activities. J. Asian Nat. Prod. Res..

[58] Jaeger R.J., Lamshöft M., Gottfried S., Spiteller M., Spiteller P. (2013). HR-MALDI-MS imaging assisted screening of β-carboline alkaloids discovered from *Mycena metata*. J. Nat. Prod..

[59] Naveen B., Mudiraj A., Khamushavalli G., Babu P.P., Nagarajan R. (2016). Concise total synthesis of water soluble metatacarboline A, C, D, E and F and its anticancer activity. Eur. J. Med. Chem..

[60] Oku N., Matsunaga S., Fusetani N. (2003). Shishijimicins A−C, novel enediyne antitumor antibiotics from the ascidian *Didemnum proliferum*. J. Am. Chem. Soc..

[61] Ohmoto T., Koike K., Brossi A. (1990). Chapter 3 Canthin-6-one Alkaloids. The Alkaloids: Chemistry and Pharmacology.

[62] Kam T.-S., Sim K.-M. (1998). Alkaloids from *Kopsia griffithii*. Phytochemistry.

[63] Wen-sen C. (1986). The isolation and structure of cordatanine from *Drymaria cordata* (L.) Willd. J. Integr. Plant Biol..

[64] Hsieh P.-W., Chang F.-R., Lee K.-H., Hwang T.-L., Chang S.-M., Wu Y.-C. (2004). A new anti-HIV alkaloid, drymaritin, and a new C-glycoside flavonoid, diandraflavone, from *Drymaria diandra*. J. Nat. Prod..

[65] Wetzel I., Allmendinger L., Bracher F. (2009). Revised structure of the alkaloid drymaritin. J. Nat. Prod..

[66] Zang Y., Genta-Jouve G., Zheng Y., Zhang Q., Chen C., Zhou Q., Wang J., Zhu H., Zhang Y. (2018). Griseofamines A and B: Two Indole-Tetramic Acid Alkaloids with 6/5/6/5 and 6/5/7/5 Ring Systems from Penicillium griseofulvum. Org. Lett..

[67] Pan X., Liu Z. (2019). Total synthesis and antibacterial activity evaluation of griseofamine A and 16-epi-griseofamine A. Org. Lett..

[68] Besslièvre R., Cosson B.P., Das B.C., Husson H.P. (1980). Structure and total synthesis of deplancheine, a novel indoloquinolizidine alkaloid. Tetrahedron Lett..

[69] Shi B.B., Chen J., Bao M.F., Zeng Y., Cai X.H. (2019). Alkaloids isolated from *Tabernaemontana bufalina* display xanthine oxidase inhibitory activity. Phytochemistry.

[70] Bao M.-F., Zeng C.-X., Liu Y.-P., Zhang B.-J., Ni L., Luo X.-D., Cai X.-H. (2017). Indole alkaloids from *Hunteria zeylanica*. J. Nat. Prod..

[71] Yan W., Ge H.M., Wang G., Jiang N., Mei Y.N., Jiang R., Li S.J., Chen C.J., Jiao R.H., Xu Q. (2014). Pictet-Spengler reaction-based biosynthetic machinery in fungi. Proc. Natl. Acad. Sci. USA.

[72] Khokhar S., Feng Y., Campitelli M.R., Ekins M.G., Hooper J.N.A., Beattie K.D., Sadowski M.C., Nelson C.C., Davis R.A. (2014). Isolation, structure determination and cytotoxicity studies of tryptophan alkaloids from an Australian marine sponge *Hyrtios* sp.. Bioorg. Med. Chem. Lett..

[73] Rath B., Hochmair M., Plangger A., Hamilton G. (2018). Anticancer activity of fascaplysin against lung cancer cell and small cell lung cancer circulating tumor cell lines. Mar. Drugs.

[74] Roll D.M., Ireland C.M., Lu H.S.M., Clardy J. (1988). Fascaplysin, an unusual antimicrobial pigment from the marine sponge *Fascaplysinopsis* sp.. J. Org. Chem..

[75] Segraves N.L., Robinson S.J., Garcia D., Said S.A., Fu X., Schmitz F.J., Pietraszkiewicz H., Valeriote F.A., Crews P. (2004). Comparison of fascaplysin and related alkaloids:  A study of structures, cytotoxicities, and sources. J. Nat. Prod..

[76] Asahina Y.J., Kashiwaki K. (1915). Chemical constituents of the fruits of *Evodia rutaecarpa*. Pharm. Soc. Jpn..

[77] Sun Q., Xie L., Song J., Li X. (2020). Evodiamine: A review of its pharmacology, toxicity, pharmacokinetics and preparation researches. J. Ethnopharmacol..

[78] Nasser A.M.A.G., Court W.E. (1984). Stem bark alkaloids of rauvolfia caffra. J. Ethnopharmacol..

[79] Majumdar S.P., Potier P., Poisson J. (1972). The structure of suaveoline, a new alkaloid from *Rauwolfia suaveolens* S. Moore (apocynaceae). Tetrahedron Lett..

[80] Amer M.M.A., Court W.E. (1980). Stem bark alkaloids of *Rauwolfia macrophylla*. Planta Med..

[81] Zhan G., Miao R., Zhang F., Hao X., Zheng X., Zhang H., Zhang X., Guo Z. (2020). Monoterpene indole alkaloids with diverse skeletons from the stems of *Rauvolfia vomitoria* and their acetylcholinesterase inhibitory activities. Phytochemistry.

[82] Xu Y.-J., Tang C.-P., Ke C.-Q., Ye Y. (2009). Indole alkaloids from leaves and stems of *Hunteria zeylanica*. Chem. Nat. Compd..

[83] Schnoes H.K., Biemann K., Mokry J., Kompis I., Chatterjee A., Ganguli G. (1966). Strictamine. J. Org. Chem..

[84] Ahmad Y., Fatima K., Atta ur R., Occolowitz J.L., Solheim B.A., Clardy J., Garnick R.L., Le Quesne P.W. (1977). Structure and absolute configuration of strictamine and strictalamine from *Rhazya stricta*. Stereochemistry of the *Picralima alkaloids*. J. Am. Chem. Soc..

[85] Zhang L., Zhang C.-J., Zhang D.-B., Wen J., Zhao X.-W., Li Y., Gao K. (2014). An unusual indole alkaloid with anti-adenovirus and anti-HSV activities from *Alstonia scholaris*. Tetrahedron Lett..

[86] Wong S.-P., Chong K.-W., Lim K.-H., Lim S.-H., Low Y.-Y., Kam T.-S. (2016). Arborisidine and arbornamine, two monoterpenoid indole alkaloids with new polycyclic carbon–nitrogen skeletons derived from a common pericine precursor. Org. Lett..

[87] Van Beek T.A., Verpoorte R., Baerheim Svendsen A. (1984). Alkaloids of *Tabernaemontana eglandulosa*. Tetrahedron.

[88] Gilani S.A., Kikuchi A., Shinwari Z.K., Khattak Z.I., Watanabe K.N. (2007). Phytochemical, pharmacological and ethnobotanical studies of *Rhazya stricta* Decne. Phytother. Res. Int. J. Devoted Pharmacol. Toxicol. Eval. Nat. Prod. Deriv..

[89] Kitajima M., Nakano S., Kogure N., Subhadhirasakul S., Takayama H. (2019). New indole alkaloids from *Ervatamia cumingiana* (Dedicated to Professor Tohru Fukuyama on the occasion of his 70th birthday). Heterocycles Int. J. Rev. Commun. Heterocycl. Chem..

[90] Ndongo J.T., Mbing J.N., Monteillier A., Tala M.F., Rütten M., Mombers D., Cuendet M., Pegnyemb D.E., Dittrich B., Laatsch H. (2018). Carbazole-, aspidofractinine-, and aspidocarpamine-type alkaloids from *Pleiocarpa pycnantha*. J. Nat. Prod..

[91] Zhang B.-J., Teng X.-F., Bao M.-F., Zhong X.-H., Ni L., Cai X.-H. (2015). Cytotoxic indole alkaloids from *Tabernaemontana officinalis*. Phytochemistry.

[92] Subramaniam G., Hiraku O., Hayashi M., Koyano T., Komiyama K., Kam T.-S. (2008). Biologically active aspidofractinine alkaloids from *Kopsia singapurensis*. J. Nat. Prod..

[93] Lim S.-H., Sim K.-M., Abdullah Z., Hiraku O., Hayashi M., Komiyama K., Kam T.-S. (2007). Leuconoxine, kopsinitarine, kopsijasmine, and kopsinone derivatives from *Kopsia*. J. Nat. Prod..

[94] Liu L., Chen Y.-Y., Qin X.-J., Wang B., Jin Q., Liu Y.-P., Luo X.-D. (2015). Antibacterial monoterpenoid indole alkaloids from *Alstonia scholaris* cultivated in temperate zone. Fitoterapia.

[95] Penelle J., Tits M., Christen P., Molgo J., Brandt V., Frédérich M., Angenot L. (2000). Quaternary indole alkaloids from the stem bark of Strychnos guianensis. Phytochemistry.

[96] Naaz H., Singh S., Pandey V.P., Singh P., Dwivedi U.N. (2013). Anti-cholinergic alkaloids as potential therapeutic agents for Alzheimer’s disease: An in silico approach. Indian J. Biochem. Biophys..

[97] Kitajima M., Anbe M., Kogure N., Wongseripipatana S., Takayama H. (2014). Indole alkaloids from *Kopsia jasminiflora*. Tetrahedron.

[98] Kam T.-S., Arasu L., Yoganathan K. (1996). Alkaloids from Kopsia pauciflora. Phytochemistry.

[99] Walser A., Djerassi C. (1965). Alkaloid-Studien LII. Die Alkaloide aus *Vallesia dichotoma*. Helv. Chim. Acta.

[100] Renner U. (1964). Alkaloide aus *Schizozygia Caffaeoides.* 3. Strukturelle Beziehungen zwischen Schizozygin und einigen Nebenalkaloiden. Lloydia.

[101] Wang K.-B., Di Y.-T., Bao Y., Yuan C.-M., Chen G., Li D.-H., Bai J., He H.-P., Hao X.-J., Pei Y.-H. (2014). Peganumine A, a β-carboline dimer with a new octacyclic scaffold from *Peganum harmala*. Org. Lett..

[102] Neuss N., Boaz H.E., Forbes J. (1954). Rauwolfia Serpentina alkaloids. I. Structure of reserpine. J. Am. Chem. Soc..

[103] Tsioufis C., Thomopoulos C. (2017). Combination drug treatment in hypertension. Pharmacol. Res..

[104] De Silva K.T.D., King D., Smith G.N. (1971). 5α-Carboxystrictosidine. J. Chem. Soc. D Chem. Commun..

[105] Ferrari F., Messana I., Botta B., de Mello J.F. (1986). Constituents of *Guettarda platypoda*. J. Nat. Prod..

[106] Kitajima M., Hashimoto K.-i., Yokoya M., Takayama H., Aimi N., Sakai S.-i. (2000). A New gluco indole alkaloid, 3,4-dehydro-5-carboxystrictosidine, from Preruvian Una de Gato (*Uncaria tomentosa*). Chem. Pharm. Bull..

[107] Onozawa T., Kitajima M., Kogure N., Peerakam N., Santiarworn D., Takayama H. (2017). A Cyclopeptide and a tetrahydroisoquinoline ílkaloid from *Ophiorrhiza nutans*. J. Nat. Prod..

[108] Brown R.T., Charalambides A.A. (1973). Adina alkaloids: The structure of rubenine. J. Chem. Soc. Chem. Commun..

[109] Wang Z.-X., Xiang J.-C., Cheng Y., Ma J.-T., Wu Y.-D., Wu A.-X. (2018). Direct biomimetic synthesis of β-carboline alkaloids from two amino acids. J. Org. Chem..

[110] Lood C., Nieger M., Koskinen A. (2015). Enantiospecific gram scale synthesis of (*S*)-eleagnine. Tetrahedron.

[111] Ellman J.A., Owens T.D., Tang T.P. (2002). *N*-tert-butanesulfinyl imines:  Versatile intermediates for the asymmetric synthesis of amines. Acc. Chem. Res..

[112] Reddy N.S.S., Babu R.A., Reddy B.V.S. (2016). Asymmetric synthesis of tetrahydro-β-carboline alkaloids employing Ellman’s chiral auxiliary. Synthesis.

[113] Van Maarseveen J.H., Hermkens P.H., De Clercq E., Balzarini J., Scheeren H.W., Kruse C.G. (1992). Antiviral and antitumor structure-activity relationship studies on tetracyclic eudistomines. J. Med. Chem..

[114] Roggero C.M., Giulietti J.M., Mulcahy S.P. (2014). Efficient synthesis of eudistomin U and evaluation of its cytotoxicity. Bioorg. Med. Chem. Lett..

[115] Kolodina A.A., OV S. (2018). Eudistomin U, isoeudistomin U, and related indole compounds: Synthesis and biological activity. Heterocycles.

[116] Kamal A., Sathish M., Prasanthi A.V.G., Chetna J., Tangella Y., Srinivasulu V., Shankaraiah N., Alarifi A. (2015). An efficient one-pot decarboxylative aromatization of tetrahydro-β-carbolines by using *N*-chlorosuccinimide: Total synthesis of norharmane, harmane and eudistomins. RSC Adv..

[117] Davis R.A., Carroll A.R., Quinn R.J. (1998). Eudistomin V, a New β-carboline from the australian ascidian *Pseudodistoma aureum*. J. Nat. Prod..

[118] Hati S., Sen S. (2016). *N*-Bromo-succinimide promoted synthesis of β-carbolines and 3,4-dihydro-β-carbolines from tetrahydro-β-carbolines. Tetrahedron Lett..

[119] Kamal A., Tangella Y., Manasa K.L., Sathish M., Srinivasulu V., Chetna J., Alarifi A. (2015). PhI(OAc)_2_-mediated one-pot oxidative decarboxylation and aromatization of tetrahydro-β-carbolines: Synthesis of norharmane, harmane, eudistomin U and eudistomin I. Org. Biomol. Chem..

[120] Pakhare D.S., Kusurkar R.S. (2015). Synthesis of tetrahydro-β-carbolines, β-carbolines, and natural products, (±)-harmicine, eudistomin U and canthine by reductive Pictet–Spengler cyclization. Tetrahedron Lett..

[121] Ábrányi-Balogh P., Földesi T., Grün A., Volk B., Keglevich G., Milen M. (2016). Synthetic study on the T3P^®^-promoted one-pot preparation of 1-substituted-3,4-dihydro-β-carbolines by the reaction of tryptamine with carboxylic acids. Tetrahedron Lett..

[122] Gaikwad S., Kamble D., Lokhande P. (2018). Iodine-catalyzed chemoselective dehydrogenation and aromatization of tetrahydro-β-carbolines: A short synthesis of kumujian-C, eudistomin-U, norharmane, harmane harmalan and isoeudistomine M. Tetrahedron Lett..

[123] Szabó T., Dancso A., Volk B., Milen M. (2021). First total synthesis of beta-carboline alkaloid trigonostemine G and its derivatives. Nat. Prod. Res..

[124] Szabó T., Hazai V., Volk B., Simig G., Milen M. (2019). First total synthesis of the β-carboline alkaloids trigonostemine A, trigonostemine B and a new synthesis of pityriacitrin and hyrtiosulawesine. Tetrahedron Lett..

[125] Ambule M.D., Tripathi S., Ghoshal A., Srivastava A.K. (2019). IBX-mediated oxidative addition of isocyanides to cyclic secondary amines: Total syntheses of alangiobussine and alangiobussinine. Chem. Commun..

[126] Singha Roy M., Meng X., Koda K., Rasapalli S., Gout D., Lovely C.J. (2019). Total synthesis of (−)-haploscleridamine. Tetrahedron Lett..

[127] Kulkarni A.S., Shingare R.D., Dandela R., Reddy D.S. (2018). Total synthesis of an anticancer natural product (±)-peharmaline A and its analogues. Eur. J. Org. Chem..

[128] Wang Z., Niu J., Zeng H., Li C.-J. (2019). Construction of spirocyclic tetrahydro-β-carbolines *via* cross-annulation of phenols with tryptamines in water. Org. Lett..

[129] Meng T.-Z., Zheng J., Trieu T.H., Zheng B., Wu J.-J., Zhang Y., Shi X.-X. (2018). CuBr_2_-Catalyzed mild oxidation of 3,4-dihydro-β-carbolines and application in total synthesis of 6-hydroxymetatacarboline D. ACS Omega.

[130] Nicolaou K., Lu Z., Li R., Woods J.R., Sohn T.-i. (2015). Total synthesis of shishijimicin A. J. Am. Chem. Soc..

[131] Schott Y., Decker M., Rommelspacher H., Lehmann J. (2006). 6-Hydroxy- and 6-methoxy-β-carbolines as acetyl- and butyrylcholinesterase inhibitors. Bioorg. Med. Chem. Lett..

[132] Cogan D.A., Liu G., Ellman J. (1999). Asymmetric synthesis of chiral amines by highly diastereoselective 1,2-additions of organometallic reagents to *N*-tert-butanesulfinyl imines. Tetrahedron.

[133] Davis F.A., Gaddiraju N.V., Theddu N., Hummel J.R., Kondaveeti S.K., Zdilla M.J. (2012). Enantioselective synthesis of cocaine C-1 analogues using sulfinimines (*N*-sulfinyl imines). J. Org. Chem..

[134] Nishikawa Y., Kitajima M., Kogure N., Takayama H. (2009). A divergent approach for the total syntheses of cernuane-type and quinolizidine-type Lycopodium alkaloids. Tetrahedron.

[135] Fandrick D.R., Hart C.A., Okafor I.S., Mercadante M.A., Sanyal S., Masters J.T., Sarvestani M., Fandrick K.R., Stockdill J.L., Grinberg N. (2016). Copper-catalyzed asymmetric propargylation of cyclic aldimines. Org. Lett..

[136] Nalikezhathu A., Cherepakhin V., Williams T.J. (2020). Ruthenium Catalyzed Tandem Pictet–Spengler Reaction. Org. Lett..

[137] Shelar S.V., Argade N.P. (2017). Total synthesis of bioactive canthine alkaloid cordatanine comprising in situ double oxidative aromatization of tetrahydrocarbazole. ACS Omega.

[138] Fang H.W., Liao Y.-R., Hwang T.-L., Shieh P.-C., Lee K.-H., Hung H.-Y., Wu T.-S. (2015). Total synthesis of cordatanine, structural reassignment of drymaritin, and anti-inflammatory activity of synthetic precursors. Bioorg. Med. Chem. Lett..

[139] Zheng Y., Wei K., Yang Y.-R. (2017). Total synthesis of (−)-geissoschizol through Ir-catalyzed allylic amidation as the key step. Org. Lett..

[140] Shi Y., Xu Z., Tan R., Lei X. (2019). Divergent total synthesis of chaetoglines C to F. J. Org. Chem..

[141] Zhidkov M.E., Kantemirov A.V., Koisevnikov A.V., Andin A.N., Kuzmich A.S. (2018). Syntheses of the marine alkaloids 6-oxofascaplysin, fascaplysin and their derivatives. Tetrahedron Lett..

[142] Wang Z.-X., Xiang J.-C., Wang M., Ma J.-T., Wu Y.-D., Wu A.-X. (2018). One-Pot Total Synthesis of evodiamine and its analogues through a continuous biscyclization reaction. Org. Lett..

[143] Zhao Z., Wei H., Xiao K., Cheng B., Zhai H., Li Y. (2019). Facile Synthesis of pyridines from propargyl amines: Concise total synthesis of suaveoline alkaloids. Angew. Chem..

[144] Zhang L.-H., Bi Y.-Z., Yu F.-X., Menzia G., Cook J. (1992). Stereospecificity in the Pictet–Spengler reaction. Enantiospecific synthesis of (6S,10S)--(–)-5-methyl-9-oxo-12-benzyl-6,7,8,9,10,11-hexahydro-6,10-imino-5*H*-cyclooct[*b*]indole, a template for preparation of macroline/sarpagine alkaloids. Heterocycles.

[145] Fu X., Cook J.M. (1993). General approach for the synthesis of ajmaline-related alkaloids. Enantiospecific total synthesis of (-)-suaveoline, (-)-raumacline, and (-)-Nb-methylraumacline. J. Org. Chem..

[146] Smith M.W., Zhou Z., Gao A.X., Shimbayashi T., Snyder S.A. (2017). A 7-step formal asymmetric total synthesis of strictamine *via* an asymmetric propargylation and metal-mediated cyclization. Org. Lett..

[147] Ren W., Wang Q., Zhu J. (2016). Total synthesis of (±)-strictamine. Angew. Chem. Int. Ed..

[148] Nishiyama D., Ohara A., Chiba H., Kumagai H., Oishi S., Fujii N., Ohno H. (2016). Formal total synthesis of (±)-strictamine based on a gold-catalyzed cyclization. Org. Lett..

[149] Kolundžić F., Murali A., Pérez-Galán P., Bauer J.O., Strohmann C., Kumar K., Waldmann H. (2014). A cyclization–rearrangement cascade for the synthesis of structurally complex chiral gold(I)–aminocarbene complexes. Angew. Chem. Int. Ed..

[150] Zheng Y., Yue B.-B., Wei K., Yang Y.-R. (2018). Short synthesis of the monoterpene indole alkaloid (±)-arbornamine. J. Org. Chem..

[151] Szántay C.N. (1994). The Chemistry of Heterocyclic Compounds.

[152] Vas Á., Gulyás B. (2005). Eburnamine derivatives and the brain. Med. Res. Rev..

[153] Massiot G., Oliveira F., Lévy J. (1982). Synthesis in the indole series. 9. Total synthesis of the pseudovincamones. Bull. Soc. Chim. Fr. Partie 2.

[154] Smith M.W., Ferreira J., Hunter R., Venter G.A., Su H. (2019). Synthesis of (+)-tacamonine *via* stereoselective radical cyclization. Org. Lett..

[155] Jarret M., Tap A., Kouklovsky C., Poupon E., Evanno L., Vincent G. (2018). Bioinspired oxidative cyclization of the geissoschizine skeleton for the total synthesis of (−)-17-nor-excelsinidine. Angew. Chem. Int. Ed..

[156] Maxime J., Victor T., Aurélien T., Jean-Francois G., Cyrille K., Erwan P., Guillaume V., Laurent E. (2019). Bioinspired oxidative cyclization of the geissoschizine skeleton for the enantioselective total synthesis of mavacuran alkaloids. Angew. Chem. Int. Ed..

[157] Sato K., Kogure N., Kitajima M., Takayama H. (2019). Total syntheses of pleiocarpamine, normavacurine, and C-mavacurine. Org. Lett..

[158] Trost B.M., Bai Y., Bai W.-J., Schultz J.E. (2019). Enantioselective divergent synthesis of C19-oxo eburnane alkaloids *via* palladium-catalyzed asymmetric allylic alkylation of an *N*-alkyl-α,β-unsaturated lactam. J. Am. Chem. Soc..

[159] Baran P.S., Guerrero C.A., Corey E. (2003). Short, enantioselective total synthesis of okaramine N. J. Am. Chem. Soc..

[160] White K.L., Mewald M., Movassaghi M. (2015). Direct observation of intermediates involved in the interruption of the Bischler–Napieralski reaction. J. Org. Chem..

[161] Zhang X., Anderson J.C. (2019). A divergent synthetic route to the vallesamidine and schizozygine alkaloids: Total synthesis of (+)-vallesamidine and (+)-14,15-dehydrostrempeliopine. Angew. Chem. Int. Ed..

[162] Evans D.A., Seidel D. (2005). Ni(II)−Bis[(*R*,*R*)-*N*,*N*‘-dibenzylcyclohexane-1,2-diamine]Br_2_ catalyzed enantioselective Michael additions of 1,3-dicarbonyl compounds to conjugated nitroalkenes. J. Am. Chem. Soc..

[163] Piemontesi C., Wang Q., Zhu J. (2016). Enantioselective synthesis of (+)-peganumine A. J. Am. Chem. Soc..

[164] Woodward R., Bader F., Bickel H., Frey A., Kierstead R. (1958). The total synthesis of reserpine. Tetrahedron.

[165] Khan Z.A., Shahzad S.A., Anjum A., Bale A.T., Naqvi S.A.R. (2018). Synthetic approaches toward the reserpine. Synth. Commun..

[166] Park J., Chen D.Y. (2018). A desymmetrization-based total synthesis of reserpine. Angew. Chem. Int. Ed. Engl..

[167] Rakumitsu K., Sakamoto J., Ishikawa H. (2019). Total Syntheses of (−)-Secologanin,(−)-5-Carboxystrictosidine, and (−)-Rubenine. Chem. A Eur. J..

[168] Takayama H., Fujiwara R., Kasai Y., Kitajima M., Aimi N. (2003). First asymmetric total synthesis of Us-7 and -8, novel D-seco corynanthe-type oxindole alkaloids from *Uncaria a ttenuata*: Structure revision of Us-7 and determination of absolute stereochemistry. Org. Lett..

[169] Tietze L.F., Meier H., Nutt H. (1990). Inter- and intramolecular hetero Diels-Alder reactions, 28. Synthesis of (±)-secologanin aglucone O-ethyl ether and derivatives by tandem Knoevenagel hetero Diels-Alder reaction. Liebigs Ann. Chem..

[170] Bailey P.D., Hollinshead S.P., McLay N.R. (1987). Exceptional stereochemical control in the Pictet–Spengler reaction. Tetrahedron Lett..

